# iCAVE: an open source tool for visualizing biomolecular networks in 3D, stereoscopic 3D and immersive 3D

**DOI:** 10.1093/gigascience/gix054

**Published:** 2017-07-15

**Authors:** Vaja Liluashvili, Selim Kalayci, Eugene Fluder, Manda Wilson, Aaron Gabow, Zeynep H. Gümüş

**Affiliations:** 1 Department of Genetics and Genomic Sciences, Icahn School of Medicine at Mount Sinai, New York, NY 10029, USA; 2 Icahn Institute for Genomics and Multiscale Biology, Icahn School of Medicine at Mount Sinai, New York, NY 10029, USA; 3 Computational Biology Center, Memorial-Sloan Kettering Cancer Center, New York, NY 10065, USA

**Keywords:** biomolecular networks, network visualization, visualization, immersive, stereoscopic, CAVE

## Abstract

Visualizations of biomolecular networks assist in systems-level data exploration in many cellular processes. Data generated from high-throughput experiments increasingly inform these networks, yet current tools do not adequately scale with concomitant increase in their size and complexity. We present an open source software platform, interactome-CAVE (iCAVE), for visualizing large and complex biomolecular interaction networks in 3D. Users can explore networks (i) in 3D using a desktop, (ii) in stereoscopic 3D using 3D-vision glasses and a desktop, or (iii) in immersive 3D within a CAVE environment. iCAVE introduces 3D extensions of known 2D network layout, clustering, and edge-bundling algorithms, as well as new 3D network layout algorithms. Furthermore, users can simultaneously query several built-in databases within iCAVE for network generation or visualize their own networks (e.g., disease, drug, protein, metabolite). iCAVE has modular structure that allows rapid development by addition of algorithms, datasets, or features without affecting other parts of the code. Overall, iCAVE is the first freely available open source tool that enables 3D (optionally stereoscopic or immersive) visualizations of complex, dense, or multi-layered biomolecular networks. While primarily designed for researchers utilizing biomolecular networks, iCAVE can assist researchers in any field.

## Introduction

Interaction networks are one of the primary visual metaphors for communicating and understanding -omics data at a systems level. From cellular organisms to human society, networks provide critical clues on systems-level behavior [1–3]. In biomedicine, they are essential for understanding normal [4, 5] and disease states [6–9], and they are instrumental for drug discovery [10–12] and biomarker identification [13–15]. Changes in networks have helped in prognosis for breast cancer patients [6], analyzing systematic inflammation in humans [8] or studying emerging tumor markers [16]. Network visualizations are thus important in basic and translational biomedical research, with an abundance of tools for their exploration [17, 18]. Many tools are also coupled with public databases, enabling visualizations in the context of previous knowledge [17]. In fact, currently more than 500 resources are listed at Pathguide, [19] with thousands of networks and millions of biomolecular interactions [20].

Among currently available tools, Cytoscape [21] and Gephi [22] are quite popular. There are also a number of JavaScript network visualization libraries (e.g., sigma.js [23]), and software packages (e.g., iGraph [24]) on the web. However, the layout algorithms in these libraries and employed in Cytoscape [25], in addition to other tools like Ingenuity [26], Osprey [27], VisANT [28], and BINA [29], to name a few, are limited by the number of molecules and interactions that can be displayed on a 2D screen, and the associated layout and representation challenges. Furthermore, recent technological developments have increased the size and complexity of -omics experimental data, with simultaneous recordings from multiple cellular events leading to unprecedented growth in interaction data [30]. New approaches are necessary to address the visualization design challenges that the concomitant large, complex, and multi-dimensional (multi-layered) networks present to explore these systems.

Currently networks with 3D localizations (e.g., brain connectivity networks or molecular complexes) are best explored in 3D. For representing abstract data, taking advantage of the third dimension can also allow for greater freedom; however, the available 3D visualization options and tools are still somewhat nascent in this domain. Stereoscopy, the projection of separate images to each eye, which creates the illusion that virtual objects have volumes in 3D space, has been shown to be particularly beneficial for exploring large, complex networks, either alone or combined with rotation or user motion cues [31–33]. Immersive visualization environments, where users are virtually immersed inside the image, have also led to better performance than 2D in user studies for relatively difficult tasks and large networks [34]. For example, a CAVE environment, which includes projectors directed to several walls of a room-sized cube to create the sense of presence inside a virtual world, has been shown to help identify a new network property that a 2D display failed [35]. The technology features that were particularly helpful were stereoscopy, magnification, and wide field of view (the extent of the visualized image observed by the user) [35]. However, a CAVE facility is a substantial investment to build and maintain that only a limited number of institutions have. Network visualizations in stereoscopic and immersive 3D environments are still new, and we currently do not have community tools to help us understand how best to use them by conducting user studies on different technology platforms and testing alternative layout algorithms or to explore phenomena that involve large, complex networks.

Here, we introduce interactome-CAVE (iCAVE), an open source tool for 3D, stereoscopic 3D, and immersive 3D visualizations of complex, large, and/or multi-layered networks. iCAVE development is made possible by the continuous evolution of data analysis tools in VR, stereoscopic visualization, and emerging 3D technologies. Use of VR technology in life sciences research is still nascent [36–39], and so far does not include free open source tools for biomolecular network visualizations, mainly due to the limited portability of the technology to personal computers until recently. iCAVE is completely portable, taking advantage of recent advances in computer graphics hardware, software, and content creation that are leading to a proliferation of stereoscopic visualization capabilities in personal computing. Computers can now be upgraded to display high-quality stereoscopic 3D visuals with low-cost stereoscopic 3D glasses and software [40], which are much cheaper than recent head-mounted displays (HMDs). As most scientific computers are becoming stereo enabled and 3D glasses are going mainstream, iCAVE is on the leading edge of this larger trend in the evolution of visual computing technology. If a computer is equipped with stereo capabilities, users can explore stereoscopic 3D visualizations. With large screens or curved walls, users can additionally take advantage of magnification and wide field of view. Users can also use iCAVE in immersive CAVE environments. Without a CAVE or a stereo-equipped computer (or if users choose to turn off stereo), iCAVE provides interactive 3D visualizations that still offer most of its features.

Note that while few network visualization tools incorporate 3D layouts [41–43], they are not immersive 3D, i.e., they do not have interoperation capability with virtual reality (VR) technologies, and have 2D displays. For example, Arena 3D [41] mixes 3D and 2D properties by arranging data in multilayered graphs in 2D, with each layer representing a different data type. While the tool includes several layout and clustering algorithms for each layer and has zoom and rotation features, it does not offer global layout and clustering algorithms to make full use of the third dimension, and each layer is in 2D [41]. 3DScapeCS [42] is a Cytoscape PlugIn written in Java, with built-in extensions of the classic 2D force-directed layouts. Users cannot add new layouts or functionalities, and it does not utilize 3D effects to improve comprehension (e.g., transparency or advanced shadow effects). BioLayoutExpress (now Miru) [43] is a standalone 3D application specifically designed for gene expression networks that offers three network layouts, a clustering method, no edge bundling, and limited network topology statistics. Importantly, it is not freely available. In summary, 3D biomolecular network visualization is a nascent field. We need free open source tools for biologists to visualize their networks and for algorithm developers to add and test new methods that take advantage of the third dimension. Such a tool will also enable visualization designers to perform user studies to better understand the relative advantages of various 3D features. This is necessary as how best to utilize features specific to 3D or to take advantage of new 3D technologies is currently an open research question.

To the best of our knowledge, iCAVE is the first 3D, stereoscopic 3D, and immersive 3D biomolecular network visualization tool that is open source, freely available, and utilizable with commercial hardware/software. iCAVE introduces new built-in 3D algorithms for laying out nodes and their connections in 3D space and has built-in topology-based graph clustering algorithms. For example, it enables visual integration of multiple clusters or data types within the same graph as a multi-layered network (e.g., metabolomic, proteomic, genomic, genome-wide association studies (GWAS) disease, protein-drug interactions). Users can also add their own layout or clustering algorithms. While not extensive, it includes a few built-in databases to assist in preliminary mapping of high-throughput (HT) experimental data in the early discovery phase of network building. Customizable color, texture, size, and layout options assist in displaying maximum information in a graph in an optimized manner. Users can easily select edge colors, weights, and directions or bundle edges for simplified views. Data are input in a tab-delimited text file while visual outputs can be saved in 2D snapshots or movies configured with user-defined rotation, zoom, and speeds. Additional reports on network statistics are provided in 2D. Overall, iCAVE enables network explorations in hypothesis-driven contexts that are flexible, collaborative, and user friendly.

In the following section, we first discuss our main contributions and findings on visualizing networks that are large, with or without known 3D physical coordinates, and with multiple data types using iCAVE. We then introduce the algorithms we implemented in iCAVE for network layout and clustering and discuss input and output formats, as well as performance and scalability aspects. Then, we summarize the features of iCAVE in the Discussion section. Finally, in the Methods section, we provide details on software libraries, user interface, network topology statistics, and layout algorithms.

## Results

### Optional stereoscopy

**Figure 1:**
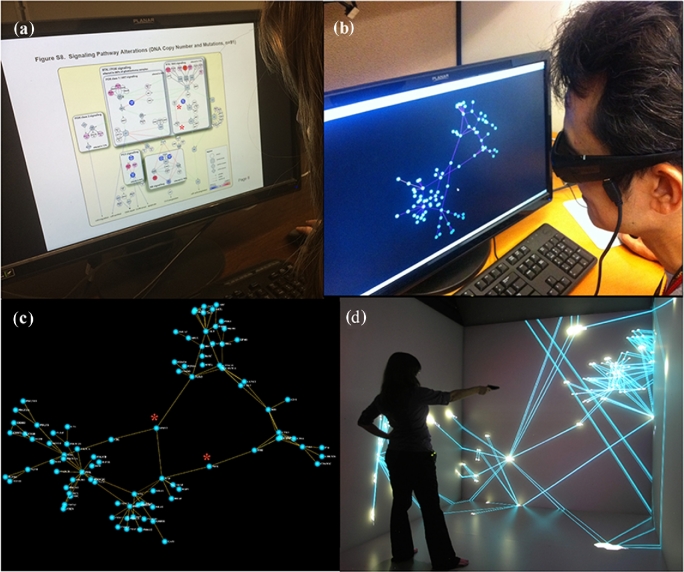
Comparison of displays. (**a**) User interacting with a flat 2D display of manually curated pathways affected by genomic alterations in glioblastoma [44]. (**b**) User experiencing full 3D depth perception of the same network with iCAVE using stereoscopic glasses on his desktop. iCAVE display is generated with a force-directed layout algorithm. The visual clutter problem due to edge crossings that create a hairball effect in 2D is eliminated as the user can navigate to multiple viewpoints. (**c**) A screenshot from the 3D display generated with the force-directed layout. This network is generated without *a priori* knowledge of the underlying biology; rotating the layout helps readily identify hubs, connectors, and modules, such as the connectors between 2 dense regions of the network (highlighted with an asterisk in both (a and c). (**d**) Immersive visualization in a CAVE environment, with user inside data space at Weill Cornell 3D CAVE^TM^ facility in New York. While the photos only capture images reflected on the interaction walls of the CAVE, the user experiences a virtual 3D image. In both (b and d), zoom and rotation options help users focus on a particular hub or module. While the addition of the third dimension gives a richer, more intuitive, and ultimately more meaningful understanding of the network-represented data, the 3D layout brings a new modality into network visualization design, with clear layouts.

iCAVE users can turn 3D stereoscopy on or off during exploration. For example, consider rendering the 2D biomolecular network in Fig. 1A that represents a pathway affected by genomic alterations in glioblastoma [44]. Instead of the static 2D network in Fig. 1A, users can experience full 3D depth perception at the comfort of their own stereo-equipped computer (Fig. 1B), or inside a CAVE (Fig. 1D) using a simple 3D extension of a classical force-directed layout algorithm (Fig. 1) [45]. Even users without a stereo-equipped computer can interact with the 3D network: they can use their mouse (in lieu of hand-held controls) to zoom in/out or rotate the network to a view without occlusions. Rotation and zoom enables viewing the network from different view angles, such as the screenshot in Fig. 1C. User studies have shown that even simple 3D features like rotation help better identification of properties unique to complex networks [33]. Visualizations using stereoscopic 3D or immersive environments that enable inspection of a system from multiple perspectives have also been shown to make different properties of a system clearer [46]. Our case study supports this as we observed a network feature that was not intuitive from the original 2D layout in Fig. 1A: nodes CBL and SPRY2 (with *) are “connectors” between 2 dense network regions (modules) (Fig. 1A–C). A targeted attack to these genes can splits the network into two. We could not identify this in 2D. Such discoveries of network topological features, among others, give a richer, more intuitive, and ultimately more insightful understanding of networks.

### Addressing large networks

Important characteristics may be missed if users cannot interact with the complete network. In the simplest case, the nodes may form (i) dense sub-networks that are interconnected by a small number of “connector” nodes that render them critical or (ii) multiple networks (often 1 giant and few smaller networks) where the smaller sub-networks may represent functional groups of importance, such as a critical enzyme complexes. Hence, visualizing the complete network can be advantageous even if it is very large to identify local patterns [47]. However, while the human brain has a remarkable capacity to visually identify patterns, enabling interpretation of data, visualizations of large networks may exhibit problems with display clutter, molecular positioning, or perceptual tension, leading the user to misinterpret closely positioned molecules as related [48]. Such misinterpretations are inherent in the limitations of human visual perception and have been well-studied in (Gestalt) psychology: people tend to organize visual elements into groups [49].

Three-dimensional elements that appear to form a pattern because of their visual positioning in one viewpoint can be interpreted correctly by rotating the image to a different viewpoint (e.g., Fig. 1). Furthermore, in networks that are denser or larger than that of Fig. 1, the potential 2D “hairball” effect can obscure important interactions. iCAVE users can simply navigate to a view without occlusions by moving their head, rotating the image, and zooming in or out, eliminating “edge-crossings.” To further address cluttering, iCAVE provides an “edge-bundled display” [50] option for visually bundling adjacent edges together, analogous to bundling electrical wires or cables. Bundling is extremely useful in identifying global patterns in very large networks and can suggest vulnerabilities as targets. Several layout algorithms built-in within iCAVE address the molecular positioning problem; depending on the topology of a network, one may work better than another. We suggest testing each to see which works best. We provide examples of how these features can help with exploring a network in the following sections.

### New biological insights from networks with known 3D physical coordinates

**Figure 2:**
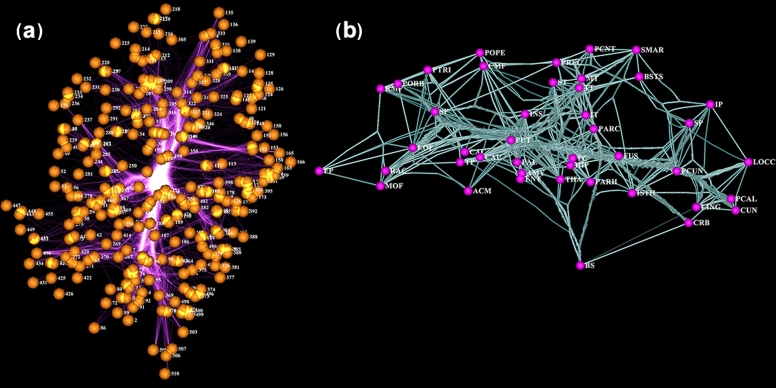
(**a**) iCAVE visualization of bacterial leucine transporter, LeuT residue correlation network, side-view. Nodes represent 3D coordinates of alpha-carbon of a residue; edges represent top 3,000 (Pearson) correlations between residue pairs, where the input is 3D coordinates and correlation scores. Surprisingly, 3D visualization with edge bundling enables representation of highest-density correlations (“correlation highways”) that travel through the substrate permeation pore in the protein center, connecting extracellular and intracellular domains. Correlation highways at the pore are visually fascinating and biophysically intuitive; some residues outside the pore reveal unexpected structural importance (data courtesy of Michael LeVine and Harel Weinstein). (**b**) Living human brain connectivity. iCAVE visualization of brain regions as nodes, labeled by anatomical region name. Edges show connectivity, and bundling shows “connectivity highways.” Datasets from diffusion tensor imaging of the left hemisphere scanned ith Siemens 1.5Tesla and generated by fiber assignment by continuous tracking tractography using the University of California, Los Angeles, Multimodal Connectivity Package, connectivity matrix module. The database is powered by the Human Connectome Project, which compiles neural data to achieve never before realized conclusions on the living human brain.

Users can visualize physically constrained networks at multiple scales, from proteins (Fig. 2A) to the whole brain (Fig. 2B). Coupled with edge bundling, these can provide insights in hypothesis generation. For example, Fig. 2A represents a snapshot of bacterial leucine transporter (LeuT) residue correlation network, where nodes represent 3D coordinates of alpha-carbon of a residue and edges represent top 3,000 (Pearson) correlations between residue pairs from a Molecular Dynamics simulation (Michael LeVine, personal communication). Remarkably, bundling the edges of this network enables the representation of highest-density “correlation highways” that travel through the substrate permeation core in the protein center, connecting extracellular and intracellular domains. These highways enable users to identify specific residues that have dense correlations with the permeation core even if they are away from it, which is unexpected. These residues may have previously unidentified importance in protein structure and function and are therefore potential candidates for follow-up studies.

### Utilizing the third dimension for automated 3D layout positioning in abstract networks

Biomolecular networks tend to follow basic and reproducible organizing principles, and navigating the entire network provides a good initial understanding. The layout algorithm must address the complex problem of arranging the nodes to clearly disseminate the network topology, and at the same time be visually pleasant and user friendly. iCAVE offers several network layout options to achieve these aims:

Due to user familiarity, we extended variations of the force-directed layout to 3D: (i) the classical “force-directed algorithm” [45] treats the network as a physical system with edges analogous to “springs” and nodes to “electrically charged particles” that repel each other, where the final layout is established when the repulsive and attractive forces balance each other [51]; (ii) “hybrid force-directed layout” [52] partitions the graph into smaller units prior to applying the force-directed algorithm; (iii) “lin-log layout” [53] is better suited for larger networks as it keeps highly connected nodes in close proximity with minimal number of edge crossings. Alternatively, for larger networks, (iv) “coarsened force-directed layout” combines a force-directed algorithm with an efficient, high-quality force-directed graph drawing graph coarsening technique [54]; and (v) “simulated annealing force-directed layout” uses simulated annealing to rapidly scale to very large networks (see the Methods section) [55].

We further implemented 2 novel layout algorithms to take full advantage of immersive 3D features:

The “semantic levels layout algorithm” segregates the network into separate layers (default 7) in the third dimension. The layout of each layer is calculated with a 3D extension of the force-directed approach. Semantic layers layout can be especially useful for user-defined networks where the number of layers and node assignments to layers can correspond to different data types (e.g., a 2D projection in Fig. 4 and 3D video in Supplementary Video 3, with layer1: genes; layer2: diseases; layer3: drugs).

“Hemispherical layout” is a novel layout algorithm we have developed that positions the network on the surface of a 3D hemisphere. The most connected node is positioned at the top center of the hemisphere. Then, the whole hemisphere surface is populated based on a decreasing rank order of connectivity. The node positions are fixed, and the edges are drawn on the hemisphere surface (e.g., see a 2D projection in Fig. 5C and 3D video in Supplementary Video 4).

Each layout algorithm has unique strengths, and we recommend that the user test different options. Semantic layout is often ideal for hierarchical networks. Force-directed layout often captures the essence of large networks. Hemispherical layout leads to clean images with optional edge bundling (Fig. 5C; Supplementary Video 4).

### Illustrative examples on network layouts

#### Example 1

**Figure 3:**
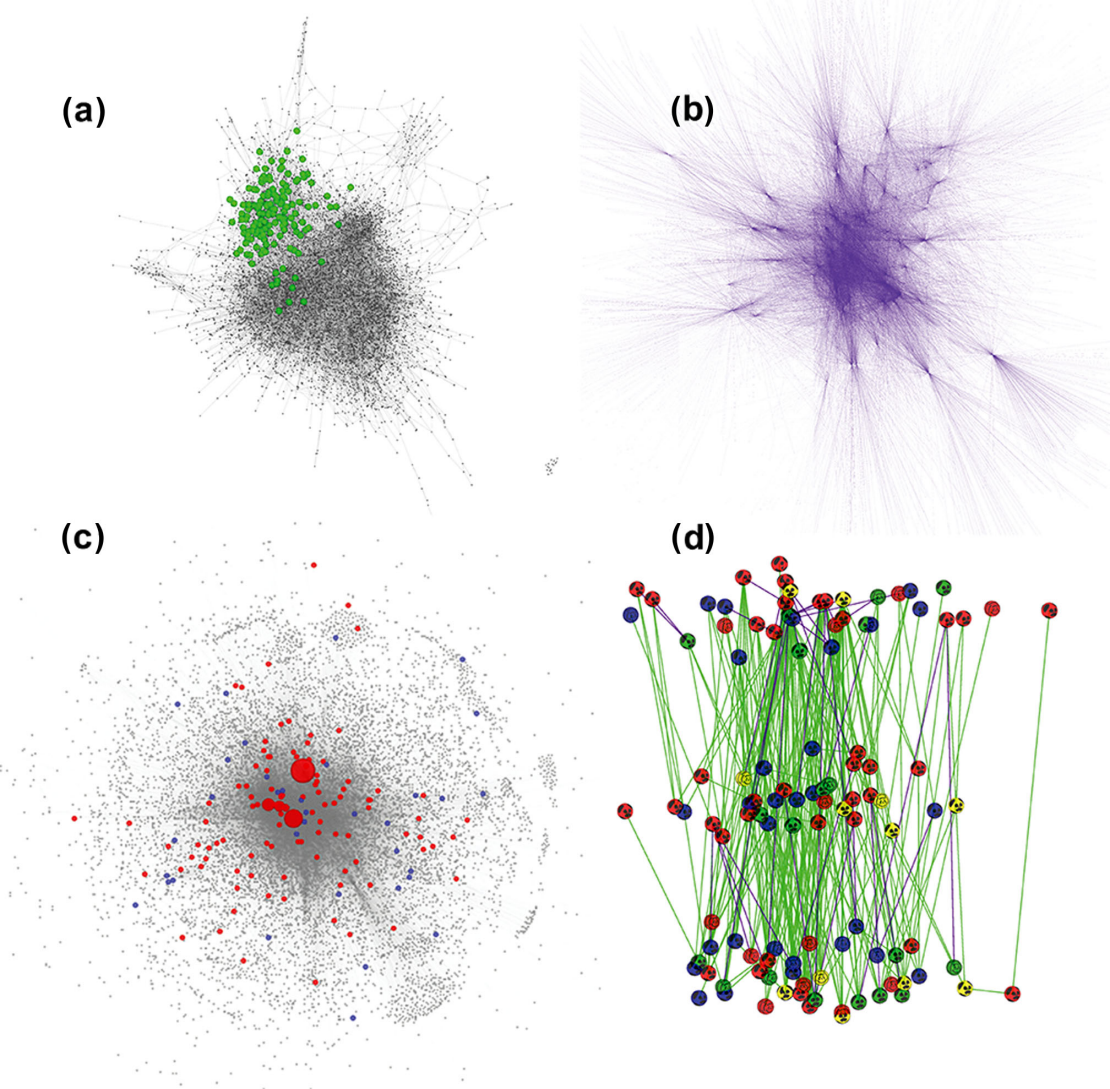
iCAVE print-ready images of networks in 2D with a white background. (**a**) A large probabilistic causal network constructed from human omental adipose tissue in a morbidly obese patient cohort (7,601 nodes, 13,979 edges) [56]. Nodes are gene expression traits in tissue; edges are derived from a Bayesian network reconstruction algorithm that leverages DNA variation for causality. Highlighted nodes represent the gene signature causally associated with disease variants or pathways. Signature genes cluster together, suggesting functional relatedness. (**b**) Network of 119 TFs, their 26,037 target interactions (edges) with 9,057 genes (nodes) [58] from the ENCODE study. (**c**) Massive unified “multinet” of protein–protein interaction, phosphorylation, metabolic, signaling, genetic, and regulatory networks (14 558 nodes, 109,597 edges). Multinet correlates tolerance to LoF mutations and evolutionary conservation, with nodes for (LoF) tolerant (blue) and essential genes (red) easily distinguishable. Node size is based on the degree of centrality of a gene. Essential genes tend to be bigger and central, and LoF-tolerant genes are smaller in the periphery. (**d**) Hierarchical network integrates TF, ncRNA, miRNA, and protein–protein interaction data. Hierarchy levels are based on the mutual relationships between TFs. Connectivity and hierarchy reflects genomic properties (top-level TF-binding correlates with target expression; mid-level contains information flow bottlenecks and connections with miRNA and distal regions, revealing ideal drug targets) (Mark Gerstein, personal communication). While a 2D figure cannot display the interconnections between elements within the same hierarchical level, it is straightforward with iCAVE semantic levels layout.

Visualizing the complete global network, even if it is very large, can enable visual identification of a pattern. For example, consider a large probabilistic causal network constructed from human omental adipose tissue in a morbidly obese patient cohort in Fig. 3A. The network consists of 7,601 nodes, 13,979 edges [56]. Nodes are the genes expressed in tissue; edges are derived from a Bayesian network reconstruction algorithm that leverages DNA variation for causality. Here, we highlight nodes that represent a signature of genes causally associated with inflammatory bowel disease (IBD) single nucleotide polymorphisms or disease pathways. Notice that within this global view of the massive network, there is a pattern of the IBD genes clustering together, which visually supports the hypothesis of functional relatedness.

#### Example 2

While force-directed layout algorithms can help identify global patterns, if the interaction network has a hierarchy, semantic layers layout can help visualize the hierarchical nature of the interactions easily. For example, Fig. 3B displays the global view of a network generated from The Encyclopedia of DNA Elements (ENCODE) [57] study data. The ENCODE Consortium is generating a comprehensive parts list of the human genome functional elements, including those that control active genes, such as transcription factors (TFs). Utilizing these unprecedented volumes of data, Gerstein and co-workers have generated the massive network in Fig. 3B that includes 119 TFs that target 9,057 genes (nodes) via 26 037 interactions (edges) [58]. Using force-directed layouts, users can capture the general network structure and differentiate a TF from its neighbors by zooming in/out, adding labels to that specific TF, etc., and users obtain statistics on network centrality and other global topological properties as they pertain to the network. However, the semantic layers layout is useful in visualizing the hierarchical nature of this network, integrating TF, non-coding RNA (ncRNA), miRNA, and protein–protein interaction data (Fig. 3D; Supplementary Video 2). Here, network connectivity and hierarchy reflect genomic properties: top-level TF binding correlates with target expression, and mid-level contains “information flow bottlenecks” and connections with miRNA and distal regions, revealing ideal drug targets. Such multi-layered heterogeneous information integration assists in differentiating intra-level interconnections as well as inter-level edge types and node labels. Note that nodes in each layer are also arranged in 3D using 3D force-directed layout.

#### Example 3

Visualizing the global network of interactions while scaling or coloring a subset of the nodes based on their specific properties can enable hypothesis support. In this example, the visualization helps support the principle that functionally significant and highly conserved genes tend to be more central in physical protein–protein and regulatory networks [59]. Based on this hypothesis, Fig. 3C visualizes a network of tolerance to loss-of-function (LoF) mutations and evolutionary conservation, with nodes for (LoF) tolerant (blue) and essential genes (red) easily distinguishable [59]. Node size is based on degree of centrality of a gene. While essential genes tend to be bigger and central, LoF-tolerant genes are smaller and located in the periphery. Both the 2D snapshot (Fig. 3C) and 3D Supplementary Video 1 provide clear visualizations of this complex data that lead to easy interpretation. Note that we have published an iCAVE-generated visualization of a network with similar properties that helped to support this hypothesis [60].

### Multiple data types

#### COMBO database for simultaneous query of multiple data types

Publicly available biomolecular interaction data are often contained in massive databases [19]. While not comprehensive, iCAVE combines data from multiple resources into a single COMBO repository to enable quick queries. These include the protein–protein interaction databases Human Protein Reference Database [61] and Intact [62], the disease and associated gene variants GWAS database [63], and drug-target databases STITCH [64] and DRUGBANK [65]. Pathways database SuperPathway is stored separately (Josh Stuart, personal communication). Users can add their own databases without affecting other parts of the code. Details on the COMBO database are given in Supplementary Table S1.

#### Visualizing multiple layers of data

**Figure 4:**
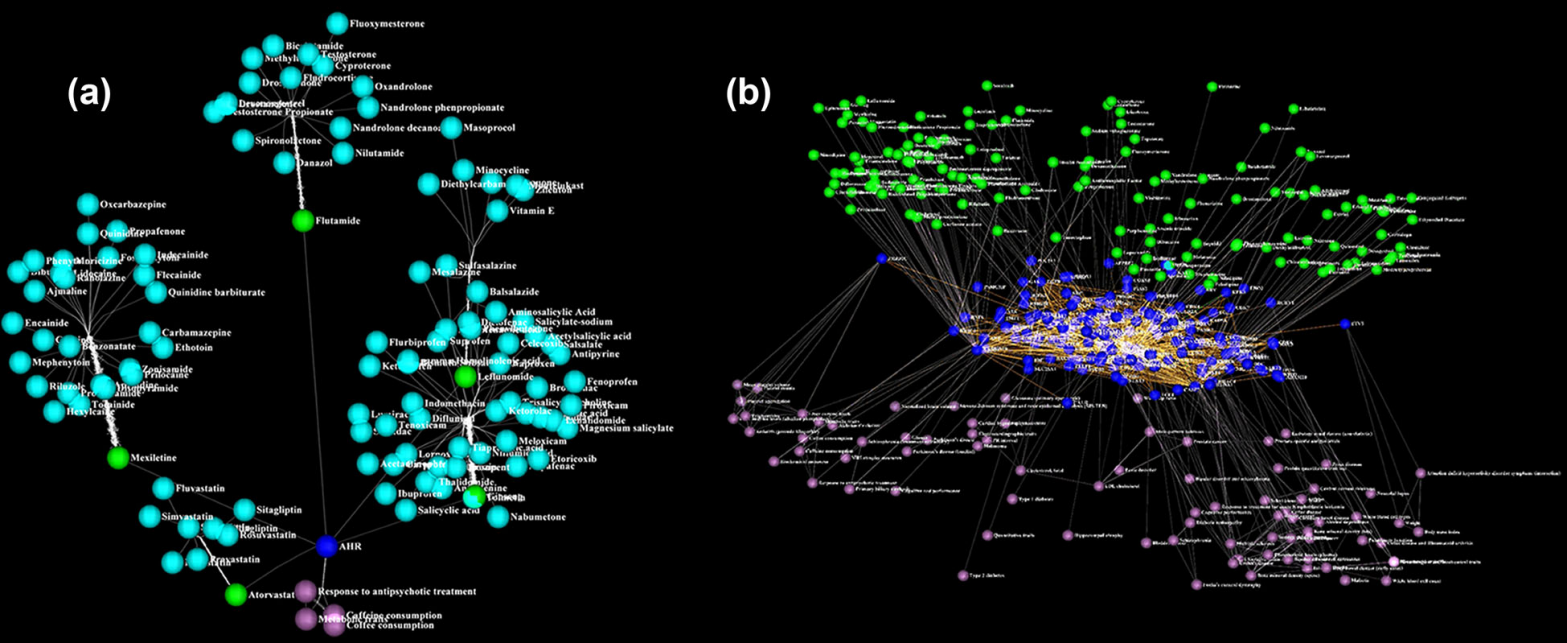
Visualizing multiple layers of information. (**a**) Using the iCAVE interface, we can pick a gene of interest (e.g., AHR, dark blue) and query the COMBO database for diseases that have been associated with AHR variants from GWAS studies (purple), drugs that are known to directly target AHR (green), and drug candidates that may directly interact with AHR (light blue). These drugs serve as an initial screening list of candidates for subsequent AHR binding site characterization. Semantics levels layout segregates the layers. **(b**) We can further query the COMBO database to generate a protein–protein interaction map of AHR (dark blue nodes, middle layer) and visualize the diseases associated with known single nucleotide polymorphisms in genes that code for AHR-interacting proteins (purple) and drugs that directly target them (green). We provide a more detailed movie of this 3-level semantics network with legible disease, gene, and drug names in Supplementary Video 3. For both panels, users can click on any edge or node for further information (e.g., exact disease variant location from GWAS studies).

Effective use of genomic information can depend on finding systems-level connections between multiple types of information, such as that between genomic variation, disease, and drugs [66–69]. Visualizing such data with semantic layout can assist in exploration in higher-level organization, all in 1 graph. The user can pick a gene (e.g., the Aryl Hydrocarbon Receptor (AHR) gene, dark blue) (Fig. 4A), query the COMBO database for diseases associated with its variants (purple), and identify drugs that target it (green) and drug candidates that may target it (light blue) due to guilt by association for having common targets with AHR-targeting drugs. These can serve as initial candidates for subsequent binding site characterization. Querying the COMBO database further generates a hierarchical network of proteins that interact with AHR (Fig. 4B, middle layer), diseases associated with gene variants of AHR-interacting proteins (purple), and AHR-targeting drugs (green).

### Graph clustering algorithms to identify network motifs

Clustering is critical in network exploration as biomolecules that cluster together tend be functionally related. iCAVE offers the following graph clustering algorithms:

“Edge-betweenness clustering” (EBC) is the number of shortest paths going through a particular edge. An edge with a high EB value connects multiple communities. At each step, the EBC algorithm removes the edge with the highest EB value until it has optimized a modularity metric on how unlikely the in-cluster degree of a node is in comparison to a random edge. EBC [70] is an attractive algorithm since it does not require an estimate of the number of clusters *a priori*, unlike a majority of existing graph clustering algorithms.

“Markov clustering” (MCL) [71] is a scalable and unsupervised algorithm that assumes that the number of intra-cluster connections is large and inter-cluster connections is small. It is based on a bootstrapping procedure that simulates random walks (flow) through the network, which expands or contracts in parallel with regional connectivity.

“Modularity clustering” (MC) uses the first eigenvector of the modularity matrix to assign nodes to clusters [72]. While ideal for weighted networks, MC delivers intuitive layouts for networks that do not have weights as well.

#### Cluster visualization layout algorithms

iCAVE can easily visualize clusters generated by iCAVE or another tool. By default, each cluster is positioned in space with “force-directed layout” [45], analogous to node positioning. Every cluster is embedded inside a transparent bubble, with members and their connections organized using the hemispherical layout. This arrangement provides a visual aesthetic, and (optional) edge bundling further clarifies the global topology (i.e., thicker bundles for high intra-cluster connectivity). Users can choose alternative layouts for cluster bubble positioning. “Lin-log cluster layout” is a variation of the force-directed model [45] where highly connected clusters are arranged in closer proximity.

**Figure 5:**
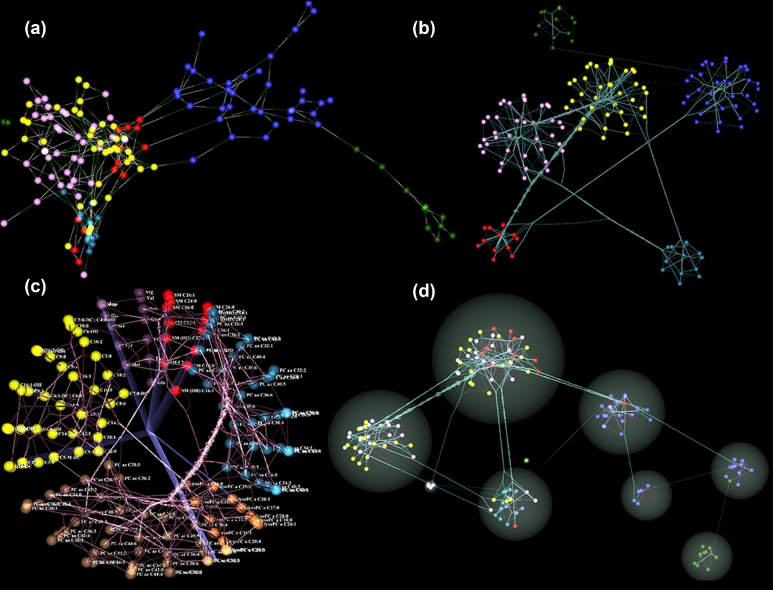
Pathway reconstructed high-throughput metabolomics data with Gaussian Graphical Modeling [88]; each sphere color represents a single metabolite class. (**a**) The force-directed layout of the weighted network captures the local cluster structures (snapshot). (**b**) Snapshot of user-defined metabolite clusters: cluster layout is force-directed while inside each cluster, and nodes are ordered in hemispherical layout. Edge bundling represents inter-cluster connectivity strength. (**c**) User-defined clusters of the same network in a Circos layout. We provide a movie to better investigate the network in Supplementary Video 4. (**d**) Markov Chain Clustering of the same network based on its connectivity, available as 1 of the clustering options in iCAVE. Each cluster is represented inside a spherical bubble. While topology suggests that most similar metabolites cluster together, this is not always the case, as shown. In all panels, the addition of metabolite labels is user-optional.

“Circos cluster layout” is an innovative algorithm we developed as a 3D adaptation of the popular 2D Circos layout [73]. In this algorithm, we arrange the nodes in 3D space as in hemispherical layout, where the most connected node is located at the center of the hemisphere. We then slice the hemisphere with (pie-like) panels that correspond to separate clusters. Cluster representations can be optimized by variations in node/slice colorings or edge bundling. Fig. 5 illustrates different cluster layout options using a metabolite network.

Within iCAVE's user interface, investigators can easily toggle between alternative layouts of a single graph to emphasize different network aspects. Users without stereo equipment can rotate, zoom, or scale the visual to investigate special structures, print 2D snapshots, and save movies of a rotating network. Rotation allows multiple views for users without 3D. Exporting and exchanging such movies is very convenient in the YouTube era, enabling easy publication and sharing with collaborators without iCAVE or stereo. Those with a stereo-enabled computer (or a CAVE facility) wear stereoscopic LCD shutter glasses that convey 3D image and allow immersive interaction. In a CAVE, sensors track the user's eye position and adjust perspective according to user movements. The mouse (or wand) gestures are mapped to logical events that the network layout application handles. Zoom and rotate options activated with a simple mouse (or wand) click help focus on a particular node or edge.

### Network topology statistics

Most real-world networks exhibit substantial and non-trivial features, where connections are neither purely regular nor random. iCAVE automatically generates and reports network topology statistics and centrality measures both graphically and in tabular form. These include the number of nodes, the number of edges, network diameter, node-betweenness centrality, closeness centrality, neighborhood connectivity, shortest path, topological coefficient, and node degree distribution properties of the network.

### Input/output

iCAVE input is provided as a tab-delimited text file of identifiers and optional information on magnitude of change, edge directionality, edge weights, node/edge colors, and patterns. Supplementary Table S2 includes the complete options list. Interaction data are read from an SQLite database. The user can modify the network in real time and store it in DB Browser (which is a light GUI editor for SQLite databases) as a .db file, so that it can be saved for later access. Output is the layout of the network drawn in the Virtual Reality User Interface (VRUI) environment, which can be saved as high-resolution 2D image snapshots (.png format) or movies (.gif format).

In iCAVE visualizations, 3D spherical glyphs represent nodes. Node color, size, and texture optionally encode further statistics (e.g., color for gene induction or repression, size for the magnitude of change in expression, texture to differentiate classes). Edges can be colored, patterned, or directed.

### Performance and scalability

Visualization and analysis of large networks in 3D may end up using a significant amount of computational resources, which in turn can affect the user experience. Size and topology of the network play an important role both in (i) rendering visual elements on the screen and (ii) calculating the results of a requested operation (e.g., layout, edge bundling, etc.). To help ease the computational burden of such factors while displaying networks in iCAVE, we implemented rendering and other compute-intensive operations to be adaptable to the size of the network. For example, as the network size increases, 3D objects are rendered in lower render quality. Similarly, for those compute-intensive operations, the tunable parameters (e.g., the number of iterations used for accuracy) are adjusted based on the network size.

## Discussion

iCAVE is a freely available open source biomolecular network visualization tool that leverages advanced 3D and immersive 3D display technologies and offers several display options integrated with an effective user interface. It incorporates a number of new and existing built-in network layout and graph clustering algorithms to enable automatic generation of 3D visualizations. Based on prior knowledge, input can additionally include (i) 3D node positions, (ii) cluster memberships, or (iii) multi-level hierarchies, or (iv) edge directionality. Utilizing iCAVE, investigators from diverse fields can gain insights from large, heterogeneous datasets and optimize the quality of their visualizations using different node color, size, and transparency options, as well as various edge weight, thickness, transparency, and directionality options. Network topological properties and centrality are also reported. While not extensive, the COMBO database enables disease researchers to conduct a quick query of their interactions among genes, drugs, and disease phenotypes.

We designed iCAVE with a modular software structure to create a general and flexible community resource. Users with intermediate programming experience can add algorithms for network layout, cluster layout, or graph clustering without affecting the core functionality of the code. They can also add datasets to COMBO. iCAVE is at the leading edge of immersive 3D network visualization. More user and case studies are needed to understand how best we can make use of immersion, stereoscopy, and 3D. Other layouts could possibly work well within 3D or immersive 3D, which we will explore further in future studies. We hope that iCAVE will encourage both programmers and biologists to enter the world of 3D human–computer interfaces, in response to the growing demands in exploring large complex data, and to facilitate further developments.

## Methods

### Input/output formats

iCAVE supports tabular input formats (.txt, .csv, or .tsv). Interactions are either user-defined, or are queries of the iCAVE COMBO database. Optional weights are represented with edge color frequency, directed edges with arrows, and node types with node glyph patterns. Input file options are listed in the User's Manual. Users can convert iCAVE input files to Cytoscape (Cytoscape, RRID:SCR_003032) or Gephi (Gephi, RRID:SCR_004293) input file formats or convert commonly used network input file formats (.sif, .csv) to those of iCAVE by utilizing scripts we provide in the iCAVE package. Networks are saved as static high-resolution (.png) images or movies of the rotating 3D image (.gif).

### Implementation

iCAVE uses VRUI, a development toolkit for interactive high-performance VR applications [47], which enables quick and scalable production of completely platform-independent software. iCAVE is thus portable between Linux and Mac system computers (optionally equipped with stereo capabilities) and CAVE facilities.

### Programming libraries

Several programming libraries provide intuitive and user-friendly rendering solutions. The VRUI library uses a C++ based OpenGL API platform that simplifies handling navigation transformations, light sources, menu creation, and rendering different objects. The SQLite3 software library handles large-scale database parsing. igraph library functions solve some of the programming challenges in generating regular and random graphs, manipulating graphs, and assigning attributes to nodes and edges. The ANSI C programming language library Argtable enables parsing user-defined 3D graphics options.

### Adding new algorithms

Node and edge data are stored in 2 separate “structure” arrays. “Node structure” stores its id, name, number of neighbors, color, texture, cluster, size, and coordinates. “Edge structure” includes start node id, end node id, weight, and color. Storage with structure arrays simplifies the addition of new layout algorithms because the arrays can be used as inputs. After layout coordinates are calculated, iCAVE utilizes OpenGL API for visualization. New algorithms are added as separate .cpp files, and the corresponding header files are imported to the main program (vrnetview.cpp).

### Label creation

Since VRUI offers limited label creation options that render low-quality and unreadable text, we developed texture mapping for high-quality rendering. Supplementary Fig. S1 illustrates VRUI versus iCAVE labels.

### User interface

Multiple functionalities demonstrate natural modes of interaction for effective analysis. These include activities such as selecting objects and interacting with the image in 3D space. While learning that a new user interface motif has been a traditional weakness of VR environments, more mature and practical technologies are becoming pervasive in consumer markets (e.g., motion sensors in Wii game consoles). These developments inform our user interface design and provide new users with familiar gestures and interaction motifs.

### Network exploration interface

Several features enable exploring, interacting, and modulating the networks in real time and saving the result. Interactive menu options are listed in Supplementary Table S2.

### User interface in CAVE environments

Investigators enter a CAVE environment wearing stereoscopic LCD shutter glasses that convey 3D image. When the user walks around, sensors track movements, and the video adjusts accordingly. Multiple users can exist simultaneously in the network and view the visualizations from multiple perspectives by moving in the space or directly interact with specific biomolecules by clicking on the handheld device to display all its interactions in that network or stored in the database. The user can alternatively investigate the network on his own computer.

### Output image generation

iCAVE assembles image snapshots from several viewpoints into one high resolution (.png) image (see Supplementary Fig. S2). The desired resolution is user-adjustable via a zoom factor.

### Network topology statistics

iCAVE automatically calculates the following network properties, rank-orders nodes based on these, and represents their distribution both graphically and in tabular form:

“Node degree property” yields hubs. Generally, only a few biomolecules (hubs) have many network interactions [74, 75]. Hubs are often central in mediating interactions among the less connected biomolecules [76, 77].

“Neighborhood connectivity metric” assists in identifying modularity, where small interconnected subgraphs may potentially represent specific enzymes, structures, or processes [78, 79] and provide significant insights into perturbed disease mechanisms. For example, the degree of gene co-expression correlates strongly with the complexity of an embedded motif [80].

“Network average and local clustering coefficients” quantify the connectivity of the whole network or a single node. The local clustering coefficient is the ratio between the numbers of edges that connect the neighbors of a node versus the maximum possible number of edges. The network average clustering coefficient is the average of the local clustering coefficients of all nodes [81]. Only nodes that belong to networks with >3 nodes are considered. The range of coefficient values varies from 0 (no interconnection) to 1 (perfect interconnection).

“Network closeness centrality and node closeness centrality” quantifies the velocity of information flow within a network (the reciprocal sum of the shortest paths from a selected node to all other nodes) [82]. Only nodes in subnetworks with >3 nodes are evaluated. When the shortest paths are calculated, each edge is scaled with corresponding weight, which can be a floating value. The average of all node closeness centrality values is the network closeness centrality value.

“Network diameter” is the length of shortest path between the 2 farthest nodes. Unconnected nodes are not considered. Irregular networks usually have small diameters, while regular networks have large diameters.

“Betweenness centrality” is a global metric on the importance of a node, which is equal to the number of shortest paths from all vertices to all others that pass through that node, calculating the “load” on a node [83]. Real-world, scale-free networks usually involve short path lengths across the network, and a few nodes have high betweenness centrality. “Connector” or “high-traffic” biomolecules that are vulnerable to targeted attacks usually suggest potential non-hub drug targets [84–86].

“Shared nearest neighbors” is a similarity metric based on the sharing of nearest neighbors between any 2 nodes, particularly useful in network topology-based motif, sub-graph, or cluster identification.

“Shortest paths” quantifies the importance of a node within the network, calculated by the number of shortest paths going through the node. Purely random graphs exhibit a small average shortest path length (∼the logarithm of the number of nodes) along with a small clustering coefficient.

### Layout algorithms

A graph *G(V = {1, …, n}, E)* represents a binary relation *E* over node set *V*. iCAVE both extends classical layouts to 3D and offers novel algorithms. Based on the underlying topology, a user can choose the best layout that helps with data interpretation. We provide below the details of algorithms we have implemented in iCAVE. Algorithms 1–5 are 3D extensions of 2D network layout algorithms that are based on the classical force-directed layout. In addition, we introduce two new layout algorithms, semantic layers and hemispherical, that we have developed to take advantage of the third dimension:

1. “Force-based layout.” The forces acting on each node in classical Fruchterman-Rheingold (FR) algorithm [45] are:
  \begin{equation*}
{f_a}(ij) = \frac{{d_{ij}^2}}{k}\quad
{f_r}(ij) = - \frac{{{k^2}}}{{{d_{ij}}}}\quad
k = \sqrt[3]{{\frac{{\rm volume}}{\hbox{number of nodes}}}},
\end{equation*}
  where *f_a_(ij)* and *f_r_(ij)* are attractive and repulsive forces, *d_ij_* is the distance between nodes *i* and *j*, and *k* is a constant corresponding to the equilibrium edge length.
2. “Lin-log layouts.” We used the r-PloyLog [53] energy model to implement the node-repulsion and edge-repulsion LinLog models. For all *r* ∈ *R* with *r > 0*, the node-repulsion energy of a layout *p* is
  \begin{eqnarray*}
\!\!\!\!\!{U_{r - NodePolyLog}}(p)
&=& \sum\limits_{\{ u,v\} \in E} \frac{1}{r}{{\| {p(u) - p(v)} \|}^r} \\
&& -\, \sum\limits_{\{ u,v\} \in \,{V^2}} {\ln \| {p(u) - p(v)} \|} ,
\end{eqnarray*}
  where *p(u)* is the position of node *u*. Edge-repulsion energy is
  \begin{eqnarray*}
{U_{r - EdgePolyLog}}(p)
&=& \sum\limits_{\{ u,v\} \in E} {\frac{1}{r}{{\| {p(u) - p(v)} \|}^r}}\nonumber\\
&& -,s \sum\limits_{\{ u,v\} \in \,{V^2}} {\deg (u)\deg(v){\rm ln} \| {p(u) - p(v)} \|} ,
\end{eqnarray*}
  where *deg(u)* is the number of edges incident to node *u*. At *r = 3*, the 3-PolyLog reduces to FR and at *r = 1* to the LinLog model. The LinLog models group nodes according to cut density and the normalized cut; therefore the layout leads to graph clustering.
3. “Hybrid force-directed layout” [52]. The original version of this algorithm is extremely computationally intensive, so we implemented a simplified version, reducing the run time at the expense of visualization quality. Our version has 3 steps: (i) position nodes randomly; (ii) partition the resulting graph; (iii) apply FR [45] algorithm separately on each subgraph. The partitioning step splits the graph into 2 sub-graphs (A and B) of equal sizes. This requires minimizing the cut size by calculating the second Eigenvector (Fiedler vector) λ of the following:
  $L( G )\ ^{^{\scriptscriptstyle\rightharpoonup}}\!\!\!\!{q} = \lambda ^{^{\scriptscriptstyle\rightharpoonup}}\!\!\!\!{q}$, where
  \begin{equation*}
\ ^{^{\scriptscriptstyle\rightharpoonup}}\!\!\!\!{q} =
\left( \begin{array}{@{}c@{}}
{{q_1}}\\
{{q_2}}\\
\vdots \\
{{q_n}}
\end{array} \right);\quad {q_i} =
\left\{
\begin{array}{c{@}{\quad}l}
1 & \forall i \in A\\
-1 & \forall i \in B
\end{array} \right.\quad and\ n \equiv \# \hbox{ of nodes}
\end{equation*}
  and *L(G)* is the Laplacian of graph *G*. The power-iteration algorithm solves for λ.
4. “Coarsened force-directed layout” [54] is suitable for large graphs as it combines FR with graph coarsening to speed up the calculations. In the first phase, the graph is coarsened until it reaches a minimum size (default = 3) or it does not coarsen more than a specific coarsening rate (default = 0.75). In the next phase, the layout of the coarsened graph is calculated using FR. Then, the layouts within the coarser graphs are recursively refined.
5. “Simulated annealing force-directed layout” [55] is ideal for large graphs with an aim to better distinguish clusters in the graph. It is originally based on FR with a fixed number of iterations. The algorithm follows a simulated annealing type schedule with liquid, expansion, cool down, crunch, and simmer phases. Long edges are cut based on a specified edge-cut value between 0 (no cut, resulting in standard FR) and 1 (aggressive cutting). The default edge-cut value used in iCAVE is 0.8, which allows clusters to separate from each other.
6. “Semantic levels layout” is ideal for integrative analysis of multiple data resources (e.g., genotype, phenotype, drugs, proteins, metabolites). Initially, the FR algorithm is performed in 2D. Then, multiple equidistant levels (default = 7) are created in the z-dimension. Based on network topology, we consecutively assign the nodes to one of the layers. The iCAVE user interface allows the manual manipulation of the number of layers and the distance between them. If layers are not predefined, we suggest experimenting with different options.
7. “Hemispherical layout.” We place *n* nodes of a graph *G(V = {1, …, n}, E)* equally spaced on a single 3D hemisphere surface, reducing the problem to finding a “hemispherical node ordering.” Coordinates for a node *i ε V* are *(x_*i*_, y_*i*_, z_*i*_) ε R*, at fixed hemisphere radius R:
  \begin{eqnarray*}
{x_i} &=& R*\cos ({\rm latitude}_i)*\cos ({\rm longitude}_i)\ 0^\circ \le {\rm longitude} \le 360^{\circ}; \\
{y_i} &=& R*\sin ({\rm longitude_i})*\cos ({\rm latitude}_i)\ 0^\circ \le {\rm latitude} \le 90^{\circ}; \\
{z_i} &=& R*\sin ({\rm latitude_i})\ 0 \le i \le \# \hbox{ of nodes}.\\
\end{eqnarray*}

Nodes are sorted and placed based on their degree, with the highest degree node at the hemisphere surface center. Algorithm inputs are the number of nodes, the graph center position, and hemisphere radius. Hemisphere radius, node sizes, colors, and textures are adjustable.

### Network clustering and bundling algorithms

The “edge-betweenness clustering algorithm” is an edge with a high EB value that potentially connects two or more “communities.” The edge with the highest EB value is removed at each step. The number of edges to be removed is user-defined (with a default of 0.2 times the number of edges). Any edge that leads to a single-node cluster is not removed.

The “edge bundling algorithm” is based on application of forces (electrostatic and spring) on an edge subdivided into multiple points. Edge compatibility metrics edge angle, scale (length), position, and visual compatibility are multiplied for total compatibility. If two edges are compatible above a threshold, forces are calculated and added to each subdivision, and those subdivisions are bundled together.

Project name: iCAVE

Project home page: http://research.mssm.edu/gumuslab/software.html

Download version of record: http://dx.doi.org/10.5524/100288

Operating systems: Unix, Linux, macOS

Programming language: C++

Other requirements: For macOS: XCode, X11/XQuartz, libjpeg, libz, libpng

License: GNU Lesser General Public License

## Supporting data and documents

Latest versions of the software, user manual, and tutorial are available for download at http://research.mssm.edu/gumuslab/software.html, released under the GNU Lesser General Public License. Snapshots of the software, input files, and videos used in this paper are also openly hosted in the *GigaScience* repository, GigaDB [87].

## Additional files

Supplementary Figure 1. Labels created with VRUI versus iCAVE. Left: VRUI intern method; Right: texture mapping.

Supplementary Figure 2. High-resolution image creation steps. Upper left image is a low-resolution snapshot created with common tools (800*600). This picture was projected into 2D screen plane (upper-right) and upper-left corner location was recorded as shown in upper right. 170 sub-images were taken to create a 18490*17560 resolution image (only 8 are shown in the bottom panels).

Supplementary Table 1. iCAVE currently provides a limited database within the COMBO repository for quick queries. Because of iCAVE's modular structure, users (who have some programming experience) can populate COMBO with additional databases.

Supplementary Table 2. iCAVE user-interactive menu options.

## Abbreviations

AHR: Aryl hydrocarbon receptor; CAVE: Cave automatic virtual environment; CBL: Cbl proto-oncogene; EBC: Edge-betweenness clustering; ENCODE: Encyclopedia of DNA elements; FR Fruchterman-Rheingold; GUI: Graphical user interface; GWAS: Genome wide association study; IBD: Inflammatory bowel disease; iCAVE: interactome-CAVE; LCD: Liquid crystal display; LeuT: Leucine transporter; MC: Modularity clustering; MCL: Markov clustering; LoF: Loss of function; miRNA: microRNA; ncRNA: non-coding RNA; OpenGL: Open graphics library; SNP: Single nucleotide polymorphism; SPRY2: Sprouty RTK signaling antagonist; TF: Transcription factor; VR: Virtual reality; 2D: Two dimensions; 3D: Three dimensions.

## Consent for publication

All authors consent for publication.

## Competing interests

None declared.

## Funding

This work was supported by the Concern Foundation Conquer Cancer Now Award (to Z.H.G), by computational resources provided by the Department of Scientific Computing at the Icahn School of Medicine at Mount Sinai, by PBTECH staff expertise of HRH Prince Alwaleed Bin Talal Bin Abdulaziz Alsaud Institute for Computational Biomedicine, and by the computational resources of the Coffrin Center for Biomedical Information at Weill Cornell Medical College of Cornell University (to V.L. and Z.H.G).

## Author contributions

Z.H.G. conceived and designed the study. V.L., S.K., and Z.H.G. contributed to data collection, analysis, and interpretation. All authors contributed to software development. V.L., S.K., and Z.H.G. contributed to drafting and critical revisions of the article. All authors approve the final version.

## Supplementary Material

GIGA-D-17-00026_Original-Submission.pdf

GIGA-D-17-00026_Revision-1.pdf

Response-to-Reviewer-Comments_Original-Submission.pdf

Reviewer-1-Report-(Original-Submission).pdf

Reviewer-1_Original-Submission-(Attachment).pdf

Reviewer-2-Report-(Original-Submission).pdf

Suppelment Materials

## References

[1] Newman MEJ Networks: An Introduction. New York, USA: Oxford University Press; 2006.

[2] Caldarelli G Scale-Free Networks: Complex Webs in Nature and Technology. New York, USA: Oxford University Press; 2007.

[3] Dorogovtsev SN, Mendes JFF, Samukhin AN Organization of modular networks. Phys Rev E 2008;78(5):056106. 10.1103/PhysRevE.78.056106 19113189

[4] Salwinski L, Eisenberg D Computational methods of analysis of protein–protein interactions. Curr Opin Struct Biol 2003;13(3):377–82. 12831890 10.1016/s0959-440x(03)00070-8

[5] Yook S, Oltvai ZN, Barabási A Functional and topological characterization of protein interaction networks. Proteomics 2004;4(4):928–42. 15048975 10.1002/pmic.200300636

[6] Taylor IW, Linding R, Warde-Farley D Dynamic modularity in protein interaction networks predicts breast cancer outcome. Nat Biotechnol 2009;27(2):199–204. 19182785 10.1038/nbt.1522

[7] Benson M, Breitling R Network theory to understand microarray studies of complex diseases. CMM 2006;6(6):695–701. 10.2174/156652406778195044 17022739

[8] Calvano SE, Xiao W, Richards DR A network-based analysis of systemic inflammation in humans. Nature 2005;437(7061):1032–7. 16136080 10.1038/nature03985

[9] Gao J, Aksoy BA, Dogrusoz U Integrative analysis of complex cancer genomics and clinical profiles using the cBioPortal. Sci Signal 2013;6(269):pl1. 23550210 10.1126/scisignal.2004088 PMC4160307

[10] Zhu J, Zhang B, Schadt EE A systems biology approach to drug discovery. Adv Genet 2008;60:603–35. 18358334 10.1016/S0065-2660(07)00421-X

[11] Brehme M, Hantschel O, Colinge J Charting the molecular network of the drug target Bcr-Abl. Proc Natl Acad Sci U S A 2009;10(186):7414–9. 10.1073/pnas.0900653106 PMC2670881 19380743

[12] Erler JT, Linding R Network medicine strikes a blow against breast cancer. Cell 2012;149(4):731–3. 22579276 10.1016/j.cell.2012.04.014

[13] Gorin MA, Pan Q Protein kinase C: an oncogene and emerging tumor biomarker. Mol Cancer 2009;8(1):9. 19228372 10.1186/1476-4598-8-9 PMC2647895

[14] Dudley JT, Butte AJ Identification of discriminating biomarkers for human disease using integrative network biology. Pac Symp Biocomput 2009;27–38. 19209693 PMC2749008

[15] Jin G, Zhou X, Wang H The knowledge-integrated network biomarkers discovery for major adverse cardiac events. J Proteome Res 2008;7(9):4013–21. 18665624 10.1021/pr8002886 PMC2854538

[16] Ou K, Yu K, Kesuma D Novel breast cancer biomarkers identified by integrative proteomic and gene expression mapping. J Proteome Res 2008;7(4):1518–28. 18318472 10.1021/pr700820g

[17] Gehlenborg N, O’donoghue SI, Baliga NS Visualization of omics data for systems biology. Nat Meth 2010;7(3s):S56–68. 10.1038/nmeth.1436 20195258

[18] Pavlopoulos GA, Malliarakis D, Papanikolaou N Visualizing genome and systems biology: technologies, tools, implementation techniques and trends, past, present and future. Gigascience 2015;4(1):38. 26309733 10.1186/s13742-015-0077-2 PMC4548842

[19] http://pathguide.org (1 January 2015, date last accessed).

[20] Bader GD Pathguide: a pathway resource list. Nucleic Acids Res 2006;34(90001):D504–6. 16381921 10.1093/nar/gkj126 PMC1347488

[21] Shannon P, Markiel A, Ozier O Cytoscape: a software environment for integrated models of biomolecular interaction networks. Genome Res 2003;13(11):2498–504. 14597658 10.1101/gr.1239303 PMC403769

[22] Bastian M, Heymann S, Jacomy M Gephi: an open source software for exploring and manipulating networks. In: International AAAI Conference on Weblogs and Social Media, San Jose, CA, 2009 http://www.aaai.org/ocs/index.php/ICWSM/09/paper/view/154 (1 January 2014, date last accessed).

[23] Jacomy A, Plique G http://sigmajs.org/ (1 January 2014, date last accessed).

[24] http://igraph.org (1 January 2014, date last accessed).

[25] Yeung N, Cline MS, Kuchinsky A Exploring biological networks with Cytoscape software. Curr Protoc Bioinformatics 2008;chapter 8:unit 8.13. 10.1002/0471250953.bi0813s23 18819078

[26] www.ingenuity.com (1 January 2014, date last accessed).

[27] Breitkreutz B, Stark C, Tyers M Osprey: a network visualization system. Genome Biol 2003;4(3):R22. 12620107 10.1186/gb-2003-4-3-r22 PMC153462

[28] Hu Z, Mellor J, Wu J VisANT: data-integrating visual framework for biological networks and modules. Nucleic Acids Res 2005;33(web server):W352–7. 15980487 10.1093/nar/gki431 PMC1160192

[29] Gerasch A, Faber D, Küntzer J BiNA: a visual analytics tool for biological network data. PLoS One 2014;9(2):e87397. 24551056 10.1371/journal.pone.0087397 PMC3923765

[30] Schadt EE, Linderman MD, Sorenson J Computational solutions to large-scale data management and analysis. Nat Rev Genet 2010;11(9):647–57. 20717155 10.1038/nrg2857 PMC3124937

[31] Greffard N, Picarougne F, Kuntz P Beyond the classical monoscopic 3D in graph analytics: an experimental study of the impact of stereoscopy. In: 2014 IEEE VIS International Workshop on 3DVis, Phoenix, AZ, 2014 p. 19–24. http://ieeexplore.ieee.org/document/7160095/ (1 January 2014, date last accessed).

[32] Ware C, Mitchell P Visualizing graphs in three dimensions. ACM Trans Appl Percept 2008;5(1):1–15.

[33] Sollenberger RL, Milgram P Effects of stereoscopic and rotational displays in a three-dimensional path-tracing task. Hum Factors 1993;35:483–99. 8244411 10.1177/001872089303500306

[34] Kwon O, Muelder C, Lee K A study of layout, rendering, and interaction methods for immersive graph visualization. IEEE Trans Visual Comput Graphics 2016;(7):1802–15. 10.1109/TVCG.2016.2520921 26812726

[35] Bhavnani SK, Ganesan A, Hall T Discovering hidden relationships between renal diseases and regulated genes through 3D network visualizations. BMC Res Notes 2010;3(1):296. 21070623 10.1186/1756-0500-3-296 PMC3001742

[36] Ruths DA, Chen ES, Ellis L Arbor 3D: an interactive environment for examining phylogenetic and taxonomic trees in multiple dimensions. Bioinformatics 2000;16(11):1003–9. 11159311 10.1093/bioinformatics/16.11.1003

[37] Quon G, Gordon P, Sensen C 4D bioinformatics: a new look at the ribosome as an example. IUBMB Life 2003;55(4–5):279–83. 12880210 10.1080/1521654031000136255

[38] Turinsky AL, Fanea E, Trinh Q CAVEman: standardized anatomical context for biomedical data mapping. Anat Sci Ed 2008;1(1):10–18. 10.1002/ase.3 19177373

[39] Yang Y, Engin L, Wurtele ES Integration of metabolic networks and gene expression in virtual reality. Bioinformatics 2005;21(18):3645–50. 16020466 10.1093/bioinformatics/bti581

[40] http://nvidia.com/get3D (1 January 2014, date last accessed).

[41] Pavlopoulos GA, O’donoghue SI, Satagopam VP Arena3D: visualization of biological networks in 3D. BMC Syst Biol 2008;2(1):104. 19040715 10.1186/1752-0509-2-104 PMC2637860

[42] Wang Q, Tang B, Song L 3DScapeCS: application of three dimensional, parallel, dynamic network visualization in Cytoscape. BMC Bioinformatics 2013;14(1):322. 24225050 10.1186/1471-2105-14-322 PMC3835703

[43] Freeman TC, Goldovsky L, Brosch M Construction, visualisation, and clustering of transcription networks from microarray expression data. PLoS Comput Biol 2007;3(10):2032–42. 17967053 10.1371/journal.pcbi.0030206 PMC2041979

[44] Cerami E, Demir E, Schultz N Automated network analysis identifies core pathways in glioblastoma. PLoS One 2010;5(2):e8918. 20169195 10.1371/journal.pone.0008918 PMC2820542

[45] Fruchterman TMJ, Reingold EM Graph drawing by force-directed placement. Softw Pract Exper 1991;21(11):1129–64.

[46] Ellis SR, Tharp GK, Grunwald AJ Exocentric judgements in real environments and stereoscopic displays. In: Proceedings of the Human Factors and Ergonomics Society Annual Meeting, San Francisco, CA, 1991. Vol 35, p. 1442–6. Sage, CA: SAGE Publications; 1991.

[47] Etemadpour R, Monson E, Linsen L The effect of stereoscopic immersive environments on projection-based multi-dimensional data visualization. In: Information Visualisation (IV), 2013 17th International Conference, London, UK, 2013 p. 389–97.

[48] Stephen G, Eick SGE Aspects of network visualization Computer Graphics and Applications 1996;16(2):69–72.

[49] Wagemans J, Elder JH, Kubovy M A century of Gestalt psychology in visual perception: I. Perceptual grouping and figure–ground organization. Psychol Bull 2012;138(6):1172–217. 22845751 10.1037/a0029333 PMC3482144

[50] Holten D, Van Wijk JJ Force-directed edge bundling for graph visualization. Comput Graph Forum Proc EuroVis 2009;28(3):983–90.

[51] Kamada T, Kawai S An algorithm for drawing general undirected graphs. Inform Process Lett 1989;31(1):7–15.

[52] Frishman Y, Tal A Multi-level graph layout on the GPU. IEEE Trans Visual Comput Graphics 2007;13(6):1310–9. 10.1109/TVCG.2007.70580 17968079

[53] Noack A Energy models for graph clustering. JGAA 2007;11(2):453–80.

[54] Hu Y Efficient and high quality force-directed graph drawing. Math J 2005;10:37–71.

[55] Martin S, Brown W, Klavans R OpenOrd: an open-source toolbox for large graph layout. InIS&T/SPIE Electron Imaging 2011;786806.

[56] Gandy S, Haroutunian V, Dekosky ST CR1 and the “vanishing amyloid” hypothesis of Alzheimer's disease. Biol Psychiatry 2013;73(5):393–5. 23399469 10.1016/j.biopsych.2013.01.013 PMC3600375

[57] https://www.encodeproject.org/ (1 January 2014, date last accessed).

[58] Gerstein MB, Kundaje A, Hariharan M Architecture of the human regulatory network derived from ENCODE data. Nature 2012;489(7414):91–100. 22955619 10.1038/nature11245 PMC4154057

[59] Khurana E, Fu Y, Chen J Interpretation of genomic variants using a unified biological network approach. PLoS Comput Biol 2013;9(3):e1002886. 23505346 10.1371/journal.pcbi.1002886 PMC3591262

[60] Khurana E, Fu Y, Colonna V Integrative annotation of variants from 1092 humans: Application to cancer genomics. Science 2013;342(6154):1235587. 24092746 10.1126/science.1235587 PMC3947637

[61] http://www.hprd.org (1 January 2014, date last accessed).

[62] http://www.ebi.ac.uk/intact (1 January 2014, date last accessed).

[63] http://www.genome.gov/gwastudies (1 January 2014, date last accessed).

[64] http://stich.embl.de (1 January 2014, date last accessed).

[65] http://www.drugbank.ca (1 January 2014, date last accessed).

[66] Butcher EC, Berg EL, Kunkel EJ Systems biology in drug discovery. Nat Biotechnol 2004;22(10):1253–9. 15470465 10.1038/nbt1017

[67] Chanda SK, Caldwell JS Fulfilling the promise: drug discovery in the post-genomic era. Drug Discov Today 2003;8(4):168–74. 12581711 10.1016/s1359-6446(02)02595-3

[68] Searls DB Pharmacophylogenomics: genes, evolution and drug targets. Nat Rev Drug Discov 2003;2(8):613–23. 12904811 10.1038/nrd1152

[69] Yildirim MA, Goh K, Cusick ME Drug target network. Nat Biotechnol 2007;25(10):1119–26. 17921997 10.1038/nbt1338

[70] Girvan M, Newman MEJ Community structure in social and biological networks. Proc Natl Acad Sci U S A 2002;99(12):7821–6. 12060727 10.1073/pnas.122653799 PMC122977

[71] van Dongen S Graph Clustering by Flow Simulation. Utrecht, The Netherlands: University of Utrecht; 2000.

[72] Newman MEJ Modularity and community structure in networks. Proc Natl Acad Sci U S A 2006;103(23):8577–82. 16723398 10.1073/pnas.0601602103 PMC1482622

[73] Krzywinski M, Schein J, Birol I Circos: an information aesthetic for comparative genomics. Genome Res 2009;19(9):1639–45. 19541911 10.1101/gr.092759.109 PMC2752132

[74] Barabási AL, Albert R Emergence of scaling in random networks. Science 1999;286(5439):509–12. 10521342 10.1126/science.286.5439.509

[75] Albert R Scale-free networks in cell biology. J Cell Sci 2005;118(21):4947–57. 16254242 10.1242/jcs.02714

[76] Jeong H, Mason SP, Barabási A-L Lethality and centrality in protein networks. Nature 2001;411(6833):41–42. 11333967 10.1038/35075138

[77] Jonsson PF, Bates PA Global topological features of cancer proteins in the human interactome. Bioinformatics 2006;22(18):2291–7. 16844706 10.1093/bioinformatics/btl390 PMC1865486

[78] Milo R Network motifs: simple building blocks of complex networks. Science 2002;298(5594):824–7. 12399590 10.1126/science.298.5594.824

[79] Shen-Orr SS, Milo R, Mangan S Network motifs in the transcriptional regulation network of *Escherichia coli*. Nat Genet 2002;31(1):64–68. 11967538 10.1038/ng881

[80] Bhardwaj N, Lu H Co-expression among constituents of a motif in the protein-protein interaction network. J Bioinform Comput Biol 2009;7(01):1–17. 19226657 10.1142/s0219720009003959 PMC2770376

[81] Watts DJ, Strogatz SH Collective dynamics of “small-world” networks. Nature 1998;393(6684):440–2. 9623998 10.1038/30918

[82] Newman MEJ A measure of betweenness centrality based on random walks. Social Networks 2005;27 (1):39–54.

[83] Brandes U A faster algorithm for betweenness centrality*. J Math Sociol 2001;25:163–77.

[84] Csermely P, Agoston V, Pongor S The efficiency of multi-target drugs: the network approach might help drug design. Trends Pharmacol Sci 2005;26(4):178–82. 15808341 10.1016/j.tips.2005.02.007

[85] Joy MP, Brock A, Ingber DE High-betweenness proteins in the yeast protein interaction network. J Biomed Biotechnol 2005;2(2005):96–103. 10.1155/JBB.2005.96 PMC1184047 16046814

[86] Yu H, Kim PM, Sprecher E The importance of bottlenecks in protein networks: correlation with gene essentiality and expression dynamics. PLoS Comput Biol 2007;3(4):e59. 17447836 10.1371/journal.pcbi.0030059 PMC1853125

[87] Liluashvili V, Kalayci S, Fluder E Supporting data for “iCAVE: an open source tool for visualizing biomolecular networks in 3D, stereoscopic 3D and immersive 3D.” GigaScience Database 2017 http://dx.doi.org/10.5524/100288. 10.1093/gigascience/gix054 PMC5554349 28814063

[88] Krumsiek J, Suhre K, Illig T Gaussian graphical modeling reconstructs pathway reactions from high-throughput metabolomics data. BMC Syst Biol 2011;5:21. 21281499 10.1186/1752-0509-5-21 PMC3224437

