# Supplementary material for: iCAVE: an open source tool for visualizing biomolecular networks in 3D, stereoscopic 3D and immersive 3D
Source: Gigascience. 2017 Jul 15;6(8):1–13. doi: 10.1093/gigascience/gix054 (PMC5554349; doi:10.1093/gigascience/gix054)
Supplement: GIGA-D-17-00026_Original-Submission.pdf [file gix054_GIGA-D-17-00026_Original-Submission.pdf]

Manuscript
Click here to download Manuscript Gumus_iCAVE_Gigascience_2017.pdf

iCAVE: an open source tool for visualizing biomolecular networks in 3D, stereoscopic 3D and immersive 3D

Vaja Liluashvili[1,2], Selim Kalaycı[1,2], Eugene Fluder[1,2], Manda Wilson[3], Aaron Gabow[3] & Zeynep H. Gümüş[1,2,*]

[1]Department of Genetics and Genomic Sciences, [2]Icahn Institute for Genomics and Multiscale Biology, Icahn School of Medicine at Mount Sinai, New York, NY 10029

[3]Computational Biology Center, Memorial-Sloan Kettering Cancer Center, New York, NY 10065

*To whom correspondence should be addressed: zeynep.gumus@gmail.com

Abstract

Background. Visualizations of biomolecular networks assist in systems-level data exploration in many cellular processes. Data generated from high-throughput experiments increasingly inform these networks, yet current tools do not adequately scale with concomitant increase in their size and complexity.

Findings. We present an open-source software platform, interactome-CAVE (iCAVE), for visualizing large and complex biomolecular interaction networks in three dimensions (3D). Users can explore networks (i) in 3D using a desktop; (ii) in stereoscopic 3D using 3D-vision glasses and a desktop; or (iii) in immersive 3D within a CAVE environment. iCAVE introduces 3D extensions of known 2D network layout, clustering, and edge-bundling algorithms, as well as new 3D network layout algorithms. Furthermore, users can simultaneously query several built-in databases within iCAVE for network generation, or visualize their own networks (e.g. disease, drug, protein, metabolite). iCAVE has modular structure that allows rapid development by addition of algorithms, datasets or features without affecting other parts of the code.

Conclusions. Overall, iCAVE is the first freely available open source tool that enables 3D (optionally stereoscopic or immersive) visualizations of complex, dense or multi-layered biomolecular networks. While primarily designed for researchers utilizing biomolecular networks, iCAVE can assist researchers in any field.

Keywords. Biomolecular networks; network visualization; visualization; immersive; stereoscopic; CAVE.

Background

Interaction networks are one of the primary visual metaphors for communicating and understanding –omics data at a systems level. From cellular organisms to human society, networks provide critical clues on systems-level behavior [1–3]. In biomedicine, they are essential for understanding normal [4,5] and disease states [6–9], and instrumental for drug discovery [10–12] as well as biomarker identification [13–15]. Changes in networks have helped in prognosis for breast cancer patients [6], analyzing systematic inflammation in humans [8], or studying emerging tumor markers [16]. Network visualizations are thus important in basic and translational biomedical research, with an abundance of tools for their exploration [17,18]. Many tools are also coupled with public databases, enabling visualizations in the context of previous knowledge [17]. In fact, currently more than five hundred resources are listed at http://pathguide.org with thousands of networks, and millions of biomolecular interactions [19].

Among currently available tools, Cytoscape [20] is quite popular. There are also a number of JavaScript network visualization libraries (e.g. sigma.js http://sigmajs.org/), and software packages (e.g. iGraph http://igraph.org) on the web. However, the layout algorithms in these libraries and employed in Cytoscape [21], in addition to other tools like Ingenuity [22], Osprey [23], VisANT [24], BINA [25] to name a few, are limited by the number of molecules and interactions that can be displayed on a 2D-screen, and the associated layout and representation challenges. Furthermore, recent technological developments have increased the size and complexity of –omics experimental data, with simultaneous recordings from multiple cellular events, leading to unprecedented growth in interaction data [26]. New approaches are necessary to address the visualization design challenges the concomitant large, complex and multi-dimensional (multi-layered) networks present to explore these systems.

Currently networks with 3D localizations (e.g. brain connectivity networks or molecular complexes) are best explored in 3D. For representing abstract data, taking advantage of the third dimension can also allow for greater freedom, however, the available 3D visualization options and tools are still somewhat nascent in this domain. Stereoscopy, the projection of separate images to each eye, which creates the illusion that virtual objects have volumes in 3D-space has been shown to be particularly beneficial for exploring large, complex net-

works, either alone or combined with rotation or user motion cues [27–29]. Immersive visualization environments where users are virtually immersed inside the image, have also led to better performance than 2D in user studies for relatively difficult tasks and large networks [30]. For example, a CAVE environment, which includes projectors directed to several walls of a room-sized cube to create the sense of presence inside a virtual world, has been shown to help identify a new network property that a 2D-display failed [31]. The technology features that were particularly helpful were stereoscopy, magnification and wide field-of-view (the extent of the visualized image observed by the user) [31]. However, a CAVE facility is a substantial investment to build and maintain that only a limited number of institutions have. Network visualizations in stereoscopic and immersive-3D environments are still new, and we currently do not have community tools to help us understand how best to use them by conducting user studies on different technology platforms, and testing alternative layout algorithms or to explore phenomena that involve large, complex networks.

Here, we introduce interactome-CAVE (iCAVE), an open-source tool for 3D, stereoscopic 3D and immersive 3D visualizations of complex, large, and/or multi-layered networks. iCAVE development is made possible by the continuous evolution of data analysis tools in VR, stereoscopic visualization and emerging 3D technologies. Use of VR technology in life sciences research is still nascent [32–35], and so far does not include free open source tools for biomolecular network visualizations, mainly due to the limited portability of the technology to personal computers until recently. iCAVE is completely portable, taking advantage of recent advances in computer graphics hardware, software and content creation that are leading to a proliferation of stereoscopic visualization capabilities in personal computing. Computers can now be upgraded to display high quality stereoscopic 3D visuals with low-cost stereoscopic 3D-glasses and software [36], which are much cheaper than recent Head Mounted Displays (HMDs). As most scientific computers are becoming stereo-enabled and 3D glasses are going mainstream, iCAVE is on the leading edge of this larger trend in the evolution of visual computing technology. If a computer is equipped with stereo capabilities, users can explore stereoscopic-3D visualizations. With large screens or curved walls, users can additionally take advantage of magnification and wide field-of-view. Users can also use iCAVE in immersive CAVE environments. Without a CAVE or a stereo-equipped computer (or if users choose to turn off stereo), iCAVE provides interactive 3D visualizations that still offer most of its features.

Note that while few network visualization tools incorporate 3D layouts [37–39], they are not immersive 3D, i.e. they do not have interoperation capability with Virtual Reality (VR) technologies, and have 2D displays. For example, Arena 3D [37] mixes 3D and 2D properties by arranging data in multilayered graphs in 2D, with each layer representing a different data type. While the tool includes several layout and clustering algorithms for each layer, and has zoom and rotation features, it does not offer global layout and clustering algorithms to make full use of the third dimension and each layer is in 2D [37]. 3DScapeCS [38] is a Cytoscape PlugIn written in Java, with built-in extensions of the classic 2D force-directed layouts. Users cannot add new layouts or functionalities and it does not utilize 3D-effects to improve comprehension (e.g. transparency or advanced shadow effects). BioLayoutExpress [39] (now Miru) is a stand-alone 3D application specifically for gene expression networks that offers three network layouts, a clustering method, no edge bundling and with limited network topology statistics. Importantly, it is not freely available. In summary, 3D biomolecular network visualization is a nascent field. We need free open-source tools for biologists to visualize their networks, and for algorithm developers to add and test new methods that take advantage of the third dimension. Such a tool will also enable visualization designers to perform user studies to better understand the relative advantages of various 3D features. This is necessary, as how best to utilize features specific to 3D or to take advantage of new 3D technologies are currently open research questions.

To the best of our knowledge, iCAVE is the first 3D, stereoscopic-3D and immersive-3D biomolecular network visualization tool that is open source, freely available and utilizable with commercial hardware/software. iCAVE introduces new built-in 3D algorithms for laying out nodes and their connections in 3D space and has built-in topology-based graph clustering algorithms For example, it enables visual integration of multiple clusters or data types within the same graph as a multi-layered network (e.g. metabolomic, proteomic, genomic, GWAS-disease, protein-drug interactions). Users can also add their own layout or clustering algorithms. While not extensive, it includes a few built-in databases to assist in preliminary mapping of High-Throughput (HT) experimental data in early discovery phase of network building. Customizable color, texture, size and layout options assist in displaying maximum information in a graph in an optimized manner. Users can easily select edge colors, weights and directions or bundle edges for simplified views. Data are input in a tab-delimited text file while visual outputs can be saved in 2D-snapshots or movies configured with user-defined rotation, zoom and

speeds. Additional reports on network statistics are provided in 2D. Overall, iCAVE enables network explorations in hypothesis-driven contexts that is flexible, collaborative and user friendly.

Results

Optional Stereoscopy. iCAVE users can turn 3D stereoscopy on or off during exploration. For example, consider rendering the 2D biomolecular network in Fig. 1A that represents a pathway affected by genomic alterations in glioblastoma[40]. Instead of the static 2D network in Fig. 1A, users can experience full 3D depth perception at the comfort of their own stereo-equipped computer (Fig. 1B), or inside a CAVE (Fig. 1D) using a simple 3D extension of a classical force-directed layout algorithm [41] (Fig 1). Even users without a stereo-equipped computer can interact with the 3D network: they can use their mouse (in lieu of hand-held controls) to zoom in/out or rotate the network to a view without occlusions. Rotation and zoom enables viewing the network from different view angles, such as the screenshot in Fig.1C. User studies have shown that even simple 3D features like rotation help better identification of properties unique to complex networks [29]. Visualizations using stereoscopic 3D or immersive environments that enable inspection of a system from multiple perspectives have also been shown to make different properties of a system clearer [42]. Our case study supports this, as we observed a network feature that was not intuitive from the original 2D layout in Fig. 1A: nodes CBL and SPRY2 (with *) are *connectors* between two dense network regions (modules) (Fig. 1A-C). A targeted attack to these genes can split the network into two. We could not identify this in 2D. Such discoveries of network topological features, among others, give a richer, more intuitive and ultimately more insightful understanding of networks.

Addressing Large Networks. Important characteristics may be missed if users cannot interact with the complete network. In the simplest case, the nodes may form (i) dense sub-networks that are interconnected by a small number of *connector* nodes which render them critical or (ii) multiple networks (often one giant and few smaller ones) where the smaller sub-networks may represent functional groups of importance, such as a critical enzyme complexes. Hence, visualizing the complete network can be advantageous even if it is very large, to identify local patterns [43]. However, while human brain has a remarkable capacity to visually identify patterns, enabling interpretation of data, visualizations of large networks may exhibit problems with display clutter,

molecular positioning or perceptual tension, leading the user to misinterpret closely positioned molecules as related [44]. Such misinterpretations are inherent in the limitations of human visual perception, and have been well-studied in (Gestalt) psychology: people tend to organize visual elements into groups [45].

In 3D elements that appear to form a pattern because of their visual positioning in one viewpoint can be interpreted correctly by rotating the image to a different viewpoint (e.g. Fig. 1). Furthermore, in networks that are denser or larger than that of Fig. 1, the potential 2D *hairball* effect can obscure important interactions. iCAVE users can simply navigate to a view without occlusions by moving their head, rotating the image, and zooming in or out, eliminating *edge-crossings*. To further address cluttering, iCAVE provides an *edge-bundled display* [46] option for visually bundling adjacent edges together, analogous to bundling electrical wires or cables. Bundling is extremely useful in identifying global patterns in very large networks and can suggest vulnerabilities as targets. Several layout algorithms built-in within iCAVE address the molecular positioning problem; depending on the topology of a network, one may work better than another. We suggest testing each to see which works best. We provide examples of how these features can help with exploring a network in the following sections.

New biological insights from networks with known 3D physical coordinates. Users can visualize physically constrained networks at multiple scales, from proteins (Fig. 2A) to the whole brain (Fig. 2B). Coupled with edge bundling, these can provide insights in hypothesis generation. For example, Fig. 2A represents a snapshot of bacterial leucine transporter (LeuT) residue correlation network, where tnodes represent 3D coordinates of alpha-carbon of a residue and edges represent top 3,000 (Pearson) correlations between residue pairs from a Molecular Dynamics simulation (from Michael LeVine, personal communication). Remarkably, bundling the edges of this network enables the representation of highest density *correlation highways* that travel through substrate permeation core in protein center, connecting extracellular and intracellular domains. These highways enable users to identify specific residues that have dense correlations with the permeation core even if they are away from it, which is unexpected. These residues may have previously unidentified importance in protein structure and function and are therefore potential candidates for follow-up studies.

Automated Layouts utilize 3D for molecular positioning. Biomolecular networks tend to follow basic and reproducible organizing principles, and navigating the entire network provides a good initial understanding. The layout algorithm must address the complex problem of arranging the nodes to clearly disseminate the network topology, and at the same time be visually pleasant and user-friendly. iCAVE offers several network layout options to achieve these aims:

Due to user familiarity, we extended variations of the force-directed layout to 3D: (i) the classical *force-directed algorithm* [41] treats the network as a physical system with edges analogous to *springs* and nodes to *electrically charged particles* that repel each other. Final layout is established when the repulsive and attractive forces balance each other [47]; (ii) *Lin-log layout* [48] is better suited for larger networks because it keeps highly connected nodes in close proximity with minimal number of edge crossings; and (iii) *hybrid force-directed layout* [49] partitions the graph into smaller units before applying the force-directed algorithm (see Methods). We further implemented two novel layout algorithms to take full advantage of immersive 3D:

Semantic levels layout algorithm segregates the network into separate layers (default 7) in the third dimension. The layout of each layer is calculated with a 3D extension of the force-directed approach. Semantic layers layout can be especially useful for user-defined networks where the number of layers and node assignments to layers can correspond to different data types (e.g. a 2D projection in Fig. 4 and 3D video in Supplementary Video 3, with layer1: genes; layer2: diseases; layer3: drugs).

Hemispherical layout is a novel layout algorithm we have developed, that positions the network on the surface of a 3D hemisphere. The most connected node is positioned at the top center of the hemisphere. Then, the whole hemisphere surface is populated based on a decreasing rank-order of connectivity. The node positions are fixed and the edges are drawn on the hemisphere surface (e.g. see a 2D projection in Fig. 5C and 3D video in Supplementary Video 4).

Each layout algorithm has unique strengths and we recommend the user to test different options. Semantic layout is often ideal for hierarchical networks. Force-directed layout often captures the essence of large net-

works. Hemispherical layout leads to clean images with optional edge bundling (Fig. 5C and Supplementary Video 4).

Statistics on Network Topological Properties. Most real-world networks exhibit substantial and non-trivial features, where connections are neither purely regular nor random. iCAVE automatically generates and reports network topology statistics and centrality measures both graphically and in tabular form. These include the number of nodes, the number of edges, network diameter, node-betweenness centrality, closeness centrality, neighborhood connectivity, shortest path, topological coefficient, and node degree distribution properties of the network.

COMBO Database for Simultaneous Query of Multiple Data Types. Publicly available biomolecular interaction data are often contained in massive databases [19]. While not comprehensive, iCAVE combines data from multiple resources into a single COMBO repository to enable quick queries. These include protein-protein interaction databases Human Protein Reference Database (http://www.hprd.org) and intact (http://www.ebi.ac.uk/intact), disease and associated gene variants database (http://www.genome.gov/gwastudies); and drug-target databases STITCH (http://stich.embl.de) and DRUGBANK (http://www.drugbank.ca). Pathways database SuperPathway is stored separately (personal communication with Josh Stuart, UCSD). Users can add their own databases without affecting other parts of the code. Details on COMBO database are given in Supplementary Table 1.

Visualizing Multiple Layers of Information. Effective use of genomic information can depend on finding systems-level connections between multiple types of information, such as that of between genomic variation, disease and drugs [50–53]. Visualizing such data with semantic layout can assist in exploration in higher-level organization, all in one graph. User can pick a gene (e.g. AHR, dark blue, Fig. 4A), query the COMBO database for diseases associated with its variants (purple); identify drugs that target it (green) and drug candidates that may target (light blue) due to guilt by association for having common targets with AHR-targeting drugs.

These can serve as initial candidates for subsequent binding site characterization. Querying COMBO database further generates a hierarchical network of proteins that interact with AHR (Fig. 4B, middle layer), diseases associated with gene variants of AHR-interacting proteins (purple) and AHR targeting drugs (green).

Illustrative Examples.

Example 1. Visualizing the complete global network, even if it is very large, can enable visual identification of a pattern. For example, consider a large probabilistic causal network constructed from human omental adipose tissue in a morbidly obese patient cohort in Fig. 3A. The network consists of 7,601 nodes, 13,979 edges[54]. Nodes are the genes expressed in tissue; edges are derived from a Bayesian network reconstruction algorithm that leverages DNA variation for causality. Here, we highlight nodes that represent a signature of genes causally associated with inflammatory bowel disease (IBD) SNPs or disease pathways. Notice that within this global view of the massive network, there is a pattern of the IBD genes clustering together, which visually supports the hypothesis of functional relatedness.

Example 2. While force-directed layout algorithms can help identify global patterns, if the interaction network has a hierarchy, semantic layers layout can help visualize the hierarchical nature of the interactions easily. For example, Fig. 3B displays the global view of network generated from The Encyclopedia of DNA Elements (ENCODE https://www.encodeproject.org/) study data. The ENCODE Consortium is generating a comprehensive parts list of the human genome functional elements, including those that control active genes, such as transcription factors (TFs). Utilizing these unprecedented volumes of data, Gerstein and co-workers have generated the massive network in Fig. 3B that includes 119 TFs that target 9,057 genes (nodes) via 26,037 interactions (edges)[55] . Using force-directed layouts, users can capture the general network structure and differentiate a TF from its neighbors by zooming in/out, adding labels to that specific TF, etc, as well as obtain statistics on its network centrality and other global topological properties as they pertain to the network. However, the semantic layers layout is useful in visualizing the hierarchical nature of this network, integrating TF, non-coding RNA (ncRNA), miRNA and protein-protein interaction data (Fig. 3D and Supplementary Video 2). Here, network connectivity and hierarchy reflects genomic properties:top level TF-binding correlates with target ex-

pression, mid-level contains 'information flow bottlenecks' and connections with miRNA and distal regions, revealing ideal drug targets. Such multi-layered heterogenous information integration assists in differentiating intra-level interconnections as well as inter-level edge types and node labels. Note that nodes in each layer are also arranged in 3D using 3D force-directed layout.

Example 3. Visualizing the global network of interactions while scaling or coloring a subset of the nodes based on their specific properties can enable hypothesis support. In this example, the visualization helps support the principle that functionally significant and highly conserved genes tend to be more central in physical protein-protein and regulatory networks[56]. Based on this hypothesis, Fig. 3C visualizes a network of tolerance to loss-of-function (LoF) mutations and evolutionary conservation, with nodes for (LoF) tolerant (blue) and essential genes (red) easily distinguishable [56]. Node size is based on degree centrality of a gene[7]. While essential genes tend to be bigger and central, LoF-tolerant genes are smaller and located in the periphery. Both the 2D snapshot (Fig. 3C) and 3D Supplementary Video 1 provide clear visualizations of this complex data that lead to easy interpretation. Note that we have published an iCAVE-generated visualization of a network with similar properties that enabled help support this hypothesis [57].

Graph Clustering To Identify Network Motifs. Clustering is critical in network exploration, as biomolecules that cluster together tend be functionally related. iCAVE offers the following graph clustering algorithms:

Edge-Betweenness clustering (EBC). The number of shortest paths going through a particular edge is EB. An edge with a high EB value connects multiple communities. At each step, the EBC algorithm removes the edge with the highest EB value until it has optimized a modularity metric on how unlikely the in-cluster degree of a node is in comparison to a random edge. EBC [58] is an attractive algorithm since it does not require an estimate of the number of clusters *a priori*, unlike a majority of existing graph clustering algorithms.

Markov clustering (MCL)[59] is a scalable and unsupervised algorithm which assumes that the number of intra-cluster connections is large and inter-cluster connections is small. It is based on a bootstrapping procedure

that simulates random walks (flow) through the network that expands or contracts in parallel with regional connectivity.

Modularity clustering (MC) uses the first eigenvector of the modularity matrix to assign nodes to clusters [60]. While ideal for weighted networks, MC delivers intuitive layouts for networks that do not have weights as well.

Layout Options for Cluster Visualization. iCAVE can easily visualize the clusters generated by iCAVE or another tool. By default, each cluster is positioned in space with *force-directed layout*[41], analogous to node positioning. Every cluster is embedded inside a transparent bubble, with members and their connections organized using the hemispherical layout. This arrangement provides a visual aesthetic, and (optional) edge bundling further clarifies the global topology (i.e. thicker bundles for high intra-cluster connectivity). Users can choose alternative layouts for cluster bubble positioning. *Lin-log cluster layout* is a variation of the force-directed model[41], where highly connected clusters are arranged in closer proximity.

Circos cluster layout is an innovative algorithm we developed as a 3D adaptation of the popular 2D Circos layout[61]. In this algorithm, we arrange the nodes in 3D space as in hemispherical layout, where the most connected node is located at the center of the hemisphere. We then slice the hemisphere with (pie-like) panels that correspond to separate clusters. Cluster representations can be optimized by variations in node/ slice colorings or edge bundling. Fig. 5 illustrates different cluster layout options using a metabolite network.

User Interface. Investigators can easily toggle between alternative layouts of a single graph to emphasize different network aspects. Users without stereo equipment can rotate, zoom or scale the visual to investigate special structures, print 2D snapshots and save movies of a rotating network. Rotation allows multiple views for users without 3D. Exporting and exchanging such movies is very convenient in the YouTube era, enabling easy publication and sharing with collaborators without iCAVE or stereo. Those with a stereo-enabled computer (or a CAVE facility) wear stereoscopic LCD shutter glasses that convey 3D image and allow immersive interaction. In a CAVE, sensors track the user's eye position and adjust perspective according to user movements. The mouse (or wand) gestures are mapped to logical events that the network layout application han-

dles. Zoom and rotate options activated with a simple mouse (or wand) click help focus on a particular node or edge.

Shape, color, texture for easier visual data dissemination. In iCAVE visualizations, 3D spherical glyphs represent nodes. Node color, size and texture optionally encode further statistics (e.g. color for gene induction or repression, size for the magnitude of change in expression, texture to differentiate classes). Edges can be colored, patterned or directed.

Input/output. Input to the iCAVE software is provided as a tab-delimited text file of identifiers and optional information on magnitude of change, edge directionality, edge weights, node/edge colors and patterns. Supplementary Table 2 includes the complete options list. Interaction data are read from an SQLite database. The user can modify the network in real time and store it in DB Browser (which is a light GUI editor for SQLite databases) as a .db file, so that it can be saved for later access. Output is the layout of the network drawn in VRUI environment, which can be saved as high-resolution 2D image snapshots (.png format) or movies (.gif format).

Discussion

iCAVE is a freely available open-source biomolecular network visualization tool that leverages advanced 3D and immersive 3D display technologies and offers several display options integrated with an effective user-interface. It incorporates a number of new and existing built-in network layout and graph clustering algorithms to enable automatic generation of 3D visualizations. Based on prior knowledge, input can additionally include (i) 3D node positions; (ii) cluster memberships; or (iii) multi-level hierarchies; or (iv) edge directionality. Utilizing iCAVE, investigators from diverse fields can gain insights from large, heterogeneous datasets, and optimize the quality of their visualizations using different node color, size, transparency options as well as various edge weight, thickness, transparency and directionality options. Network topological properties and centrality are also reported. While not extensive, its COMBO database enables disease researchers for quick query of their interactions among genes, drugs and disease phenotypes.

We designed iCAVE with a modular software structure to create a general and flexible community resource. Users with intermediate programming experience can add algorithms for network layout, cluster layout or graph

clustering without affecting the core functionality of the code. They can also add datasets to COMBO. Note that in future iterations of iCAVE, we will implement a Cytoscape [20] API. iCAVE is at the leading edge of immersive 3D network visualization. More user and case studies are needed to understand how best we can make use of immersion, stereoscopy and 3D. Other layouts could possibly work well within 3D or immersive 3D, which we will explore further in future studies. We hope that iCAVE will encourage both programmers and biologists to enter the world of 3D human-computer interfaces, in response to the growing demands in exploring large complex data and facilitate further developments.

Methods

Input/Output Formats. iCAVE supports tabular input formats (.txt, .csv, or .tsv). Interactions are either user-defined, or are queries of iCAVE COMBO database. Optional weights are represented with edge color frequency, directed edges with arrows, and node types with node glyph patterns. Input file options are listed in User's Manual. Networks are saved as static high-resolution (.png) images, or movies of the rotating 3D image (.gif).

Implementation. iCAVE uses Virtual Reality User Interface (VRUI), a development toolkit for interactive high performance VR applications[44], which enables quick and scalable production of completely platform-independent software. iCAVE is thus portable between Linux and Mac system computers (optionally equipped with stereo capabilities) and CAVE facilities.

Programming Libraries. Several programming libraries provide intuitive and user-friendly rendering solutions. Vrui library uses a C++ based OpenGL API platform that simplifies handling navigation transformations, light sources, menu creation, and rendering different objects. The SQLite3 software library handles large-scale database parsing. igraph library functions solve some of the programming challenges in generating regular and random graphs, manipulating graphs as well as assigning attributes to nodes and edges. The ANSI C programming language library Argtable enables parsing user-defined 3D graphics options.

Adding New Algorithms. Node and edge data are stored in two separate *structure* arrays. *Node structure* stores its id, name, number of neighbors, color, texture, cluster, size and coordinates. *Edge structure* includes start node id, end node id, weight and color. Storage with structure arrays simplifies the addition of new layout algorithms, because the arrays can be used as inputs. After layout coordinates are calculated, iCAVE utilizes OpenGL API for visualization. New algorithms are added as separate .cpp files and the corresponding header files are imported to the main program (vrnetview.cpp).

Label Creation. Since VRUI offers limited label creation options that render low quality and unreadable text, we developed texture mapping for high quality rendering. Supplementary Figure 1 illustrates VRUI vs. iCAVE labels.

User interface

Multiple functionalities demonstrate natural modes of interaction for effective analysis. These include activities such as selecting objects and interacting with the image in 3D space. While learning a new user-interface motif has been a traditional weakness of VR environments, more mature and practical technologies are becoming pervasive in consumer markets (e.g. motion sensors in Wii game consoles). These developments inform our user-interface design and provide new users with familiar gestures and interaction motifs.

Network exploration interface. Several features enable exploring, interacting and modulating the networks in real-time and saving the result. Interactive menu options are listed in Supplementary Table 2.

User interface in CAVE environments. Investigators enter a CAVE environment wearing stereoscopic LCD shutter glasses that convey 3D image. When the user walks around, sensors track movements and the video adjusts accordingly. Multiple users can exist simultaneously in the network and view the visualizations from multiple perspectives by moving in the space, or directly interact with specific biomolecules by clicking on the handheld device to display all its interactions in that network or stored in the database. User can alternatively investigate the network on his own computer.

Output image generation. iCAVE assembles image snapshots from several viewpoints into one high-resolution (.png) image (see Supplementary Fig. 2). The desired resolution is user-adjustable via a zoom factor.

Network Topological Properties

iCAVE automatically calculates the following network properties, rank-orders nodes based on these and represents their distribution both graphically and in tabular form:

Node degree property yields hubs. Generally, only a few biomolecules (hubs) have many network interactions[62,63]. Hubs are often central in mediating interactions among the less connected biomolecules [64],[65].

Neighborhood connectivity metric assists in identifying modularity, where small interconnected subgraphs may potentially represent specific enzymes, structures or processes[66,67] and provide significant insights to perturbed disease mechanisms. For example, the degree of gene co-expression correlates strongly with the complexity of an embedded motif[68].

Network average and local clustering coefficients quantify connectivity of the whole network or a single node. Local clustering coefficient is the ratio between the number of edges that connect the neighbors of a node versus the maximum possible number of edges. The network average clustering coefficient is the average of the local clustering coefficients of all nodes[69]. Only nodes that belong to networks with >3 nodes are considered. The range of coefficient values varies from 0 (no interconnection), to 1 (perfect interconnection).

Network closeness centrality and node closeness centrality quantifies the velocity of information flow within a network (the reciprocal sum of the shortest paths from a selected node to all other nodes[70]). Only nodes in subnetworks with >3 nodes are evaluated. When shortest paths are calculated, each edge is scaled

with corresponding weight, which can be a floating value. The average of all node closeness centrality values is the network closeness centrality value.

Network diameter is the length of shortest path between two farthest nodes. Unconnected nodes are not considered. Irregular networks usually have small diameters, while regular networks have large diameters.

Betweenness centrality is a global metric on the importance of a node, which is equal to the number of shortest paths from all vertices to all others that pass through that node, calculating the *load* on a node[71]. Real world scale-free networks usually involve short path lengths across the network, and a few nodes have high betweenness-centrality. *Connector* or *high-traffic* biomolecules that are vulnerable to targeted attacks, usually suggest potential non-hub drug targets[72],[73,74].

Shared nearest neighbors: A similarity metric based on the sharing of nearest neighbors between any two nodes. Particularly useful in network topology-based motif, sub-graph or cluster identification.

Shortest paths: Quantifies the importance of a node within the network, calculated by the number of shortest paths going through the node. Purely random graphs exhibit a small average shortest path length (~ the logarithm of the number of nodes) along with a small clustering coefficient.

Layout Algorithms.

A graph *G(V ={1, . . . , n},E)* represents a binary relation *E* over node set *V*. iCAVE both extends classical layouts to 3D and offers novel algorithms. Based on the underlying topology, a user can choose the best layout that helps with data interpretation.

1.Force-based layout. The forces acting on each node in classical Fruchterman-Rheingold (FR) algorithm[41] are:

$$f_a(ij) = \frac{d_{ij}^2}{k} \qquad f_r(ij) = -\frac{k^2}{d_{ij}} \qquad k = \sqrt[3]{\frac{volume}{number\ of\ nodes}}$$

where $f_a(ij)$ and $f_r(ij)$ are attractive and repulsive forces, d_{ij} is the distance between nodes *i* and *j*, and *k* is a constant corresponding to the equilibrium edge length.

2. Lin-log layouts. We used r-PloyLog[48] energy model to implement the node-repulsion and edge-repulsion LinLog models. For all $r \in R$ with $r > 0$, the node-repulsion energy of a layout *p* is:

$$U_{r-NodePolyLog}(p) = \sum_{\{u,v\}\in E} \frac{1}{r} \| p(u) - p(v) \|^r - \sum_{\{u,v\}\in V^2} \ln \| p(u) - p(v) \|$$

where *p(u)* is the position of node *u*. Edge-repulsion energy is:

$$U_{r-EdgePolyLog}(p) = \sum_{\{u,v\}\in E} \frac{1}{r} \| p(u) - p(v) \|^r - \sum_{\{u,v\}\in V^2} \deg(u)\deg(v) \ln \| p(u) - p(v) \|$$

where *deg(u)* is the number of edges incident to node *u*. At *r=3*, the 3-PolyLog reduces to FR and at *r=1* to LinLog model. LinLog models group nodes according to cut density and the normalized cut, therefore the layout leads to graph clustering.

3. Hemispherical layout. We place *n* nodes of a graph *G(V= {1,..,n}, E)* equally spaced on a single 3D hemisphere surface, reducing the problem to finding a *hemispherical node ordering*. Coordinates for a node *i* ϵ *V* are (x_i, y_i, z_i) ϵ *R*, at fixed hemisphere radius R:

$$x_i = R * \cos(latitude_i) * \cos(longitude_i) \qquad 0° \le longitude \le 360°$$

$y_i = R * \sin(longitude_i) * \cos(latitude_i) \qquad 0° \leq latitude \leq 90°$

$z_i = R * \sin(latitude_i) \qquad 0° \leq i \leq \#\ of\ nodes$

Nodes are sorted and placed based on their degree, with the highest degree node at the hemisphere surface center. Algorithm inputs are the number of nodes, the graph center position and hemisphere radius. Hemisphere radius, node sizes, colors and textures are adjustable.

4. Semantic levels layout is ideal for integrative analysis of multiple data resources (e.g. genotype, phenotype, drugs, proteins, metabolites). Initially, FR algorithm is performed in 2D. Then, multiple equidistant levels (default =7) are created in the z-dimension. Based on network topology, we consecutively assign the nodes to one of the layers. iCAVE user-interface allows the manual manipulation of the number of layers and the distance between them. If layers are not predefined, we suggest experimenting with different options.

5. Hybrid force directed layout[49] Original version of this algorithm is extremely computationally intensive, so we implemented a simplified version, reducing the run time at the expense of visualization quality. Our version has three steps: (i) position nodes randomly; (ii) partition the resulting graph; (iii) apply FR[41] algorithm separately on each subgraph. The partitioning step splits the graph into two sub-graphs (A and B) of equal sizes. This requires minimizing the cut size, by calculating the second Eigenvector (Fiedler vector) λ of the following:

$L(G)\vec{q} = \lambda\vec{q}$ where

$$\vec{q} = \begin{pmatrix} q_1 \\ q_2 \\ \vdots \\ q_n \end{pmatrix}; \quad q_i = \begin{cases} 1 & \forall i \in A \\ -1 & \forall i \in B \end{cases} \ and\ n \equiv \#\ of\ nodes$$

and *L(G)* is the Laplacian of graph *G*. The power-iteration algorithm solves for λ.

Edge-Betweenness (EB) Clustering Algorithm: An edge with a high *EB* value potentially connects two or more *communities*. The edge with the highest *EB* value is removed at each step. The number of edges to be removed is user-defined (with a default of *0.2 times the number of edges)*. Any edge that leads to a single-node cluster is not removed.

Edge bundling algorithm is based on application of forces (electrostatic and spring) on an edge subdivided into multiple points. Edge compatibility metrics edge angle, scale (length), position and visual compatibility are multiplied for total compatibility. If two edges are compatible above a threshold, forces are calculated and added to each subdivision, and those subdivisions are bundled together.

Project name: iCAVE

Project home page: http://research.mssm.edu/gumuslab/software.html

Operating system(s): Unix, Linux, macOS

Programming Language: C++

Other requirements: For macOS: XCode, X11/XQuartz, libjpeg, libz, libpng

License: GNU Lesser General Public License

Any restrictions to use by non-academics: License needed

Supporting Data and Materials

Software, user manual and tutorial are freely available for download to academic users at http://research.mssm.edu/gumuslab/software.html released under the GNU Lesser General Public License. *We will host the software at the GigaScience GitHub repository, if available.

Declarations

Consent for publication. All authors consent for publication.

Competing interests. None declared.

Funding. This work was supported by the Concern Foundation Conquer Cancer Now Award (to Z.H.G) and computational resources provided by the Department of Scientific Computing at the Icahn School of Medicine at Mount Sinai, and PBTECH staff expertise of HRH Prince Alwaleed Bin Talal Bin Abdulaziz Alsaud Institute for Computational Biomedicine and the computational resources of the Coffrin Center for Biomedical Information at Weill Cornell Medical College of Cornell University (to V.L. and Z.H.G).

Author's Contributions. ZHG conceived and designed the study. VL, SK and ZHG contributed to data collection, analysis and interpretation. All authors contributed to software development. VL, SK and ZHG contributed to drafting and critical revisions of the article. All authors approve the final version.

Acknowledgements. We thank Jian Sun and Jason Banfelder for programming assistance and comments. To ensure that iCAVE meets the needs of biomedical researchers, we relied heavily on the advice, feedback and discussions from representative end-users including Alex Lash, Michael LeVine, Chris Sander, Eric Schadt and Karsten Suhre.

References

1. Newman MEJ. Networks: An Introduction. Oxford University Press, USA.; 2006.

2. Caldarelli G. Scale-Free Networks:Complex Webs in Nature and Technology. Oxford Univ. Press. USA. Oxford University Press, USA.; 2007.

3. Dorogovtsev SN, Mendes JFF, Samukhin AN, Zyuzin AY. Organization of modular networks. Phys. Rev. E. Stat. Nonlin. Soft Matter Phys. [Internet]. 2008 [cited 2012 Sep 14];78:056106. Available from: http://www.ncbi.nlm.nih.gov/pubmed/19113189

4. Salwinski L, Eisenberg D. Computational methods of analysis of protein-protein interactions. Curr. Opin. Struct. Biol. [Internet]. 2003 [cited 2012 Sep 14];13:377–82. Available from: http://www.ncbi.nlm.nih.gov/pubmed/12831890

5. Yook S-H, Oltvai ZN, Barabási A-L. Functional and topological characterization of protein interaction networks. Proteomics [Internet]. 2004 [cited 2012 Jul 19];4:928–42. Available from: http://www.ncbi.nlm.nih.gov/pubmed/15048975

6. Taylor IW, Linding R, Warde-Farley D, Liu Y, Pesquita C, Faria D, et al. Dynamic modularity in protein interaction networks predicts breast cancer outcome. Nat. Biotechnol. [Internet]. 2009 [cited 2010 Jul 14];27:199–204. Available from: http://www.ncbi.nlm.nih.gov/pubmed/19182785

7. Benson M, Breitling R. Network theory to understand microarray studies of complex diseases. Curr. Mol. Med. [Internet]. 2006 [cited 2012 Sep 14];6:695–701. Available from: http://www.ncbi.nlm.nih.gov/pubmed/17022739

8. Calvano SE, Xiao W, Richards DR, Felciano RM, Baker H V, Cho RJ, et al. A network-based analysis of systemic inflammation in humans. Nature [Internet]. 2005 [cited 2012 Jul 13];437:1032–7. Available from: http://www.ncbi.nlm.nih.gov/pubmed/16136080

9. Gao J, Aksoy BA, Dogrusoz U, Dresdner G, Gross B, Sumer SO, et al. Integrative analysis of complex cancer genomics and clinical profiles using the cBioPortal. Sci. Signal. [Internet]. 2013 [cited 2014 Jul 15];6:pl1. Available from: http://stke.sciencemag.org/content/6/269/pl1

10. Zhu J, Zhang B, Schadt EE. A systems biology approach to drug discovery. Adv. Genet. [Internet]. 2008 [cited 2012 Jul 29];60:603–35. Available from: http://www.ncbi.nlm.nih.gov/pubmed/18358334

11. Brehme M, Hantschel O, Colinge J, Kaupe I, Planyavsky M, Köcher T, et al. Charting the molecular network of the drug target Bcr-Abl. Proc. Natl. Acad. Sci. U. S. A. [Internet]. 2009 [cited 2012 Jul 16];106:7414–9. Available from: http://www.pubmedcentral.nih.gov/articlerender.fcgi?artid=2670881&tool=pmcentrez&rendertype=abstract

12. Erler JT, Linding R. Network medicine strikes a blow against breast cancer. Cell [Internet]. 2012 [cited 2012 Jul 23];149:731–3. Available from: http://www.ncbi.nlm.nih.gov/pubmed/22579276

13. Gorin MA, Pan Q. Protein kinase C epsilon: an oncogene and emerging tumor biomarker. Mol. Cancer [Internet]. 2009 [cited 2012 Aug 5];8:9. Available from: http://www.pubmedcentral.nih.gov/articlerender.fcgi?artid=2647895&tool=pmcentrez&rendertype=abstract

14. Dudley JT, Butte AJ. Identification of discriminating biomarkers for human disease using integrative

network biology. Pac. Symp. Biocomput. [Internet]. 2009 [cited 2012 Sep 14];27–38. Available from: http://www.pubmedcentral.nih.gov/articlerender.fcgi?artid=2749008&tool=pmcentrez&rendertype=abstract

15. Jin G, Zhou X, Wang H, Zhao H, Cui K, Zhang X-S, et al. The knowledge-integrated network biomarkers discovery for major adverse cardiac events. J. Proteome Res. [Internet]. 2008 [cited 2012 Sep 14];7:4013–21. Available from: http://www.pubmedcentral.nih.gov/articlerender.fcgi?artid=2854538&tool=pmcentrez&rendertype=abstract

16. Ou K, Yu K, Kesuma D, Hooi M, Huang N, Chen W, et al. Novel breast cancer biomarkers identified by integrative proteomic and gene expression mapping. J. Proteome Res. [Internet]. 2008 [cited 2012 Sep 14];7:1518–28. Available from: http://www.ncbi.nlm.nih.gov/pubmed/18318472

17. Gehlenborg N, O'Donoghue SI, Baliga NS, Goesmann A, Hibbs M a, Kitano H, et al. Visualization of omics data for systems biology. Nat. Methods [Internet]. 2010;7:S56–68. Available from: http://www.ncbi.nlm.nih.gov/pubmed/20195258

18. Pavlopoulos GA, Malliarakis D, Papanikolaou N, Theodosiou T, Enright AJ, Iliopoulos I. Visualizing genome and systems biology: technologies, tools, implementation techniques and trends, past, present and future. Gigascience [Internet]. 2015 [cited 2016 Jan 25];4:38. Available from: http://www.pubmedcentral.nih.gov/articlerender.fcgi?artid=4548842&tool=pmcentrez&rendertype=abstract

19. Bader GD, Cary MP, Sander C. Pathguide: a pathway resource list. Nucleic Acids Res. [Internet]. 2006;34:D504–6. Available from: http://nar.oxfordjournals.org/content/34/suppl_1/D504.long

20. Shannon P, Markiel A, Ozier O, Baliga NS, Wang JT, Ramage D, et al. Cytoscape: a software environment for integrated models of biomolecular interaction networks. Genome Res. 2003;13:2498–504.

21. Yeung N, Cline MS, Kuchinsky A, Smoot ME, Bader GD. Exploring biological networks with Cytoscape software. Curr. Protoc. Bioinformatics [Internet]. 2008 [cited 2012 Sep 19];Chapter 8:Unit 8.13. Available from: http://www.ncbi.nlm.nih.gov/pubmed/18819078

22. www.ingenuity.com.

23. Breitkreutz B-J, Stark C, Tyers M. Osprey: a network visualization system. Genome Biol. [Internet]. 2003 [cited 2012 Sep 19];4:R22. Available from: http://www.pubmedcentral.nih.gov/articlerender.fcgi?artid=153462&tool=pmcentrez&rendertype=abstract

24. Hu Z, Mellor J, Wu J, Yamada T, Holloway D, Delisi C. VisANT: data-integrating visual framework for biological networks and modules. Nucleic Acids Res. [Internet]. 2005 [cited 2012 Jul 17];33:W352–7. Available from: http://www.pubmedcentral.nih.gov/articlerender.fcgi?artid=1160192&tool=pmcentrez&rendertype=abstract

25. Gerasch A, Faber D, Küntzer J, Niermann P, Kohlbacher O, Lenhof H-P, et al. BiNA: a visual analytics tool for biological network data. PLoS One [Internet]. 2014 [cited 2016 Feb 4];9:e87397. Available from: http://www.pubmedcentral.nih.gov/articlerender.fcgi?artid=3923765&tool=pmcentrez&rendertype=abstract

26. Schadt EE, Linderman MD, Sorenson J, Lee L, Nolan GP. Computational solutions to large-scale data management and analysis. Nat. Rev. Genet. [Internet]. 2010 [cited 2016 Oct 16];11:647–57. Available from: http://www.ncbi.nlm.nih.gov/pubmed/20717155

27. Greffard N, Picarougne F, Kuntz P. Beyond the classical monoscopic 3D in graph analytics: An experimental study of the impact of stereoscopy. 2014 IEEE VIS Int. Work. 3DVis [Internet]. IEEE; 2014 [cited 2016 Oct 7]. p. 19–24. Available from: http://ieeexplore.ieee.org/document/7160095/

28. Ware C, Mitchell P. Visualizing graphs in three dimensions. ACM Trans. Appl. Percept. [Internet]. 2008;5:1–15. Available from: http://portal.acm.org/citation.cfm?id=1279640.1279642

29. Sollenberger RL, Milgram P. Effects of stereoscopic and rotational displays in a three-dimensional path-tracing task. Hum. Factors [Internet]. 1993 [cited 2016 Oct 12];35:483–99. Available from: http://www.ncbi.nlm.nih.gov/pubmed/8244411

30. Kwon O-H, Muelder C, Lee K. A study of layout, rendering, and interaction methods for immersive graph visualization. IEEE Trans. Vis. Comput. Graph. 2016;1802–15.

31. Bhavnani SK, Ganesan A, Hall T, Maslowski E, Eichinger F, Martini S, et al. Discovering hidden relationships between renal diseases and regulated genes through 3D network visualizations. BMC Res. Notes [Internet]. 2010;3:296. Available from: http://www.biomedcentral.com/1756-0500/3/296

32. Ruths DA, Chen ES, Ellis L. Arbor 3D: an interactive environment for examining phylogenetic and taxonomic trees in multiple dimensions. Bioinformatics [Internet]. 2000 [cited 2012 Sep 19];16:1003–9. Available from: http://www.ncbi.nlm.nih.gov/pubmed/11159311

33. Quon GT, Gordon P, Sensen CW. 4D bioinformatics: a new look at the ribosome as an example. IUBMB

Life [Internet]. [cited 2012 Sep 19];55:279–83. Available from: http://www.ncbi.nlm.nih.gov/pubmed/12880210

34. Turinsky AL, Fanea E, Trinh Q, Wat S, Hallgrímsson B, Dong X, et al. CAVEman: Standardized anatomical context for biomedical data mapping. Anat. Sci. Educ. [Internet]. 2008 [cited 2012 Sep 19];1:10–8. Available from: http://www.ncbi.nlm.nih.gov/pubmed/19177373

35. Yang Y, Engin L, Wurtele ES, Cruz-Neira C, Dickerson JA. Integration of metabolic networks and gene expression in virtual reality. Bioinformatics [Internet]. 2005 [cited 2012 Sep 19];21:3645–50. Available from: http://www.ncbi.nlm.nih.gov/pubmed/16020466

36. http://nvidia.com/get3D.

37. Pavlopoulos GA, O'Donoghue SI, Satagopam VP, Soldatos TG, Pafilis E, Schneider R. Arena3D: visualization of biological networks in 3D. BMC Syst. Biol. [Internet]. 2008 [cited 2011 Aug 1];2:104. Available from: http://www.pubmedcentral.nih.gov/articlerender.fcgi?artid=2637860&tool=pmcentrez&rendertype=abstract

38. Wang Q, Tang B, Song L, Ren B, Liang Q, Xie F, et al. 3DScapeCS: application of three dimensional, parallel, dynamic network visualization in Cytoscape. BMC Bioinformatics [Internet]. 2013;14:322. Available from: http://www.pubmedcentral.nih.gov/articlerender.fcgi?artid=3835703&tool=pmcentrez&rendertype=abstract

39. Freeman TC, Goldovsky L, Brosch M, van Dongen S, Mazière P, Grocock RJ, et al. Construction, visualisation, and clustering of transcription networks from microarray expression data. PLoS Comput. Biol. [Internet]. 2007 [cited 2012 Aug 3];3:2032–42. Available from: http://www.pubmedcentral.nih.gov/articlerender.fcgi?artid=2041979&tool=pmcentrez&rendertype=abstract

40. Cerami E, Demir E, Schultz N, Taylor BS, Sander C. Automated network analysis identifies core pathways in glioblastoma. PLoS One. 2010;5:e8918.

41. Fruchterman TMJ, Reingold EM. Graph drawing by force-directed placement. Software—Practice Exp. 1991;21:1129–64.

42. Ellis SR, Tharp GK, Grunwald AJ, Smith S. Exocentric Judgements in Real Environments and Stereoscopic Displays. Proc. Hum. Factors Ergon. Soc. Annu. Meet. [Internet]. SAGE PublicationsSage CA: Los Angeles, CA; 1991 [cited 2017 Jan 13];35:1442–6. Available from:

http://pro.sagepub.com/lookup/doi/10.1177/154193129103502005

43. Etemadpour R, Monson E, Linsen L. The Effect of Stereoscopic Immersive Environments on Projection-Based Multi-dimensional Data Visualization. 2013 17th Int. Conf. Inf. Vis. [Internet]. IEEE; 2013 [cited 2016 Oct 12]. p. 389–97. Available from: http://ieeexplore.ieee.org/document/6676591/

44. Stephen G. Eick SGE. Aspects of Network Visualization. [cited 2012 Sep 25]; Available from: http://citeseerx.ist.psu.edu/viewdoc/summary?doi=10.1.1.42.8448

45. Wagemans J, Elder JH, Kubovy M, Palmer SE, Peterson MA, Singh M, et al. A century of Gestalt psychology in visual perception: I. Perceptual grouping and figure-ground organization. Psychol. Bull. [Internet]. 2012 [cited 2016 Jan 10];138:1172–217. Available from: http://www.pubmedcentral.nih.gov/articlerender.fcgi?artid=3482144&tool=pmcentrez&rendertype=abstract

46. Holten D, van Wijk JJ. Force-Directed Edge Bundling for Graph Visualization.. Comput. Graph. Forum, Proc. EuroVis 2009. 2009;28:983–90.

47. Kamada T, Kawai S. An algorithm for drawing general undirected graphs. . Inf. Process. Lett. 1989;31:7–15.

48. Noack A. Energy models for graph clustering. J. Graph Algorithms Appl. 2007;11:453–80.

49. Frishman Y, Tal A. Multi-level graph layout on the GPU. IEEE Trans. Vis. Comput. Graph. [Internet]. [cited 2012 Nov 12];13:1310–9. Available from: http://www.ncbi.nlm.nih.gov/pubmed/17968079

50. Butcher EC, Berg EL, Kunkel EJ. Systems biology in drug discovery. Nat. Biotechnol. [Internet]. 2004 [cited 2012 Jul 13];22:1253–9. Available from: http://www.ncbi.nlm.nih.gov/pubmed/15470465

51. Chanda SK, Caldwell JS. Fulfilling the promise: drug discovery in the post-genomic era. Drug Discov. Today [Internet]. 2003 [cited 2012 Sep 25];8:168–74. Available from: http://www.ncbi.nlm.nih.gov/pubmed/12581711

52. Searls DB. Pharmacophylogenomics: genes, evolution and drug targets. Nat. Rev. Drug Discov. [Internet]. 2003 [cited 2012 Jul 30];2:613–23. Available from: http://www.ncbi.nlm.nih.gov/pubmed/12904811

53. Yildirim MA, Goh K-I, Cusick ME, Barabási A-L, Vidal M. Drug-target network. Nat. Biotechnol. [Internet]. 2007 [cited 2012 Jul 13];25:1119–26. Available from: http://www.ncbi.nlm.nih.gov/pubmed/17921997

54. Gandy S, Haroutunian V, DeKosky ST, Sano M, Schadt EE. CR1 and the “vanishing amyloid” hypothesis of

Alzheimer's disease. Biol. Psychiatry [Internet]. 2013 [cited 2013 Mar 27];73:393–5. Available from: http://www.pubmedcentral.nih.gov/articlerender.fcgi?artid=3600375&tool=pmcentrez&rendertype=abstract

55. Gerstein MB, Kundaje A, Hariharan M, Landt SG, Yan K-K, Cheng C, et al. Architecture of the human regulatory network derived from ENCODE data. Nature [Internet]. 2012 [cited 2013 Feb 28];489:91–100. Available from: http://www.ncbi.nlm.nih.gov/pubmed/22955619

56. Khurana E, Fu Y, Chen J, Gerstein M. Interpretation of genomic variants using a unified biological network approach. PLoS Comput. Biol. [Internet]. 2013 [cited 2016 Feb 14];9:e1002886. Available from: http://www.pubmedcentral.nih.gov/articlerender.fcgi?artid=3591262&tool=pmcentrez&rendertype=abstract

57. E K, Y F, V C, XJ M, HM K, T L, et al. Integrative annotation of variants form 1, 092 humans: application to cancer genomics. Science (80-.). 2013;

58. Girvan M, Newman MEJ. Community structure in social and biological networks. Proc. Natl. Acad. Sci. U. S. A. [Internet]. 2002 [cited 2012 Jul 17];99:7821–6. Available from: http://www.pnas.org/content/99/12/7821

59. van Dongen S. Graph Clustering by Flow Simulation. University of Utrecht; 2000.

60. Newman MEJ. Modularity and community structure in networks. Proc. Natl. Acad. Sci. U. S. A. [Internet]. 2006 [cited 2012 Nov 2];103:8577–82. Available from: http://www.pnas.org/content/103/23/8577.abstract

61. Krzywinski M, Schein J, Birol I, Connors J, Gascoyne R, Horsman D, et al. Circos: an information aesthetic for comparative genomics. Genome Res. [Internet]. 2009 [cited 2011 May 11];19:1639–45. Available from: http://genome.cshlp.org/cgi/content/abstract/gr.092759.109v1

62. Barabasi A, Albert R. Emergence of scaling in random networks. Science [Internet]. 1999 [cited 2012 Jul 23];286:509–12. Available from: http://www.ncbi.nlm.nih.gov/pubmed/10521342

63. Albert R. Scale-free networks in cell biology. J. Cell Sci. [Internet]. 2005 [cited 2012 Jul 17];118:4947–57. Available from: http://www.ncbi.nlm.nih.gov/pubmed/16254242

64. Jeong H, Mason SP, Barabási AL, Oltvai ZN. Lethality and centrality in protein networks. Nature [Internet]. 2001 [cited 2012 Jul 26];411:41–2. Available from: http://www.ncbi.nlm.nih.gov/pubmed/11333967

65. Jonsson PF, Bates PA. Global topological features of cancer proteins in the human interactome. Bioinformatics [Internet]. 2006 [cited 2012 Jul 14];22:2291–7. Available from: http://www.pubmedcentral.nih.gov/articlerender.fcgi?artid=1865486&tool=pmcentrez&rendertype=abstract

66. Milo R, Shen-Orr S, Itzkovitz S, Kashtan N, Chklovskii D, Alon U. Network motifs: simple building blocks of complex networks. Science [Internet]. 2002 [cited 2012 Jul 13];298:824–7. Available from: http://www.ncbi.nlm.nih.gov/pubmed/12399590

67. Shen-Orr SS, Milo R, Mangan S, Alon U. Network motifs in the transcriptional regulation network of Escherichia coli. Nat. Genet. [Internet]. 2002 [cited 2012 Jul 14];31:64–8. Available from: http://dx.doi.org/10.1038/ng881

68. Bhardwaj N, Lu H. Co-expression among constituents of a motif in the protein-protein interaction network. J. Bioinform. Comput. Biol. [Internet]. 2009 [cited 2012 Sep 26];7:1–17. Available from: http://www.pubmedcentral.nih.gov/articlerender.fcgi?artid=2770376&tool=pmcentrez&rendertype=abstract

69. Watts DJ, Strogatz SH. Collective dynamics of "small-world" networks. Nature [Internet]. 1998 [cited 2013 Feb 28];393:440–2. Available from: http://www.ncbi.nlm.nih.gov/pubmed/9623998

70. Newman MEJ. A measure of betweenness centrality based on random walks. 2003 [cited 2013 Apr 23];15. Available from: http://arxiv.org/abs/cond-mat/0309045

71. Brandes U. A Faster Algorithm for Betweenness Centrality. J. Math. Sociol. 2001;25:163–77.

72. Csermely P, Agoston V, Pongor S. The efficiency of multi-target drugs: the network approach might help drug design. Trends Pharmacol. Sci. [Internet]. 2005 [cited 2012 Jul 16];26:178–82. Available from: http://www.ncbi.nlm.nih.gov/pubmed/15808341

73. Joy MP, Brock A, Ingber DE, Huang S. High-betweenness proteins in the yeast protein interaction network. J. Biomed. Biotechnol. [Internet]. 2005 [cited 2012 Jul 31];2005:96–103. Available from: http://www.pubmedcentral.nih.gov/articlerender.fcgi?artid=1184047&tool=pmcentrez&rendertype=abstract

74. Yu H, Kim PM, Sprecher E, Trifonov V, Gerstein M. The importance of bottlenecks in protein networks: correlation with gene essentiality and expression dynamics. PLoS Comput. Biol. [Internet]. 2007 [cited 2012 Jul 13];3:e59. Available from: http://www.pubmedcentral.nih.gov/articlerender.fcgi?artid=1853125&tool=pmcentrez&rendertype=abstract

75. Krumsiek J, Suhre K, Illig T, Adamski J, Theis FJ. Gaussian graphical modeling reconstructs pathway reactions from high-throughput metabolomics data. BMC Syst. Biol. 2011;5:21.

Figures

Fig. 1. Comparison of Displays. **a**. User interacting with a flat 2D display of manually curated pathways affected by genomic alterations in glioblastoma [40]. **b**. User experiencing full 3D depth perception of the same network with iCAVE using stereoscopic glasses on his desktop. iCAVE display is generated with force-directed layout algorithm. The visual clutter problem due to edge crossings that create a hairball effect in 2D are eliminated, as the user can navigate to multiple viewpoints. **c.** A screenshot from the 3D display generated with the force-directed layout. This network is generated without *a priori* knowledge of the underlying biology; rotating the layout helps readily identifying hubs, connectors and modules, such as the connectors between two dense regions of the network (highlighted with a (*) in both panels **a** and **c**). **d**. Immersive visualization in a CAVE environment, with user inside data space. While the photos only capture images reflected on the interaction walls of the CAVE, user experiences a virtual 3D image. In both **b** and **d**, zoom and rotation options help users focus on a particular hub or module. While the addition of the third dimension gives a richer, more intuitive and ultimately more meaningful understanding of the network-represented data, the 3D layout brings a new modality into network visualization design, with clear layouts.

Fig. 2. a. iCAVE visualization of bacterial leucine transporter, LeuT residue correlation network, side-view. Nodes represent 3D coordinates of alpha-carbon of a residue; edges represent top 3,000 (Pearson) correlations between residue pairs, where the input is 3D coordinates & correlation scores. Surprisingly, 3D visualization with edge bundling enables representation of highest density correlations (*correlation highways*) that travel through the substrate permeation pore in protein center, connecting extracellular and intracellular domains. Correlation highways at the pore are visually fascinating and biophysically intuitive; some residues outside the pore reveal unexpected structural importance (data courtesy of Harel Weinstein). **b**. **Living Human Brain Connectivity**. iCAVE visualization of brain regions as nodes, labeled by anatomical region name. Edges show connectivity, and bundling shows *connectivity highways*. Datasets from Diffusion Tensor Imaging of left hemisphere scanned ith Siemens 1.5Tesla and generated by Fiber Assignment by Continuous Tracking tractography using U. of California, Los Angeles (UCLA) Multimodal Connectivity Package, connectivity matrix mod-

ule. Database is powered by the Human Connectome Project, which compiles neural data to achieve never before realized conclusions on the living human brain.

Fig. 3. iCAVE print-ready images of networks in 2D with white background. a. Large probabilistic causal network constructed from human omental adipose tissue in a morbidly obese patient cohort (7,601 nodes, 13,979 edges)[54]. Nodes are gene expression traits in tissue; edges are derived from a Bayesian network reconstruction algorithm that leverages DNA variation for causality. Highlighted nodes represent gene signature causally associated with disease variants or pathways. Signature genes cluster together, suggesting functional relatedness. **b.** Network of 119 transcription factors (TFs), their 26,037 target interactions (edges) with 9,057 genes (nodes)[55] from ENCODE study. **c.** Massive unified 'Multinet' of Protein-Protein Interaction (PPI), phosphorylation, metabolic, signaling, genetic and regulatory networks (14,558 nodes, 109,597 edges). Multinet correlates tolerance to loss-of-function (LoF) mutations and evolutionary conservation, with nodes for (LoF) tolerant (blue) and essential genes (red) easily distinguishable. Node size is based on the degree centrality of a gene. Essential genes tend to be bigger and central and LoF-tolerant genes are smaller in the periphery. **d.** Hierarchical network integrates TF, ncRNA, miRNA and PPI data. Hierarchy levels are based on the mutual relationships between TFs. Connectivity and hierarchy reflects genomic properties (top level TF-binding correlates with target expression; mid-level contains information flow bottlenecks and connections with miRNA and distal regions, revealing ideal drug targets) (data from: Mark Gerstein, personal communication). While a 2D figure cannot display the interconnections between elements within the same hierarchical level, it is straightforward with iCAVE semantic levels layout.

Fig. 4. Visualizing Multiple Layers of Information. a. Using the iCAVE interface, we can pick a gene of interest (e.g. AHR, dark blue), and query the COMBO database for diseases that have been associated with AHR variants from GWAS studies (purple); drugs that are known to directly target AHR (green); and drug candidates that may directly interact with AHR (light blue). These drugs serve as an initial screening list of candidates for subsequent AHR binding site characterization. Semantics Levels layout segregates the layers. **b.** We can further query the COMBO database to generate a protein-protein interaction map of AHR (dark blue

nodes; middle layer) and visualize the diseases associated with known SNPs (Single Nucleotide Polymorphisms) in genes that code for AHR-interacting proteins (purple) and drugs that directly target them (green). We provide a more detailed movie of this three-level semantics network with legible disease, gene and drug names in the Supplementary Video 3. For both Panels, users can click on any edge or node for further information (e.g. exact disease variant location from GWAS studies).

Fig. 5. Pathway reconstructed high-throughput metabolomics data with Gaussian Graphical Modeling (GGM)[75]; each sphere color represents a single metabolite class**. a.** The force-directed layout of the weighted network captures the local cluster structures (snapshot). **b.** Snapshot of user-defined metabolite clusters: cluster layout is force-directed while inside each cluster, nodes are ordered in hemispherical layout. Edge bundling represents inter-cluster connectivity strength. **c**. User-defined clusters of the same network in a Circos layout. We provide a movie to better investigate the network in the Supplementary Video 4. **d**. Markov Chain Clustering of the same network based on its connectivity, available as one of the clustering options in iCAVE. Each cluster is represented inside spherical bubble. While topology suggests that most similar metabolites cluster together, this is not always the case, as shown. In all panels, addition of metabolite labels is user-optional.

Figure 1 Click here to download Figure Figure_1Gumus.tif

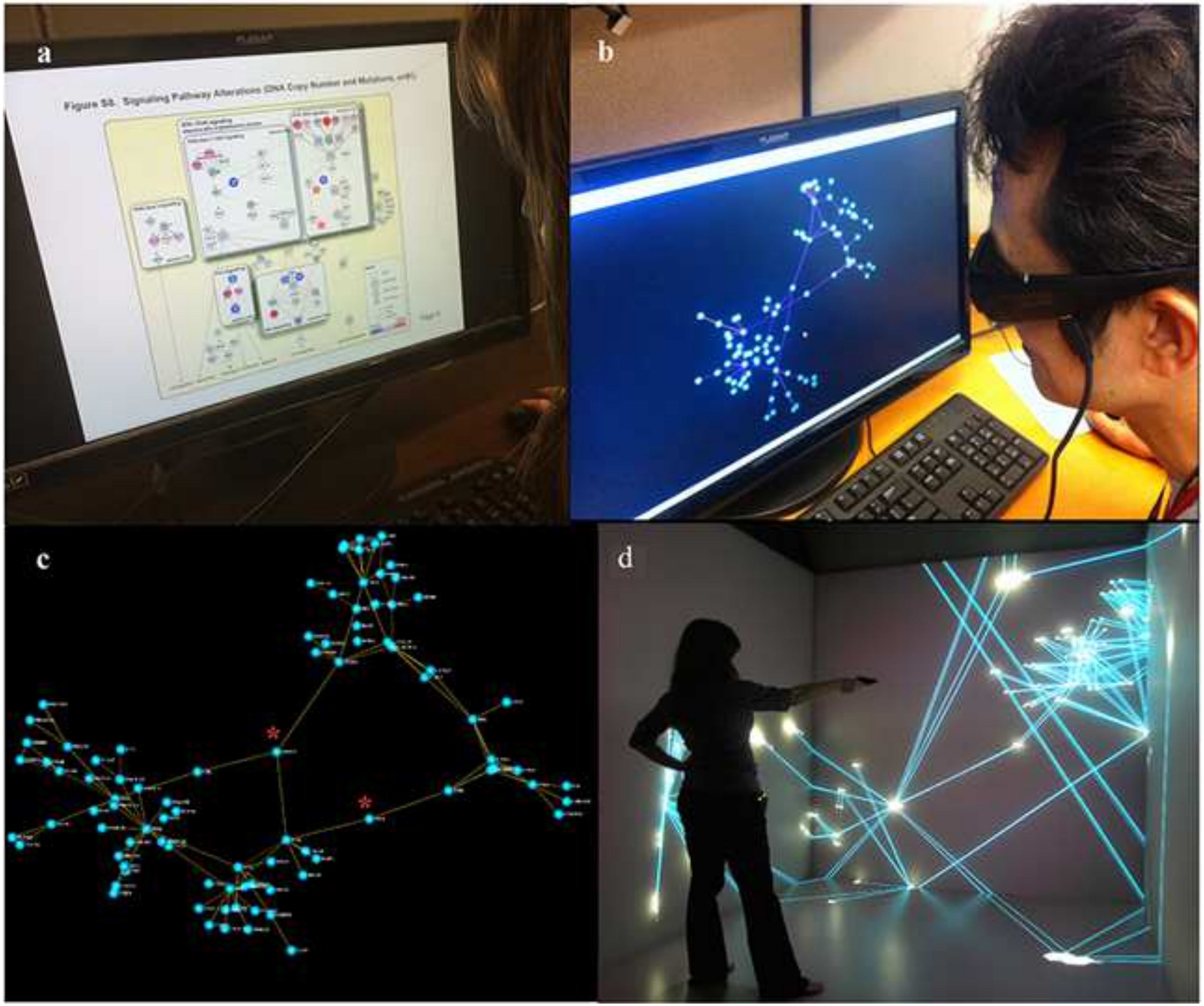

Click here to download Figure Figure-2Gumus.tif

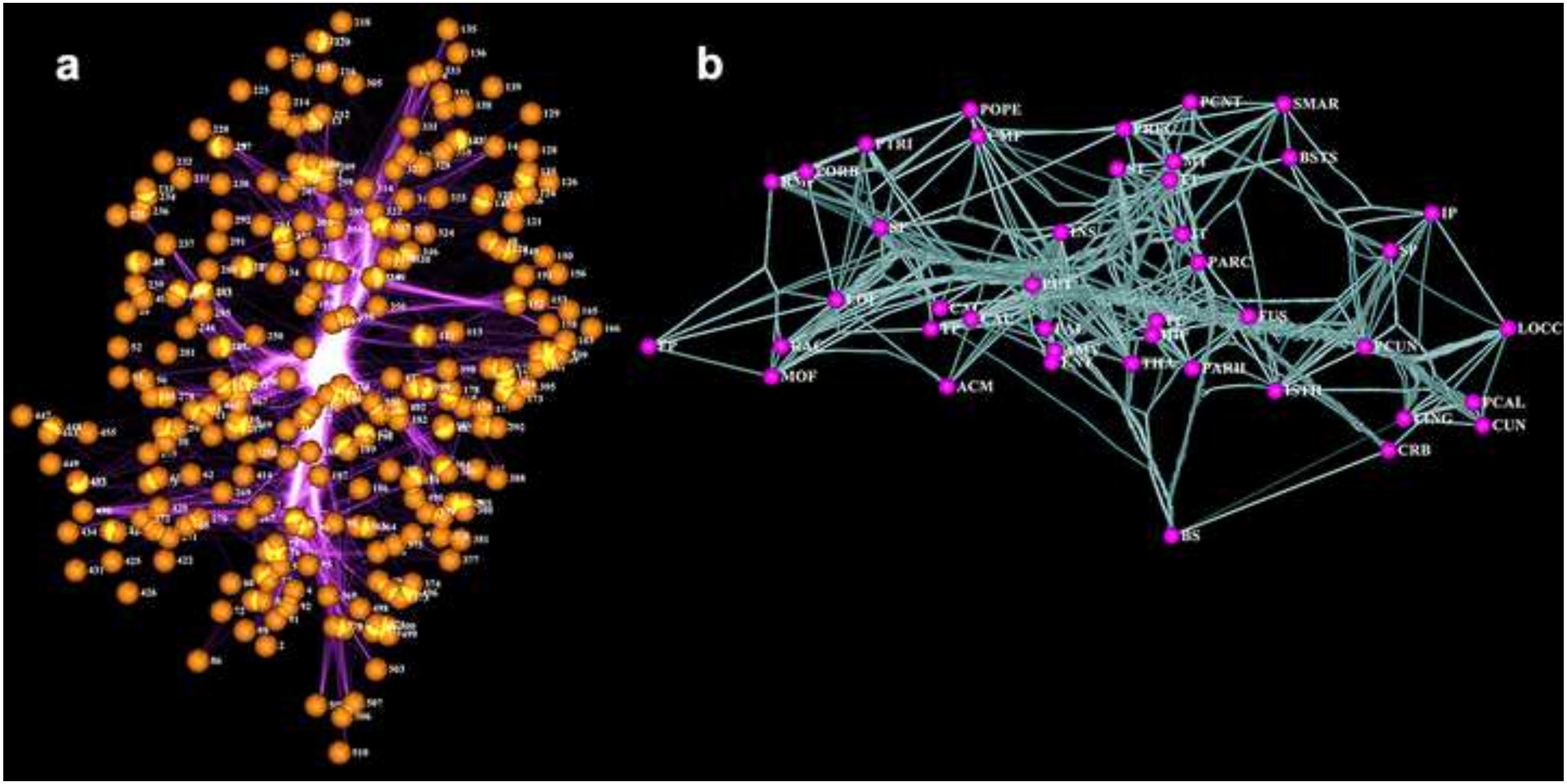

Figure 3

Click here to download Figure Figure-4Gumus.tif

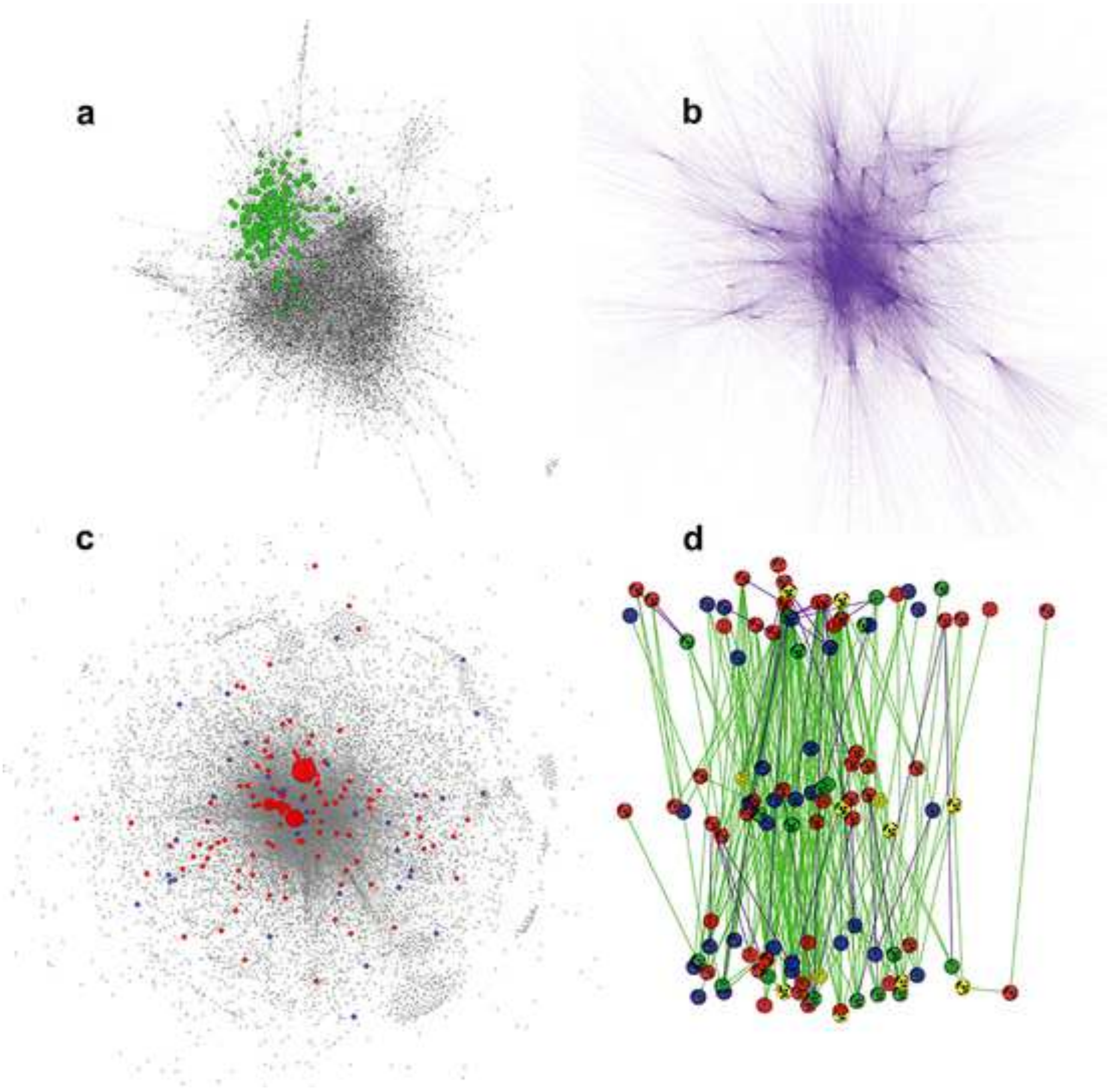

Click here to download Figure Figure-5Gumus.tif

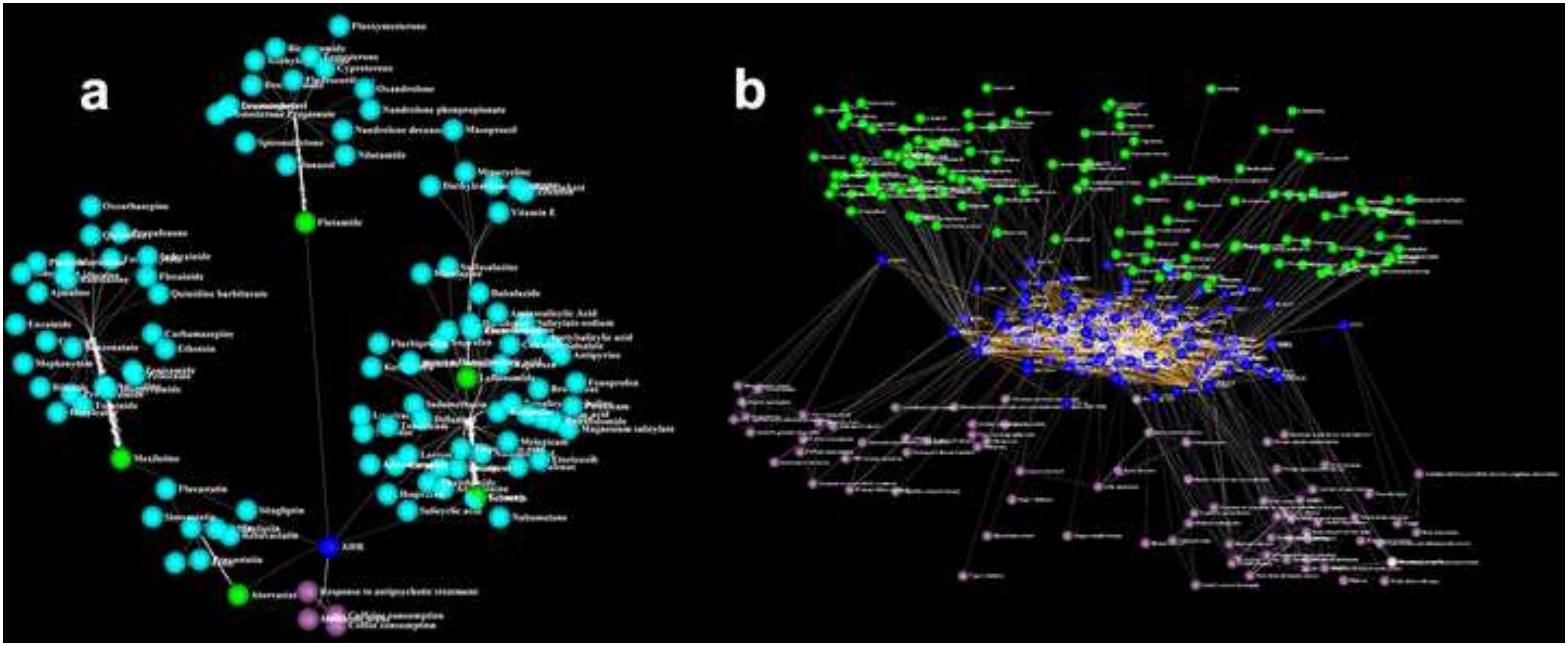

Click here to download Figure Figure-6Gumus.tif

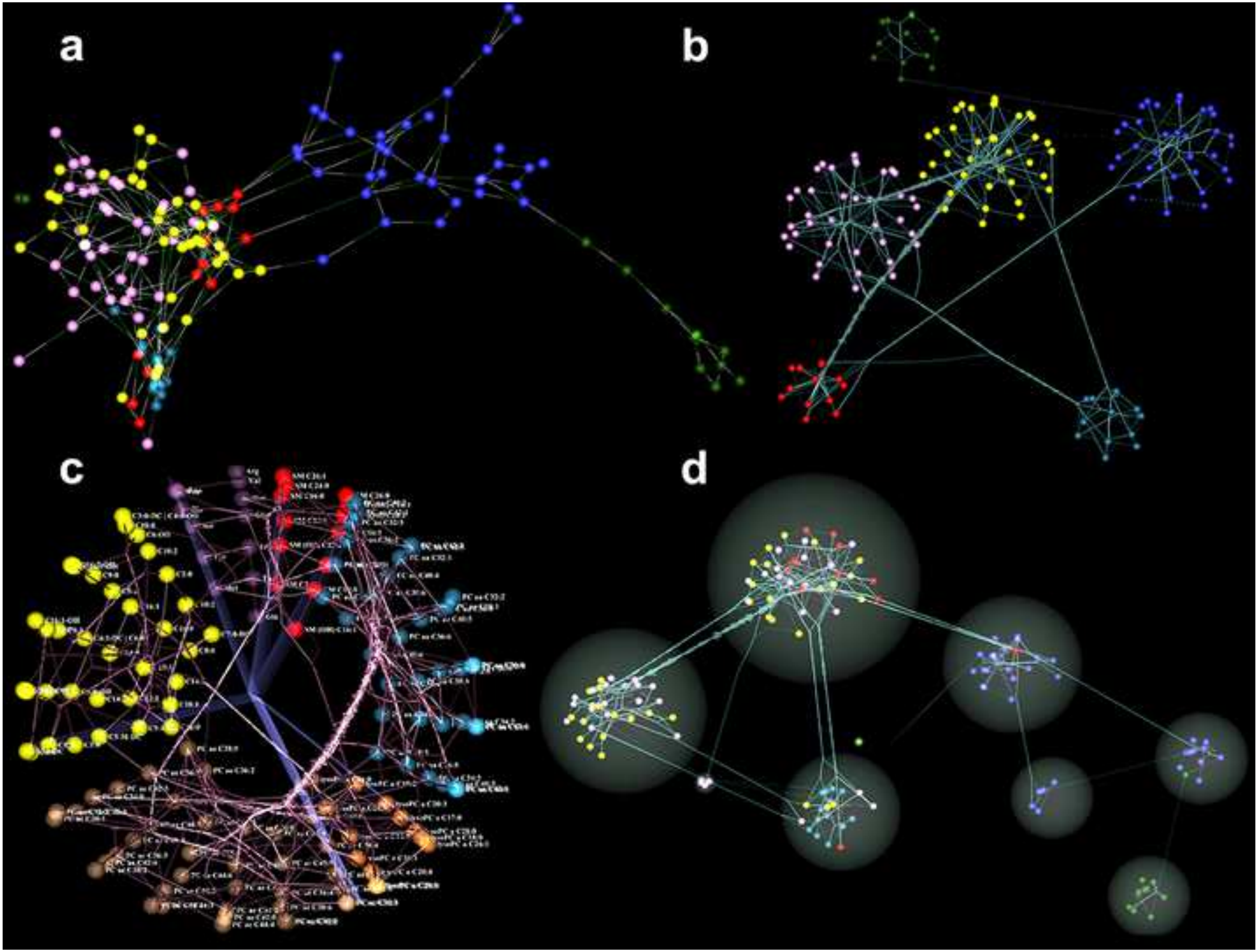

Supplementary Video 1

Click here to access/download
Supplementary Material
Supplementary Video-1Gumus.avi

Supplementary Video 2

Click here to access/download
Supplementary Material
Supplementary Video-2Gumus.avi

Supplementary Video 3

Click here to access/download
Supplementary Material
Supplementary Video-3Gumus.avi

Supplementary Video 4

Click here to access/download
Supplementary Material
Supplementary Video-4Gumus.avi

Supplementary Figures and Tables

Click here to access/download
Supplementary Material
iCAVE_SupplementaryFiguresandTables_Jan2017.pdf

Click here to access/download
Supplementary Material
iCAVE_installation_and_users_manual_May2016_ZHG.pdf

Mount Sinai

Zeynep H. Gumus, Ph.D.
Assistant Professor, Department of Genetics and Genomic Sciences
Member- Institute of Genomics and Multiscale Biology
One Gustave L. Levy Place, Box 1498
New York, NY 10029-6574

T 267-243-3530
F 646-537-8660
zeynep.gumus@mssm.edu

February 6, 2017

Dear GigaScience Editorial Board:

We respectfully submit for your consideration as a Research Paper our manuscript *iCAVE: an open source tool for visualizing biomolecular networks in 3D, stereoscopic 3D and immersive 3D.*

Visualizations of complex biomolecular interaction networks are critical in multiple systems. The volume of data represented in biomolecular interaction networks is growing at an unprecedented rate due to increasing prevalence of high-throughput experimental techniques. While studies of systems consisting of thousands of biomolecules are now routine, currently available visualization tools do not scale well with large datasets. The problem is compounded with recent sequencing technologies that yield massive data. In order to achieve better understandings of such complex processes, it is important to maximally integrate data across multiple dimensions, pushing the limits of current visualization tools. Clearly, there is a strong need for new complex, heterogeneous data visualization solutions.

In our manuscript, we present a new integrative visualization platform, interactome-CAVE (iCAVE) for visualizing large and complex networks in 3D. Users can explore networks (i) in 3D using a desktop; (ii) in stereoscopic 3D using 3D-vision glasses and a desktop; (iii) in immersive 3D within a CAVE-type environment. iCAVE incorporates several layout algorithms to automatically generate 3D visualizations that solve the scalability limitations of traditional representations. Built-in network topology analyses enable effective representations that maximize understanding of the underlying network structures of large, dense, layered or clustered networks. A user can perform simultaneous integrative visualizations of multiple database resources utilizing directionality, weight or other network properties with different layout, textures, colors or densities. Portable between desktops and CAVE environments, iCAVE provides a freely available resource for gaining novel insights from complex HT datasets.

iCAVE addresses an existing need in the user community and has already been employed in several studies (e.g. Khurana *et al,* ***Science***, 2013; Fromer *et al.* ***Nature Neuroscience****, 2016;* Yan *et al,* ***Cell Systems****, 2016*). Overall, we describe a novel and user-friendly complex network visualization software that we can greatly empower investigators from diverse biomedical fields in gaining novel insights from massive, heterogenous datasets, and therefore of general interest to the broad readership of ***GigaScience***.

Appropriate Reviewers would include:

Mark Gerstein, **PhD** [Expert on large networks in bioinformatics and biomedicine]
Professor of Biomedical Informatics
Molecular Biophysics & Biochemistry and Computer Science
Yale University, New Heaven, CT, USA
Email: mark@gersteinlab.org

Cagatay Demiralp, PhD [Expert in data and network visualizations; immersive visualizations]

Visualization Researcher
IBM Thomas J Watson Research Center
Yorktown Heights, NY, USA
Email: cagatay@cs.stanford.edu

Radu Jianu, PhD [Expert in data and network visualizations; data interaction in immersive environments; biological networks]
Lecturer
Department of Computer Science
City University, London, GB
Email: radu.jianu@city.ac.uk

David Laidlaw, PhD [Expert in multidimensional and biological data visualizations; immersive environments; networks]
Professor of Computer Science
Department of Computer Science
Brown University
Email: dhl@cs.brown.edu

On behalf of all authors, thank you for your kind consideration,

Zeynep H Gumus, PhD
Assistant Professor
Department of Genetics and Genomic Sciences
Icahn Institute for Genomics and Multiscale Biology
Mount Sinai School of Medicine

Adjunct Assistant Professor
Department of Medicine
Weill Cornell Medical College

Adjunct Member
Drug Research Center
Koc University, Istanbul, Turkey
