# Supplementary material for: iCAVE: an open source tool for visualizing biomolecular networks in 3D, stereoscopic 3D and immersive 3D
Source: Gigascience. 2017 Jul 15;6(8):1–13. doi: 10.1093/gigascience/gix054 (PMC5554349; doi:10.1093/gigascience/gix054)
Supplement: Reviewer-1_Original-Submission-(Attachment).pdf [file gix054_Reviewer-1_Original-Submission-(Attachment).pdf]

Graph theory has bee widely applied in bioinformatics for data analysis. The physical coordinates are omitted for plotting a network graph in 2D. iCAVE is designed for 3D visualization/manipulation of network graphs:
1. 3D visualization of network graphs is achieved on a 2D computer screen
2. The 3D visualization is expanded in virtual reality (VR): VR glasses and VR caves
3. Open source. Adding new algorithms for graph layout and clustering is allowed.
The searchers using graph theory in their work are going to benefit from iCAVE.

The layout of the paper is not quite clear, a little bit confusing:
The layout of the paper is: Background, Results, Illustrative Examples, Discussion, Methods, User interface, Network Topological Properties, and Layout Algorithms. Putting the Results and Illustrative Examples just after the introduction section is not common.
Two consecutive subsections *Graph Clustering To Identify Network Motifs* and *Layout Options for Cluster Visualization* are lying between Illustrative Examples and Discussion. It's strange.
The last two paragraphs *Edge-Betweenness (EB) Clustering Algorithm* and *Edge bundling algorithm* are under the section Layout Algorithms. Also the graph clustering algorithms are described in the subsection *Graph Clustering To Identify Network Motifs* after the Example 3.
The content of the paper needs a clear arrangement for reads to understand easily. A short paragraph describe the overall layout would be very nice.

There are a few questions not quite clear in the paper:
1. What are the requirements for VR glasses and caves? Could the authors provide the information of the VR glasses and caves that they have had iCAVE tests?
2. Currently a node can be located manually. Can a node be located among thousands of nodes with the information of its name or physical coordinates?
3. Are the visual contents (network graph and software interface) on computer screen and in VR (glasses/cave) exactly the same? What about the operations? A clear description about the difference among computer screen, VR glasses and VR cave is preferred.
4. Can the VR touch controller replace the mouse-keyboard input totally? If the VR touch controller could not fulfill all functions of iCAVE, users need to take off and put on classes quite a few times.
5. Each node of a 3D graph can be poisoned with its physical coordinates. Also a graph with multi layers is available iCAVE. So is it possible to make the shape of a node resemble the shape of the object it stands for? For example a node symbolizing a protein, this node could be composed of (sub)-nodes symbolizing atoms or residues, then the node has the shape of the protein. If it's possible, it would be very interesting to see an example.
6. The paragraph *3. Hemispherical layout:*
In the beginning (line 53) i ϵ V, while in the end (line 6) 0° ≤ □ ≤ # □□ □□□□□.
Should 0° be 1?
