# Supplementary material for: iCAVE: an open source tool for visualizing biomolecular networks in 3D, stereoscopic 3D and immersive 3D
Source: Gigascience. 2017 Jul 15;6(8):1–13. doi: 10.1093/gigascience/gix054 (PMC5554349; doi:10.1093/gigascience/gix054)
Supplement: Suppelment Materials [file gix054_Supp.zip › iCAVE_installation_and_users_manual_May2017_Gigascience.pdf]

Installing iCAVE (runs on Linux, Unix and Mac)

© Vaja Liluashvili, Selim Kalayci and Zeynep H. Gümüş.

- Prerequisites for Mac:

  The following packages are not readily installed in a Mac. You need to install them before you install iCAVE:

  - XCode - https://developer.apple.com/xcode/downloads
  - If you do not have X11 window system on your Mac, you also need to install:

    XQuartz - http://www.xquartz.org
  - libjpeg - http://mac-dev-env.patrickbougie.com/libjpeg/
  - libz - http://www.zlib.net/
  - libpng - http://www.libpng.org/pub/png/libpng.html

- Download iCAVE from our website, save to your local machine and execute the following commands in terminal:

  - cd into the directory into which you downloaded iCAVEinstall.tar.gz
  - tar –xvzf iCAVEinstall.tar.gz
  - cd iCAVEinstall (this directory is referred as $iCAVE_HOME in the rest of this document)
  - ./install.sh (if it does not work, try chmod +rwx ./install.sh)
  - source setup.bash

That's it!

If you get an error: "error while loading shared libraries …" you need to type in your terminal:
- export LD_LIBRARY_PATH="{$iCAVE_HOME/Vrui-3.1/lib }"

- To test whether iCAVE installation was successful on your machine, you can display a sample network provided within iCAVE package. To perform this:
  - Open your X Window terminal (e.g. xterm)
  - source setup.bash
  - cd $iCAVE_HOME/vrnet
  - vrnetview -Gsample.txt -O10

iCAVE input file formats

Listed below are the basic input file formats for iCAVE graphs. Some sample data files are available at your $iCAVE_HOME/vrnet/importFiles folder. We have aimed to make the data input both simple and flexible. The formats below are quite flexible in that they allow different data types to be input and visualized. The user has considerable control over the node attributes such as color, size, texture, scale, annotation, as well as edge color, size, weight and directionality.

- iCAVE input files are text files representing columns of data
- Each data column is separated by a tab.
- Each node should have a unique identifier.
- Options such as weight, color, size, texture, scale, cluster and directionality are added after the columns that define nodes and pair-wise edges.

Simple multicolumn format (.txt, .csv):
This is the easiest format for data input. The format allows for a full range of nodes and edges to be created from a simple column format that can be prepared in a simple spreadsheet such as Excel or Numbers. The basic format for such networks is as follows.
Nodes are defined after the header line:
#nodes name

After the nodes are defined, all pair-wise edges are <u>defined and listed in the same file</u> underneath the nodes, starting with the header line:
#edges node1 node2

Here is the simplest iCAVE input file example:

```
# nodes name
nodeA
nodeB
nodeC
#edges node1 node2
nodeA	nodeB
nodeA	nodeC
```

Note that the columns are tab-separated.

Nodes with user-defined color, size, texture options

When using node color, size or texture options (attributes), then the file should begin with:

#nodes name attributeNames

xyz (optional) – If you have a network with pre-defined (x, y, z) positions of the network nodes (i.e. the network represents a protein or a brain structure, or you simply have an adjacency matrix), you can use this attribute. The (x, y, z) positions can take real or integer values. Example:

```
#nodes	name	xyz
nodeA	0.1	0.4	0.3
```

Node Colors: You have two separate options for node coloring. You can either choose to make all the nodes a color of your choice, or you can specify each node color separately. If you do not specify a color option, iCAVE will use a default color for your nodes.

node_color (optional) – If you want <u>single, unified coloring for all the nodes</u> in your network, use this attribute. You need to specify this attribute only once, on the same line that begins with “#nodes”.

When you specify this attribute, in the next tab, you need to provide the name of the color you want. For the full-range of color names that you can choose from, you can refer to the file:
$iCAVE_HOME/vrnet/ColorChart.pdf.

An example header where the color is user-defined to be green for all nodes is:
#nodes name node_color green
nodeA
nodeB
…

color_code (optional) – If you want to specify different colors for various nodes in your network, use this attribute. After you specify this attribute on the same line that begins with "nodes", you need to associate each node with the name of the color you want for that node. For the full-range of color names that you can choose from, you can refer to the file:
$iCAVE_HOME/vrnet/ColorChart.pdf.

Here is a header example with this option- where you choose the first node magenta and second navy:

#nodes name color_code
nodeA magenta
nodeB navy

texture_type (optional) – If you want the node to have a texture, you can specify a texture type using the following integer values: 0: dashed node, 1: polka dot, 2: triangles in the circle, 3: striped, 4: stars
Here is an example:

#nodes name texture_type
nodeA 0
nodeB 1

You can also mix and match all these! In fact, we hope you can make use of any and all the attributes!

Advanced node attributes:

Nodes in Clusters. You may want to color different members of a user-defined cluster differently. In that case, when your input file includes clusters, then use the following option:

cluster (optional) - integer for if you have pre-described clusters. Example:

#nodes name cluster
nodeA 0
nodeB 1

Nodes with gene expression fold change values: Use this when you want to input gene expression values from your experiments directly into iCAVE, where the node color reflects the increase or decrease in expression of the gene the node represents in that particular RNA-seq or microarray experiment.

foldChange (optional) – any real value number. Example:

#nodes name foldChange
nodeA 0
nodeB 1.611670994

node_scale (optional) – any real value number- will change the scale of the nodes relative to each other. Example:

#nodes name node_scale

nodeA 0
nodeB 10

labels_only_scaled_nodes (optional) – when used in combination with *node_scale* option, only the labels of scaled nodes (the ones with a different value than 0) are displayed. Example:

#nodes name node_scale labels_only_scaled_nodes
nodeA 0
nodeB 10

layer_code (optional) – you can specify the layout of your nodes to be organized in several distinct layers with this option. Example:

#nodes name layer_code
nodeA 1
nodeB 2
nodeC 2

category (optional) – You can use this option to differentiate between different classes of nodes. If using superpathway, following are the codes that you can use : 0 - protein, 1 - complex, 2 - rna , 3 - mirna , 4 - family, 5 - abstract. If visualizing metabolic network, following are the codes that you can use: 0 - unclassified, 1 - amino acid, 2 - acyl_carnitiness, 3 - diacyl-PC, 4 - lyso-PC, 5 - acyl-alkyl-PC, 6 - sphingomyeline.

You can also use this option to color the nodes with preset colors rather than using the color_code option. Available color codes are as follows: 0: Orange, 1: Green, 2: Blue, 3: Magenta, 4:Gray, 5: Deep Pink, 6: Red, 7: Cyan. If you choose a code between 8 - 19, a random color will be assigned to those nodes. Example:

#nodes name category
nodeA 1
nodeB 2

showNodeProperty (optional) – if no *node scale* option is provided and you specify *showNodeProperty* option, each node is highlighted at an intensity in accordance with its number of neighbors. Example:

#nodes name showNodeProperty
nodeA
nodeB

Edges with user-defined color, direction, thickness, weight options:

At its simplest input file format, you can define the edges by stating the pairwise connections with the edges section header (right under you have defined all the nodes):

#edges node1 node2

Optional attributes such as edge color, weight, thickness and direction can be added as follows:
#edges node1 node2 attributes
nodeA nodeB attribute
nodeC NodeD attribute

isDirected (optional) – if the network is directed, in the line #edges write isDirected. Example:

#edges node1 node2 isDirected

edge_color (optional) – If you want a single, unified coloring for all the edges in your network, use this attribute. You need to specify this attribute only once, on the same line that begins with “#edges”.
When you use this attribute, you need to provide specify color you want in the next tab. For the full-range of color names you can choose from, you can refer to the file: $iCAVE_HOME/vrnet/ColorChart.pdf. Example:

#edges node1 node2 edge_color darkred

color_code (optional) – If you want to specify distinct colors for different edges in your network, use this attribute. After you specify this attribute on the same line that begins with “edges”, you need to associate each edge with the name of the color you want for that edge. For the full-range of color names that you can choose from, you can refer to the file: $iCAVE_HOME/vrnet/ColorChart.pdf.

Here is a header example with this option- where you choose the first edge cyan and second red:

#edges node1 node2 color_code
nodeA nodeB cyan
nodeB nodeC red

weight (optional) – If you want to specify weights for the edges in your network, use this attribute. After you specify this attribute on the same line that begins with “edges”, you need to associate each edge with the weight you want for that edge. Weight values can be positive or negative real numbers. Negative values are translated into the visualization as the color of the edge being red/white stripes. Positive values are translated into the visualization as the color of the edge being blue/white stripes. The absolute value of the weight is translated as the frequency of the stripes. Larger absolute weight values correspond to denser stripes. Example:

#edges node1 node2 weight
nodeA nodeB 0.35
nodeB nodeC -1.32

thickness (optional) – If you want to specify thickness for the edges in your network, use this attribute. After you specify this attribute on the same line that begins with “edges”, you need to associate each edge with the thickness you want for that edge. Thickness values can be positive real numbers. Larger thickness values, correspond to thicker edges. Example:

#edges node1 node2 thickness
nodeA nodeB 2.35
nodeB nodeC 4

hideEdgeWeight (optional) – To hide edge weights in visualization. Example:

#edges node1 node2 weight hideEdgeWeight

showNodeProperty (optional) – If you don't provide node information but still want to see node property (as explained in Advanced node attributes) specify showNodeProperty in #edges line. Example:

#edges node1 node2 weight showNodeProperty
nodeA nodeB 0.35
nodeB nodeC -1.32

strength (optional) – You can use this option to differentiate between edges when visualizing GWAS studies. Following are the codes used during GWAS studies: -1 - default, 0 - if two nodes interact, 1 - if they share a disease, 2 - if they interact and share a disease.

You can also use this option to color the edges with preset colors rather than using the *color_code* option. Available color codes are as follows: 0: Orange, 1: Green, 2: Magenta, 3: Gray, 4:Red, 5: Cyan, 6: Deep Pink. If you choose a code between 7 - 19, a random color will be assigned to those edges. Example:

```
#edges node1   node2   strength
nodeA  nodeB   1
nodeB  nodeC   2
```

interactionType (optional) – You can specify the type of interaction between two nodes using this option. After you specify this option in #edges line, for each edge, you can pick one of the following interaction types: -a> : activation, -a| : inhibition, -t> : transcription activation, -t| : transcription inhibition. Example:

```
#edges node1   node2   weight  interactionType
nodeA  nodeB   0.35    -a>
nodeB  nodeC   -1.32   -a|
```

iCAVE input file conversion from/to other formats:

You can convert your network input file from some commonly used format into iCAVE format, and visa versa.

To convert network input files in Cytoscape and Gephi formats to iCAVE input files, use these scripts in folder $iCAVE_HOME/utils :

- sif_to_icave.sh: converts Cytoscape SIF format files to iCAVE format.
- csv_to_icave.sh: converts .csv format files used in Cytoscape and Gephi to iCAVE format.

To convert network input files in iCAVE format to Cytoscape and Gephi formats, use these scripts in folder $iCAVE_Home/utils:

- icave_to_cyto.sh: converts network files in iCAVE format to Cytoscape format.
- icave_to_gephi.sh: converts network files in iCAVE format to Gephi format.

Input parameter to these scripts is simply the name of the file you want to convert. Output file will be generated and placed in the current working directory.

Example:

$ sh sif_to_icave.sh networkA.sif

will generate output file networkA.sif.txt, which can then be used as an input file to iCAVE.

NOTE: csv_to_icave.sh script assumes that your .csv file has a header line. If your .csv file does NOT have a header line, please use the --no-header flag.

Running iCAVE

Before running iCAVE, we suggest watching iCAVE video tutorial: https://vimeo.com/154868345.

Changing the 'stereo' option

If your machine is stereo-enabled, and you have 3-D glasses, you can see the networks in immersive 3-D. To use this option, go to $iCAVE_HOME, and then run command:

- sh stereo.sh

If you want to disable the stereo option, go to $iCAVE_HOME, and then run command:

- sh no-stereo.sh

A video tutorial on how to turn on/off immersive 3D in iCAVE is at: https://vimeo.org/154868346.

Please start your X Window terminal and go to $iCAVE_HOME/vrnet.

Different arguments can be passed to iCAVE input file, as listed above. When in the vrnet library, you have multiple options in running your graphs, depending on what you would like to do. The simplest case is when you already have a list of nodes and pairwise edge connections, and you would like to visualize the network they form. Here, you do not need to query databases to generate the network. The mandatory and optional commands for this case are below:

Mandatory arguments:

-GFILE_NAME (example: -Gpin_ave100.txt)	Specify file name. This file should be located under $iCAVE_HOME/vrnet/importFiles folder. Note that there is no space between G and your FILE_NAME.
-O10	Specify that the file is in iCAVE format (i.e. with node and edge attributes as defined above)

Run command:

- vrnetview -Gfilename.txt -Ol0 -l0 (optional argument - force directed layout)

Optional arguments:

-lLayoutALgorithm (example: -l0)	Specify layout algorithm to use (if you are not importing the coordinates).
-BbackgroundColor (example: -B0)	Specify background color (default is black). For white background, specify -B1.

If the known networks have nodes with fold change values:

Mandatory arguments:

-MFILE_NAME (example: Mggm_cn.txt)	Specify the import file name (node/edge attributes). File should be located at $iCAVE_HOME/vrnet/pathwayFile
-O3	You have to use -O3 option, as this tells iCAVE that it should parse connectivity Matrix with fold change values (be careful, this format is specific only for ggm_cn.txt, if you want to import your own connectivity matrix, the parsing algorithm needs to be adjusted)

Optional arguments:

-lLayoutALgorithm (example: -l0)	Specify which layout algorithm to use

Querying databases that are included in iCAVE

Single gene query:

Mandatory arguments:

-gGENE_NAME (example: -gMYL9)	Specify the name of gene of interest
-nSHELL_NUMBER (example: -n2)	Specify degree of interactions

Example command: vrnetview -gMYL9 -n2

Optional arguments:

-lLayoutALgorithm (example: -l0) (l as in lion)	specify layout algorithm:<br>-l0 = force directed,<br>-l1 = weighted force-directed (if you have edge weights),<br>-l2 = hemispherical,<br>-l3 = semantic_levels,<br>-l4 = linLog,<br>-l5 = weighted linLog,<br>-l6 = hybrid force directed (default),<br>-l7 = OpenOrd,<br>-l8 = YifanHu
-dDATABASE_ID	Specify which database should be used.<br>-d0 = HPRD (default),<br>-d1 = superPathway,<br>-d2 = Combodatabase (intact with HPRD)

Example command: vrnetview -gMYL9 -n2 -l2 -d2

User supplied gene lists from HT experiments

Mandatory arguments

-LYOUR_GENE_LIST_FILE_NAME<br>(Ex: -Lsmoke , -Lcopd, -Lphantom)	Specify a user-defined gene list file. As an example, you can run the built in examples (smoke, copd, phantom).<br>**Querying HPRD or COMBO for gene lists:**<br>1) Create a file containing only gene names YOURGENEData.txt and copy to folder $iCAVE_HOME/vrnet/queringLists (ex: smokeData.txt).<br>2) Depending on which you plan to query, go to folder $iCAVE_HOME/vrnet/hprd or $iCAVE_HOME/vrnet/comboDatabase. Create a file **importYOURFILENAME.sql**. (e.g. see hprd/importSmokeData.sql for importing data in HPRD; comboDatabase/importSmokeData.sql for importing in combo Database). Simply copy one of these two example files to importYOURFILENAME.sql<br>Querying gene lists in databases with fold changes:<br>1) Create a file containing your genes and fold changes in tab separated format YOURGENEData.txt(see file queringLists/cCancerData.txt)<br>2) Create a file importYOURGENE.sql. Write a query for importing your data (see the file hprd/importcCancerData.sql for importing data in HPRD, and comboDatabase/ importcCancerData.sql for importing in combo Database). You can copy this file in importYOURGENE.sql and all you need to change are the names: change ccancerData with YOURGENEData and "cCancer" , with the name you would like to use with -L option.

Optional arguments:

-lLayoutALgorithm (example: -l0)	Specify which layout algorithm should be used
-dDATABASE_ID	Specify which database should be used. –d0 = HPRD (default), -d1 = SuperPathway (this database is very sparse) -d2 = Combodatabase.
-DDIFF_EXPRESSION_NAME<br>(example: -Dsmoker_expression)	To use fold change values during visualization. (red is up; green is down)

Disease-associated gene catalogue based on GWAS studies vs. drugs

Mandatory arguments:

-gGENE_NAME (example: -gMYL9)	Specify the name of gene of interest
-w (e.g -w1, or -w2)	-w0 = don't use GWAS(default). If you want to visualize GWAS data, always use -w1 (to query STITCH drug database), or -w2 (to query DrugBank drug database). You cannot use layoutAlgorithms option, since we use special layout for GWAS data.

Optional arguments:

-p1	If enabled, iCAVE will visualize a network, showing the gene of interest, interacting drugs and predicted drugs. For all interacting drugs, other interacting genes are found, and their drug interactions are the predicted ones.

User provided gene list clusters with gene-gene interactions query

Mandatory arguments:

-cCLUSTER_NAME (Example: -csmokecl)	specify the name of cluster of interest. We have smokecluster imported in databases and it is possible to import others as follows:<br>1) Create a file containing only gene names and corresponding cluster numbers YOURGENESData.txt (see the file queringLists/smokeCluster.txt).<br>2) Create a file importYOURDATANAME.sql. Write a query for importing your data (see hprd/importSmokeCluster.sql for importing data in HPRD, and comboDatabase/importClusterData.sql for importing in combo Database). You can simply copy this file in importYOURDATANAME.sql and change the names: change smokeCluster with YOURGENEData and smokecl, with the name you would like to use with -c option.
-dDATABASE_ID	Specify which database should be used. -d0 = HPRD (default), -d2 = intact, -d3 = Combodatabase (intact with HPRD). Superpathway cannot be used here.

Importing a previously saved network layout

Mandatory arguments:

-l7	If importing saved layout, always use -l7, which indicates that node positions are already known
-sLAYOUT_ID (e.g. -s2)	specify the ID of the saved network layout

Example command: vrnetview -s2 -l7

There is one extra option -R which can always be used. -R0 tells iCAVE not to use drawLists, since they don't work in the CAVE yet. Don't change this option unless you are in CAVE, and always use -R0.

iCAVE Output Formats:

Snapshot:

The output generated by "Save Snapshot" operation is saved in $iCAVE_HOME/vrnet folder as "Snapshot.png". (If you already have a "Snapshot.png" file, don't forget to move or rename it, otherwise it will be overwritten)

Animation:

The output generated by "Save (Hi-Res) Rotation As Movie" operation is saved in $iCAVE_HOME/vrnet folder as "animation.gif" file. (If you already have an "animation.gif" file, don't forget to move or rename it, otherwise it will be overwritten)

Network Measures:

You can display different graph properties by clicking the menu item "Show General Measures". Please note that some of the properties are meaningful only for selected nodes. If you want, you can save the distributions of network measures as diagrams by clicking the "Save Diagrams" button. You can find these diagrams in the $iCAVE_HOME/vrnet/diagrams folder.

Clustering:

The resulting clustering information from applying each clustering method (Betweenness Centrality, LinLog, MCL), besides being shown on the network visualization, is saved in a corresponding text file in the $iCAVE_HOME/vrnet/clusteringResults folder.

Interactive user options during iCAVE display sessions

Navigation:

Press left mouse button and move the mouse for rotation
Press "z" and move the mouse for translation
Press left mouse button + "z" and move the mouse for zoom in and out
Or use the rotation mouse wheel, for zooming in and out

Showing iCAVE main menu:

Press right mouse button to see the main menu.

Creating Locator:

Since some of iCAVE's options are sensible after selecting a node, locator creation is necessary. To create a locator press a number from 0-9, or ctrl+right mouse button. Move to locator -> Screen Locator. (Other menu options are not important for us.)

Selecting and deselecting a node:

Create a locator. To select a node, keep holding the ctrl key and right-click once on the node. If it is difficult to select desired node, then use 3d to 2d projection. To deselect, keep holding the ctrl key and right-click once again.

Moving nodes

Create a locator. Enable the option "Move Methods -> Move Nodes". Keep holding the ctrl key. Right-click on a node once, move the node to desired position and right-click again.

Deleting nodes

Create a locator. Enable the option "Allow deleting nodes". Keep holding the ctrl key. Right-click on a node and it gets deleted.

Deleting edges

Create a locator. Enable the option "Allow deleting edges". Keep holding the ctrl key. Right-click on starting node of the edge you wish to delete. The node gets highlighted. Now right-click on ending node. If nothing happens, that means you clicked on a node which is not the ending node of the edge. Try it again, if necessary rotate the model.

Saving animation

Enable the option "Rotate model". Choose appropriate speed. Make sure the model does not leave the window. Execute "Save rotation as movie". The output is saved in $iCAVE_HOME/vrnet folder as animation.gif file. (If you already have an "animation.gif" file, don't forget to rename it, otherwise it will be overwritten)

Creating 3D Circos

If the input file specifies categories for the nodes in the network, you can create 3D Circos layout of the network by selecting the option "Network Algorithms -> Create 3D Circos". In 3D Circos layout, the nodes are arranged in 3D space as in hemispherical layout, where the most connected node is located at the center of the hemisphere. Also, if clustering information is available (user-defined or topology-based), the hemisphere is also sliced with (pie-like) panels that correspond to separate clusters.

iCAVE main menu interactive options

1. Layout algorithms → Select different layout algorithms implemented in iCAVE (does not work if the node coordinates have been specified in the input file)
2. Clustering algorithms → Execute a specific clustering algorithm (works only for undirected networks)
3. Reset Navigation → Reset all navigation transformations
4. Bundle Edges → Execute edge bundling algorithm
5. 3D to 2D projection → Projects the nodes onto screen plane
6. Save Snapshot → Create snapshot from network. Choose appropriate zoom factor and execute this algorithm. The output is saved in $iCAVE_HOME/vrnet folder as “Snapshot.png” (If you already have a “Snapshot.png” file, don't forget to rename it, otherwise it will be overwritten)
7. Select Node → Allows the user to highlight a node of interest. A window gets created, with names of nodes. Usage: click on one of the names, and the node with this name gets selected
8. Deselect Node → Reverse the highlighting process
9. Save Layout → Saves the current network layout (arrangement of nodes) for later import. This operation returns a unique ‘layout ID’. You need to specify this ‘layout ID’ to import the saved network layout later.
10. Show Labels → Show the names of nodes
11. Show General Measures → Shows different graph properties. Some of the properties are only for selected nodes. Usage: Create a locator and select a Node. You can save distributions as diagrams in folder $iCAVE_HOME/vrnet/diagrams by clicking “Save Diagrams”
12. Move Nodes → Allows the user to move selected node to desired location.
13. Allow Deleting Nodes → Allows the user to delete nodes
14. Allow Deleting Edges → Allows the user to delete edges
15. Separate Different Categories → Separates different “categories” specified in the network. Category layout is in force-directed, while inside each category nodes are ordered in hemispherical layout.
16. Create 3D Circos → Arranges the nodes (based either on node category or clusters) in 3D space as in hemispherical layout, where the most connected node is located at the center of the hemisphere. If this is done based on clustering information, the hemisphere is also sliced with (pie-like) panels that correspond to separate clusters.
17. Show Additional Information → Only sensible when "SUPERPATHWAY" is being visualized. It shows the whole name of selected node, its membership in family and complex. Usage: Create a locator and select a Node. Activate this option
18. Show GWAS Information → Show information about SNPs when GWAS data is being visualized
19. Show Neighbors List → Shows the interaction partners of selected Node. Usage: Create a locator, if not already created, and select a Node. The appearing window is a clickable list. Click on one of the names in the list, and the node with this name gets selected
20. Show Single Nodes → Show nodes which don't have any interactions
21. Show Netview Info → This shows the clicked coordinates. Usage: Create a locator, click in the space (with right mouse button) and activate this option
22. Rotate Model → Rotates the model. The user can save this rotation as .gif file. The output will be saved in $iCAVE_HOME/vrnet folder.
23. Reset Network → Reverse edge-bundling, clustering, 2D projection, node/edge move&delete operations
24. Adjust Node/Edge Size → Adjust the size of the nodes and/or edges using the slider
25. Adjust Colors → Adjust the color (via setting the RGB values) of nodes, edges, clusters using the slider
26. Adjust Labels Size → Adjust the font-size of the labels using the slider

Please contact zeynep.gumus@mssm.edu if you have any questions.
