# Supplementary material for: iCAVE: an open source tool for visualizing biomolecular networks in 3D, stereoscopic 3D and immersive 3D
Source: Gigascience. 2017 Jul 15;6(8):1–13. doi: 10.1093/gigascience/gix054 (PMC5554349; doi:10.1093/gigascience/gix054)
Supplement: Suppelment Materials [file gix054_Supp.zip › iCAVE_SupplementaryFiguresandTables_Jan2017.pdf]

## Supplementary Figures and Tables

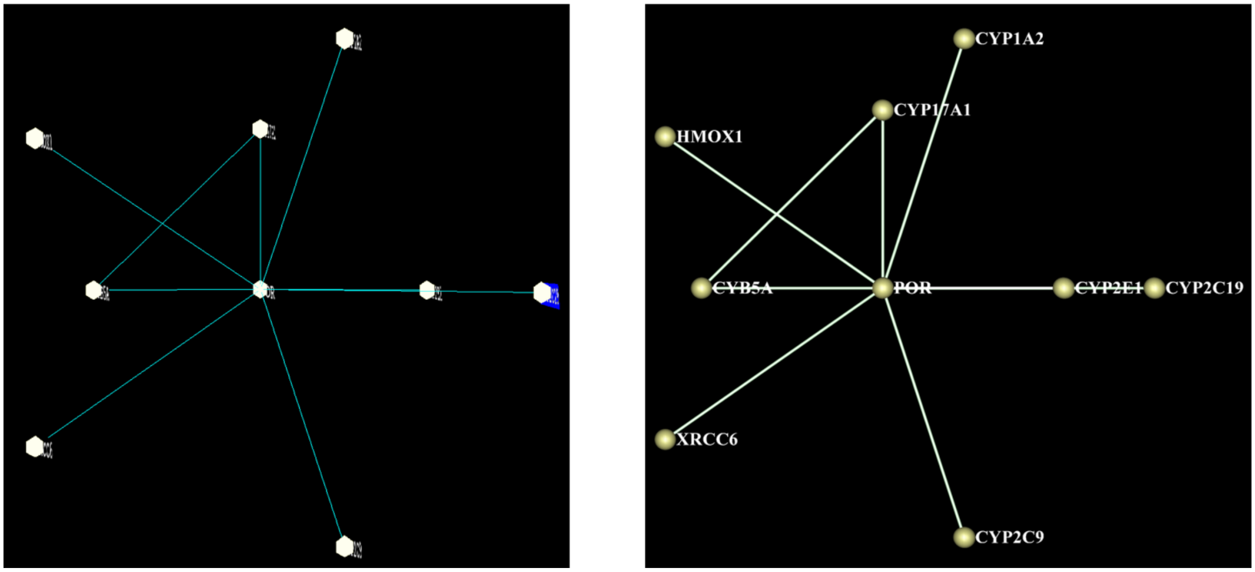


**Supplementary Figure 1.** Labels created with VRUI versus iCAVE. Left: VRUI intern method; Right: texture mapping.

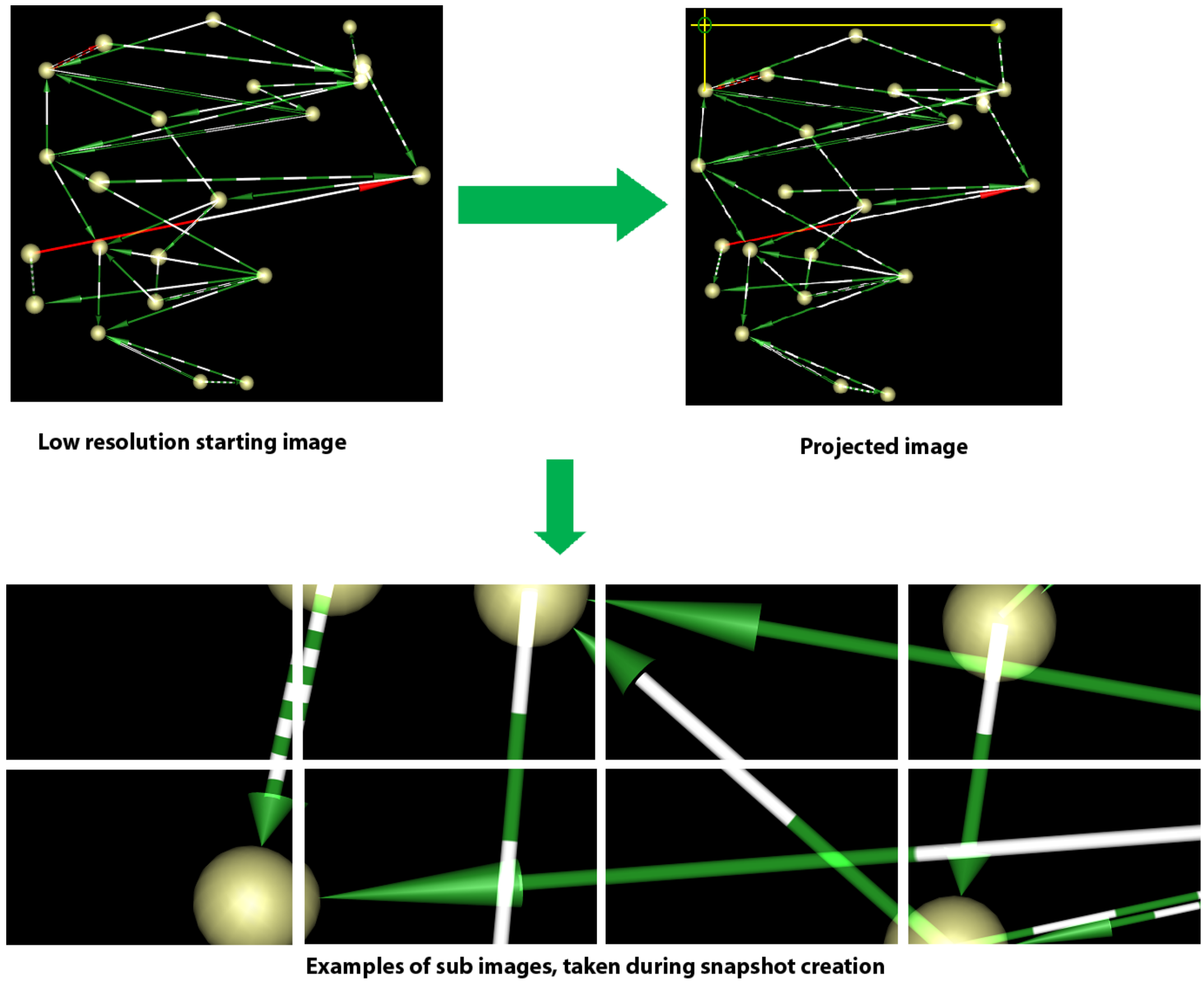


**Supplementary Figure 2.** High-resolution image creation steps. Upper left image is a low-resolution snapshot created with common tools (800*600). This picture was projected into 2D screen plane (upper-right) and upper-left corner location was recorded as shown in upper right. 170 sub-images were taken to create a 18490*17560 resolution image (only 8 are shown in the bottom panels).

**Supplementary Table 1**. iCAVE currently provides a limited database within the COMBO repository for quick queries. Because of iCAVE's modular structure, users (who have some programming experience) can populate COMBO with additional databases.

| COMBO Database | Version | # of entities (genes, drugs, diseases) | # of interactions |
|---|---|---|---|
| **Protein-protein interaction** | | | |
| HPRD Human Protein Reference (http://www.hprd.org ) | 07/2010 | 19,651 (genes) | 39,240 |
| intAct Molecular interaction (http://www.ebi.ac.uk/intact) | 03/2012 | 29,665 (genes) | 116,522 (mapped) |
| **Disease-associated gene variant databases** | | | |
| GWAS (http://genome.gov/gwastudies ) | 05/2012 | 5,234 (diseases) | 11,199 |
| **Drug-target databases** | | | |
| STITCH (http://stitch.embl.de ) | V3 | 2,467 (drugs)<br>19,686 (genes) | 39,117 |
| DRUGBANK (http://drugbank.ca ) | 06/2012 | 1,578 (drugs) | 4,235 |
| **Pathway databases** | | | |
| SuperPathway (Personal communication with Josh Stuart, UCSD) | 11/2011 | 20,076 (proteins)<br>8,897 (complexes)<br>1,866 (families)<br>491 (abstracts)<br>122 (RNAs)<br>15 (miRNAs) | 8,345 (directed interactions) |

**Supplementary Table 2.** iCAVE user-interactive menu options.

| | |
|---|---|
| **Show Netview Info** | display clicked 3D coordinates |
| **Show General Measurements** | calculate network topological properties (e.g. hubs, centrality) |
| **Show Labels** | display node names |
| **Show Single Nodes** | display nodes with no interactions |
| **Show Neighbors List** | highlight all interacting neighbors of selected node |
| **Show Additional Information** | display the name of selected component of interest, and its membership in a family or a complex (if available) |
| **Show GWAS Information** | display GWAS information if provided |
| **Reset Options** | Choose from: Reset navigation; Network |
| **Move Methods** | Choose from: Move Nodes; Layers; Clusters |
| **Allow Deleting Nodes** | Allow deleting of selected nodes |
| **Allow Deleting Edges** | Allow deleting of selected edges |
| **Rotate Model** | set the rotation rate along X/Y/Z axis |
| **Adjust Colors** | Adjust node or edge color |
| **Adjust Label Size** | Use pop-up slider to change label size |
| **Adjust Circos Size** | Use pop-up slider to change hemisphere size |
| **Layout Algorithms** | Choose from: Force Directed; Weighted Force Directed; Hemisphere; Semantic Levels; LinLog; Hybrid Force Directed |
| **Clustering Algorithms** | Choose from: Betweenness Centrality; LinLog; MCL |
| **Network Algorithms** | Choose from: Bundle Edges; 3D to 2D projections; Save Snapshot; Select Nodes; Deselect Nodes; Separate Small Network; Create 3D Circos; Separate different categories |
| **Save Layout** | save current network layout in sql database format (.db) |
| **Save Zoom As Movie** | Generate movie of current network layout |
