# Supplementary material for: iCAVE: an open source tool for visualizing biomolecular networks in 3D, stereoscopic 3D and immersive 3D
Source: Gigascience. 2017 Jul 15;6(8):1–13. doi: 10.1093/gigascience/gix054 (PMC5554349; doi:10.1093/gigascience/gix054)
Supplement: Response-to-Reviewer-Comments_Original-Submission.pdf [file gix054_Response-to-Reviewer-Comments_Original-Submission.pdf]

Author's Response To Reviewer Comments

Dear Dr. Edmunds:

We would like to thank both Reviewers for the positive feedback, comments and suggestions. We modified the software, user's manual and manuscript based on the comments of the Reviewers. All changes are highlighted in yellow in the manuscript and user's manual. Our point-by-point response to Reviewers remarks as well as the changes and clarifications that we made are as follows:

Reviewer 1:
Graph theory has bee widely applied in bioinformatics for data analysis. The physical coordinates are omitted for plotting a network graph in 2D. iCAVE is designed for 3D visualization/manipulation of network graphs:
1. 3D visualization of network graphs is achieved on a 2D computer screen
2. The 3D visualization is expanded in virtual reality (VR): VR glasses and VR caves
3. Open source. Adding new algorithms for graph layout and clustering is allowed.
The searchers using graph theory in their work are going to benefit from iCAVE.

Reviewer 1 Remarks:

1. Reviewer Remark: The layout of the paper is not quite clear, a little bit confusing:
The layout of the paper is: Background, Results, Illustrative Examples, Discussion, Methods, User interface, Network Topological Properties, and Layout Algorithms.
Putting the Results and Illustrative Examples just after the introduction section is not common. Two consecutive subsections Graph Clustering To Identify Network Motifs and Layout Options for Cluster Visualization are lying between Illustrative Examples and Discussion. It's strange. The last two paragraphs Edge-Betweenness (EB) Clustering Algorithm and Edge bundling algorithm are under the section Layout Algorithms. Also the graph clustering algorithms are described in the subsection Graph Clustering To Identify Network Motifs after the Example 3. The content of the paper needs a clear arrangement for reads to understand easily. A short paragraph describe the overall layout would be very nice.

Response: We have re-organized the manuscript sections and we have added a short paragraph that describes the overall manuscript layout to the end of the Introduction section.

Reviewer 1 Questions:
1. Reviewer Question: What are the requirements for VR glasses and caves? Could the authors provide the information of the VR glasses and caves that they have had iCAVE tests?

Response: As we have described in the Methods section, iCAVE needs to be installed on the files system of the CAVE computer, the projectors of which should be able to view Vrui: (http://idav.ucdavis.edu/~okreylos/ResDev/Vrui/LinkDownload.html)

We have tested iCAVE in the CAVE facility of Weill Cornell Medical College in New York, USA. This is an immersive visualization facility driving Christie Mirage HD3K projectors

(Christie Digital Systems USA).

As we have described in the methods section, iCAVE also works with stereo 3D glasses connected to any stereo-enabled computer.

2. Reviewer Question: Currently a node can be located manually. Can a node be located among thousands of nodes with the information of its name or physical coordinates?

Response: Yes, the user can click on the ‘Network Algorithms/Select Node’ button, and pick the name of the node, which is then highlighted.

3. Reviewer Question: Are the visual contents (network graph and software interface) on computer screen and in VR (glasses/cave) exactly the same? What about the operations? A clear description about the difference among computer screen, VR glasses and VR cave is preferred.

Response: Yes, essentially as we have described in Figure 1, the visual contents are the same in a CAVE environment as in the computer display.

4. Reviewer Question: Can the VR touch controller replace the mouse-keyboard input totally? If the VR touch controller could not fulfill any functions of iCAVE, users need to take off and put on classes quite a few times.

Response: Yes, as depicted in Figure 1, in a CAVE facility, a wand replaces all the mouse/keyboard input without losing any capabilities and interact with the content without interruption.

5. Reviewer Question: Each node of a 3D graph can be poisoned with its physical coordinates. Also a graph with multi layers is available iCAVE. So is it possible to make the shape of a node resemble the shape of the object it stands for? For example, a node symbolizing a protein, this node could be composed of (sub)-nodes, symbolizing atoms or residues, then the node has the shape of the protein. If it is possible, it would be very interesting to see in example.

Response: We thank the reviewer for the excellent suggestion. We are indeed planning to implement such a feature in the upcoming version of iCAVE.

6. Reviewer Question: The paragraph 3. Hemispherical layout, in the beginning (line 53):

Response: Thank you for pointing this out. Formula is now fixed in the manuscript.

Reviewer #2:
Last year I wrote an extensive review about network visualization emphasizing in the fact that modern technologies such as Virtual Reality must be used.
I am happily surprised, shortly after my review to see a tool such as iCave addressing exactly this point and therefore I highly recommend it for publication.
I played with this tool and certainly there are UI things to improve.

Reviewer 2 Remarks:

Reviewer Remark 1: Living in the big-data era, I would suggest OpenOrd and Yifan Hu (Gephi Layouts) to be additionally added as they scale very well for larger networks.

Response: We thank the reviewer for this excellent suggestion. In response, we have adapted and extended OpenOrd to 3D and implemented it into iCAVE as an additional layout titled "Simulated Annealing Force Directed". In addition, we have also extended Yifan Hu's layout to 3D space and have implemented this layout into iCAVE as "Coarsened Force Directed".
We added a discussion on the details of these layouts in the manuscript in pages 8 and 20.

Reviewer Remark 2: Also, I would recommend authors to emphasize on the export functionality and store networks in various formats to be easily loaded from Cytoscape and Gephi. It would be convenient to be able to save the status of the network (coordinates, colors etc) and view it with other 2D/3D tools. That would be nice to have in this version.

Response: This is another excellent suggestion. For input formats, we have implemented utility scripts to convert networks from iCAVE input files to Cytoscape and Gephi formats. We also implemented scripts to convert .sif and .csv files to iCAVE-ready input files. These scripts are now available in the iCAVE package under the utils directory.
We have added a section to the user's manual on how to convert file formats (Page 7, highlighted).
For output formats, we are also in the process of saving the status of networks in SVG and GML formats to better exchange graphs among different visualization software. This functionality will be available in the next version.

Reviewer Remark 3: Another thing that is missing is a more detailed analysis about its performance and scalability. BioLayoutExpress people have put lots of effort in this. Please elaborate more on icave's scalability in this version of the manuscript.

Response: This is definitely an important aspect for a computational tool (specifically a 3-D visualization tool for large networks). Such a study requires conducting an extensive analysis about the performance and scalability of iCAVE to display and perform various operations (e.g. calculate a certain layout, perform edge bundling, etc.) on networks of various sizes and topologies. We believe such as detailed study is beyond the scope of the current current manuscript, which is a coincise and focused introduction of the functionalities of iCAVE. We are currently conducting detailed and systematic analyses which we plan to publish as a separate manuscript.
At the same time, we believe this is an important point to provide introductory information on in the current manuscript and therefore we have added a descriptive paragraph on performance and scalability. (Page 14).

Reviewer Remark 4: Also selecting multiple nodes with a rectangle, cutting a network into subnetworks, arrange multiple networks at the same time as well as having functionality like in Cytoscape for example where one can perform clustering and store it as a separate network/view would be a plus.

Response: Unfortunately, performing certain operations in 3D (selecting a region, cutting network, etc.) can become erratic and impractical for the user. However, we are in the process of implementing a module similar to the “Data Laboratory” functionality in Gephi to help facilitate such activities and save the results accordingly.

Reviewer Remark 5: A Satellite view would be also of a great help.

Response: This is a very useful suggestion. We will incorporate this functionality in the next version.

Reviewer Remark 6: An undo button would make a difference.

Response: In iCAVE menu, we already have a ‘Reset Network’ button that corresponds to the functionality of an Undo button in most cases (e.g. edge bundling, clustering, 2d projection, move nodes, etc).

Reviewer Remark 7: These are just some of my suggestions and I would not like authors to address all of them in the current version of the tools as I see the usefulness for tools like icave to become available.
Authors claim that icave is modular enough to add functionality and I am sure that they know very well which should be the next steps.
I think icave's modularity must become a priority so that users can build their own plugins. This is mainly how Cytoscape became popular in Biology at the moment hosting >300 plugins.

Response: We are in the process of developing a “Developer’s Guide” document to explain potential developers how they can incorporate their own algorithms/modifications in the base software. We will publish and update this document accordingly in iCAVE website.

Reviewer Remark 8: Finally, a windows version would be very nice to have in the future.

Response: Unfortunately due to the technologies and libraries used in iCAVE, Windows version would require almost completely new and different architecture specific to Windows machines. Instead, we are in the process of designing and developing a Web version of iCAVE.

Reviewer Remark 9: The manuscript is very nicely written and very easy to follow and does not need editing. Once again, I am very pleased with icave manuscript and would definitely recommend it for publication even in its current version.

Response: Thank you!

Thanks for your consideration,

Sincerely Yours,

Zeynep H Gumus, PhD (on behalf of all authors)
