# Supplementary material for: iCAVE: an open source tool for visualizing biomolecular networks in 3D, stereoscopic 3D and immersive 3D
Source: Gigascience. 2017 Jul 15;6(8):1–13. doi: 10.1093/gigascience/gix054 (PMC5554349; doi:10.1093/gigascience/gix054)
Supplement: Reviewer-2-Report-(Original-Submission).pdf [file gix054_Reviewer-2-Report-(Original-Submission).pdf]

Reviewer Report

Title: iCAVE: an open source tool for visualizing biomolecular networks in 3D, stereoscopic 3D and immersive 3D

Version: Original Submission **Date:** 2/26/2017

Reviewer name: Georgios Pavlopoulos

Reviewer Comments to Author:

Last year I wrote an extensive review about network visualization emphasizing in the fact that modern technologies such as Virtual Reality must be used.
I am happily surprised, shortly after my review to see a tool such as iCave addressing exactly this point and therefore I highly recommend it for publication.

I played with this tool and certainly there are UI things to improve.

Living in the big-data era, I would suggest OpenOrd and Yifan Hu (Gephi Layouts) to be additionally added as they scale very well for larger networks.

Also, I would recommend authors to emphasize on the export functionality and store networks in various formats to be easily loaded from Cytoscape and Gephi. It would be convenient to be able to save the status of the network (coordinates, colors etc) and view it with other 2D/3D tools. That would be nice to have in this version.

Another thing that is missing is a more detailed analysis about its performance and scalability. BioLayoutExpress people have put lots of effort in this. Please elaborate more on icave's scalability in this version of the manuscript.

Also selecting multiple nodes with a rectangle, cutting a network into subnetworks, arrange multiple networks at the same time as well as having functionality like in Cytoscape for example where one can perform clustering and store it as a separate network/view would be a plus.

A Satellite view would be also of a great help.

An undo button would make a difference.

These are just some of my suggestions and I would not like authors to address all of them in the current version of the tools as I see the usefulness for tools like icave to become available.

Authors claim that icave is modular enough to add functionality and I am sure that they know very well which should be the next steps.

I think icave's modularity must become a priority so that users can build their own plugins. This is mainly how Cytoscape became popular in Biology at the moment hosting >300 plugins.

Finally, a windows version would be very nice to have in the future.

The manuscript is very nicely written and very easy to follow and does not need editing.

Once again, I am very pleased with icave manuscript and would definitely recommend it for publication even in its current version.

Methods

Are the methods appropriate to the aims of the study, are they well described, and are necessary controls included? Yes

Conclusions

Are the conclusions adequately supported by the data shown? Yes

Reporting Standards

Does the manuscript adhere to the journal's guidelines on minimum standards of reporting? YesChoose an item.

Statistics

Are you able to assess all statistics in the manuscript, including the appropriateness of statistical tests used? No, and I do not feel adequately qualified to assess the statistics.

Quality of Written English

Please indicate the quality of language in the manuscript: Acceptable

Declaration of Competing Interests

Please complete a declaration of competing interests, considering the following questions:

- Have you in the past five years received reimbursements, fees, funding, or salary from an organisation that may in any way gain or lose financially from the publication of this manuscript, either now or in the future?
- Do you hold any stocks or shares in an organisation that may in any way gain or lose financially from the publication of this manuscript, either now or in the future?

- Do you hold or are you currently applying for any patents relating to the content of the manuscript?
- Have you received reimbursements, fees, funding, or salary from an organization that holds or has applied for patents relating to the content of the manuscript?
- Do you have any other financial competing interests?
- Do you have any non-financial competing interests in relation to this paper?

If you can answer no to all of the above, write 'I declare that I have no competing interests' below. If your reply is yes to any, please give details below.

I declare that I have no competing interests

I agree to the open peer review policy of the journal. I understand that my name will be included on my report to the authors and, if the manuscript is accepted for publication, my named report including any attachments I upload will be posted on the website along with the authors' responses. I agree for my report to be made available under an Open Access Creative Commons CC-BY license (http://creativecommons.org/licenses/by/4.0/). I understand that any comments which I do not wish to be included in my named report can be included as confidential comments to the editors, which will not be published.

I agree to the open peer review policy of the journal

To further support our reviewers, we have joined with Publons, where you can gain additional credit to further highlight your hard work (see: https://publons.com/journal/530/gigascience). On publication of this paper, your review will be automatically added to Publons, you can then choose whether or not to claim your Publons credit. I understand this statement.

Yes
